# Prevalence of chlamydia, gonorrhoea, and trichomoniasis among male and female general populations in sub-Saharan Africa from 2000 to 2024: a systematic review and meta-regression analysis

**DOI:** 10.1016/j.eclinm.2025.103210

**Published:** 2025-04-24

**Authors:** Julia Michalow, Lauren Hall, Jane Rowley, Rebecca L. Anderson, Quinton Hayre, R. Matthew Chico, Olanrewaju Edun, Jesse Knight, Salome Kuchukhidze, Evidence Majaya, Domonique M. Reed, Oliver Stevens, Magdalene K. Walters, Remco P.H. Peters, Anne Cori, Marie-Claude Boily, Jeffrey W. Imai-Eaton

**Affiliations:** a MRC Centre for Global Infectious Disease Analysis, School of Public Health, Imperial College London, London, United Kingdom; b Department of Global HIV, Hepatitis and Sexually Transmitted Infections Programmes, World Health Organization, Geneva, Switzerland; c Center for Communicable Disease Dynamics, Department of Epidemiology, Harvard T.H. Chan School of Public Health, Boston, MA, USA; d Department of Disease Control, Faculty of Infectious and Tropical Diseases, London School of Hygiene & Tropical Medicine, London, United Kingdom; e Centre for Infectious Disease Epidemiology and Research, School of Public Health and Family Medicine, University of Cape Town, Cape Town, South Africa

**Keywords:** Chlamydia, Gonorrhoea, Trichomoniasis, Sexually transmitted infection, Surveillance

## Abstract

**Background:**

Sub-Saharan Africa (SSA) has the highest sexually transmitted infection (STI) prevalence globally, but information about trends and geographic variation is limited by sparse aetiologic studies, particularly among men. This systematic review assessed chlamydia, gonorrhoea, and trichomoniasis prevalence by sex, sub-region, and year, and estimated male-to-female prevalence ratios in SSA.

**Methods:**

We searched Embase, MEDLINE, Global Health, PubMed, and African Index Medicus for studies measuring STI prevalence among general populations from 1 January 2000, to 17 September 2024. We adjusted observations for diagnostic test performance and used log-binomial mixed-effects meta-regressions to estimate prevalence trends and sex-prevalence ratios. The study was registered with PROSPERO (CRD42023420384).

**Findings:**

Of 5202 records identified, we included 211 studies from 28 countries. In 2020, estimated prevalence among 15–49-year-olds in SSA for chlamydia was 6.9% (95% CI: 5.2–8.7%, n = 169 observations) among females and 4.2% (3.0–5.5%, n = 33) among males, gonorrhoea was 1.8% (1.1–2.5%, n = 171) and 1.5% (0.8–2.2%, n = 31), and trichomoniasis was 7.6% (5.1–10.2%, n = 188) and 1.7% (1.1–2.4%, n = 19). Male-to-female ratios were 0.61 (0.53–0.71) for chlamydia, 0.81 (0.61–1.09) for gonorrhoea, and 0.23 (0.18–0.28) for trichomoniasis. From 2010 to 2020, chlamydia prevalence increased by 34.5% (11.1–62.9%) in SSA, while gonorrhoea and trichomoniasis trends were not statistically significant. Chlamydia and gonorrhoea prevalence were highest in Southern and Eastern Africa, whereas trichomoniasis was similar across sub-regions.

**Interpretation:**

SSA has a high, geographically varied STI burden, with increasing prevalence of chlamydia. Region-specific sex-prevalence ratios differed from existing global ratios and should be considered in future burden estimates. Enhanced sex-stratified surveillance is crucial to guide national programmes and reduce STI prevalence in SSA.

**Funding:**

Gates Foundation, 10.13039/501100000761Imperial College London, 10.13039/100000002NIH, 10.13039/100014013UKRI.

**Research in context**

**Evidence before this study**

The World Health Organization (WHO) Global Health Sector Strategy on Sexually Transmitted Infections (STIs) aims to reduce gonorrhoea incidence by 90%, and chlamydia and trichomoniasis incidence by 50% by 2030. Sub-Saharan Africa has a disproportionately high prevalence of these three curable STIs, but prevalence patterns and trends are poorly characterised, particularly among men. We searched PubMed using the terms (“*Chlamydia trachomatis*” [MeSH] or “Chlamydia Infections” [MeSH] or “Gonorrhea” [MeSH] or “*Trichomonas vaginalis*” [MeSH] or “Trichomonas Infections” [MeSH]) AND “Review” [Publication type] to identify systematic reviews and meta-analyses on the prevalence of these infections in sub-Saharan Africa. Previous reviews estimated high STI prevalence in sub-Saharan Africa, particularly among key populations and populations living with HIV. However, these reviews were typically restricted to specific sub-populations, did not analyse temporal trends, or had limited geographic stratification. Previously published estimates by WHO and the Global Burden of Disease study relied on global sex-prevalence ratios to account for the scarcity of data among men. No previous reviews had examined sub-regional prevalence patterns and trends among both male and female general populations in sub-Saharan Africa, hindering evidence-based planning toward the WHO 2030 targets.

**Added value of this study**

We synthesized data from 211 studies across 28 sub-Saharan African countries to estimate the prevalence of chlamydia, gonorrhoea, and trichomoniasis by sex and sub-region between 2000 and 2024. We systematically evaluated sub-regional variation and temporal trends in prevalence and established empirical sex-prevalence ratios, while accounting for different diagnostic strategies across studies. We found persistently high prevalence of all three STIs, which exceeded global prevalence. There was substantial geographic variation in chlamydia and gonorrhoea prevalence in the region, which mirrored patterns of HIV prevalence, while trichomoniasis prevalence was similar across sub-regions. Chlamydia prevalence has increased over the past two decades, while there was no evidence of changes in gonorrhoea and trichomoniasis prevalence over time. Our analysis established region-specific sex-prevalence ratios, which differed from global ratios used in existing estimates by WHO and the Global Burden of Disease study.

**Implications of all the available evidence**

Increasing chlamydia prevalence in sub-Saharan Africa and sustained gonorrhoea and trichomoniasis prevalence highlight the need for enhanced STI prevention and control efforts. Geographic overlaps between STI and HIV burden support WHO policy recommendations for service integration, particularly in high-burden areas. This approach, combined with strategic intervention deployment, such as new-to-market diagnostics and therapeutic agents, could maximise the impact of available resources. Our region-specific sex-prevalence ratios should be incorporated in future burden estimates to better reflect local epidemiologic variations when data are lacking. There is a critical need to strengthen sex-stratified surveillance to monitor control strategies and progress toward WHO targets.

## Introduction

Sexually transmitted infections (STIs) pose a substantial global health burden through acute urogenital conditions, complications such as pelvic inflammatory disease and infertility, and increased risk of HIV acquisition and transmission.^1^ The World Health Organization (WHO) has established targets to reduce STI incidence by 2030, which requires ambitious scale-up of national prevention and control programmes, particularly in sub-Saharan Africa where STI prevalence is highest globally.^1^ However, monitoring and optimising these programmes is challenging due to high rates of asymptomatic infection, variable health-seeking behaviour, limited diagnostic capacity necessitating syndromic management, and weak surveillance systems.^2^ STI prevalence estimates are crucial for evaluating progress toward WHO targets but are hindered by infrequent population-representative studies, especially among men.^3^

Estimates of curable STI prevalence in sub-Saharan Africa are limited. WHO publishes global and regional prevalence and incidence estimates for curable STIs, including chlamydia (*Chlamydia trachomatis*, CT), gonorrhoea (*Neisseria gonorrhoeae*, NG), and trichomoniasis (*Trichomonas vaginalis*, TV), approximately every four years.^4^, ^5^, ^6^, ^7^, ^8^, ^9^ These focus on global geographic regions and do not report sub-regional variation or temporal trends. The Global Burden of Disease (GBD) study generates global, regional, and national prevalence and incidence trends for several STIs.^10^ Both estimation approaches depend heavily on available data and underlying model assumptions. Due to limited data among men, WHO and GBD have derived male-to-female prevalence ratios from global data and applied these in most regions to support male prevalence estimates.^5^^,^^8^^,^^10^ Although WHO reviews its global ratios prior to each estimation round, values have not been updated since 1999 for trichomoniasis and 2005 for chlamydia and gonorrhoea.^5^^,^^8^ Additionally, several systematic reviews have assessed the prevalence of curable STIs in sub-Saharan Africa among pregnant women,^11^^,^^12^ women participating in HIV prevention trials,^13^ or women living with HIV,^14^ but their generalisability beyond the specified populations is limited and these reviews have not examined changes in STI prevalence over time.

We therefore conducted a systematic review of studies on chlamydia, gonorrhoea, and trichomoniasis prevalence among adults considered representative of the general population (i.e., not conducted among groups identified as key populations, as defined in WHO guidelines on HIV, STIs, and viral hepatitis^1^) in sub-Saharan Africa from 2000 to 2024. We performed meta-analyses to assess variations in STI prevalence by sex, sub-region, and over time. To inform future regional and global STI burden estimation efforts, we derived updated male-to-female prevalence ratios specific to sub-Saharan Africa.

## Methods

### Search strategy

We systematically searched Embase (Ovid), MEDLINE (Ovid), Global Health (Ovid), PubMed, and African Index Medicus from 1 January 2000 to 17 September 2024 for studies assessing the prevalence of chlamydia, gonorrhoea, and trichomoniasis in sub-Saharan Africa. Search term domains included relevant terms and synonyms for “sexually transmitted infections” and “sub-Saharan Africa” (Supplementary Table S1). We also searched citations from relevant published systematic reviews.^7^, ^8^, ^9^^,^^11^, ^12^, ^13^, ^14^, ^15^, ^16^, ^17^, ^18^, ^19^, ^20^

### Eligibility criteria and study selection

We included studies reporting sex-stratified empirical data on the prevalence of chlamydia, gonorrhoea, or trichomoniasis among adults from the general population in sub-Saharan Africa between 2000 and 2024. We considered studies to represent the general population if they enrolled participants from antenatal care (ANC), family planning, or gynaecology clinics; primary healthcare or other outpatient services; educational institutions; or community venues (e.g., social spaces or places of worship); or recruited participants for HIV or STI prevention trials (baseline data only) or population-representative surveys. Studies were eligible regardless of participants’ symptom status, though we excluded those that exclusively enrolled symptomatic individuals or individuals seeking treatment for STIs or infertility. We excluded studies with participants enrolled exclusively from populations living with HIV or key populations.

We included studies with participants aged 12 years or older who were tested using internationally recognised aetiologic diagnostic tests with urogenital specimens (urine, urethral, or cervicovaginal samples), and excluded studies that only collected pharyngeal or rectal samples. We also excluded case reports, commentaries, longitudinal and randomised controlled studies reporting only post-baseline outcomes, and studies with fewer than 15 participants. We used the UN M49 standard to define sub-Saharan Africa and its sub-regions (Supplementary Table S2).^21^

Search results were managed and de-duplicated using Covidence systematic review software (Veritas Health Innovation, Melbourne, Australia).^22^ Researchers (DMR, EM, JM, JWI-E, JK, LH, MKW, OE, OS, QH, RLA, SK) independently double-screened title and abstract records for eligibility and assessed full text articles for inclusion. A third reviewer resolved discrepancies.

### Data extraction

Researchers (JM, LH, RLA, QH) independently double-extracted prevalence observations (number of individuals with a positive result of the total number tested) for each study with stratification by country, sex, and population group, as available. We also recorded information on study characteristics, participant characteristics, and diagnostic methodology (Supplementary Table S3). Discrepancies were resolved through consensus.

If outcomes from the same study were reported in multiple articles, we preferentially extracted observations from the largest sample or, if samples were the same size, the first published article. For studies collecting samples from multiple anatomical sites, we extracted only urogenital results. If multiple diagnostic tests were conducted, we extracted outcomes from the test and sample type with highest sensitivity and specificity for pathogen identification (Supplementary Table S4). We preferentially extracted non-stratified rather than HIV-stratified outcomes, if both were reported.

### Data analysis

To adjust study prevalence observations for imperfect diagnostic test performance, we collated sensitivity and specificity values for each test category from published literature (Supplementary Table S4)^5^^,^^23^^,^^24^ and used a Bayesian approach to estimate prevalence in each study population (Supplementary Text S1).^25^^,^^26^

For studies that did not report data collection dates (n = 26), we imputed the midpoint year of data collection by subtracting the median publication lag from the publication year (three years, based on the difference between the data collection midpoint year and year of publication among studies with dates reported). For one study spanning multiple sub-regions without country- or region-specific data reported,^27^ we classified the study sub-region according to where most participants had been recruited.

To estimate sub-regional trends in sex-specific STI prevalence in sub-Saharan Africa, we fit log-binomial mixed-effects meta-regressions for each pathogen. Fixed effects included year (midpoint of data collection), study population (ANC attendees, family planning clinic attendees, gynaecology clinic attendees, primary healthcare or other outpatient facility attendees, students, community members recruited from non-clinical venues, HIV/STI prevention trial participants, and population-representative survey participants), sex (female, male), age group (younger adults 12–25 years, all adults ≥12 years), HIV status (HIV negative, non-stratified HIV status), diagnostic test (nucleic acid amplification test [NAAT], culture, direct fluorescent antibody, enzyme immunosorbent assay, rapid antigen test, wet mount), sub-region (Western and Central Africa [WCA], Eastern Africa [EA], or Southern Africa [SA]), year and sub-region interaction term, and sex and sub-region interaction term. A year and sex interaction term was not included due to limited observations of male prevalence. We combined Western and Central Africa due to limited prevalence observations in Central Africa. Study-level random intercepts allowed for between-study heterogeneity. Fitted models were used to predict the prevalence of each infection among male and female adults by sub-region between 2000 and 2024, using reference categories for covariates in the model (study population: ANC attendees, HIV status: non-stratified, diagnostic test: NAAT). We report estimates for sub-regions and all of sub-Saharan Africa for 2020. Sub-Saharan Africa estimates were obtained by weighting sub-regional predictions with sex-matched population size for adults aged 15–49 years in 2020, according to UN World Population Prospects 2024.^28^ Standard errors were determined using the delta method.^29^

To assess sex differences in prevalence, we estimated male-to-female prevalence ratios using observations from all studies (between-study ratios) and for the subset of studies providing estimates for both sexes (within-study ratios). We estimated between-study ratios using previously described meta-regression models. We determined within-study ratios using the same meta-regression approach, but without fixed effects for population group and an interaction term for sex and sub-region, due to limited observations. We also derived pooled within-study ratios without adjusting for any covariates.

### Sensitivity analyses and risk of bias

We assessed sensitivity of prevalence estimates, trends, and male-to-female prevalence-ratios to adjustments for diagnostic performance by comparing results from models using all available observations vs. only NAAT-diagnosed observations, with and without accounting for test sensitivity and specificity.

We assessed risk of bias by adapting the Joanna Briggs Institute critical appraisal tool for prevalence studies. Studies were classified as lower or higher risk of selection bias (sampling method, participant characterisation), measurement bias (diagnostic method consistency), and poor precision (sample size adequacy; Supplementary Table S5).^30^ We extended our primary meta-regressions to include fixed effects for each criterion to assess their influence on prevalence estimates.

Unless indicated otherwise, all main results reflect observations adjusted for diagnostic test performance. Results are presented as means with 95% confidence intervals (95% CIs). Model coefficients are presented as adjusted prevalence ratios (aPRs), with confidence intervals calculated on the log scale prior to exponentiation. Study observation heterogeneity was assessed per model as the percentage of total variance attributed to observation-level random effects.^31^ Analyses were conducted in R version 4.2.3; Bayesian diagnostic test performance adjustments used RStan version 2.26.21^32^ and meta-regression modelling used glmmTMB version 1.1.8.^33^

We registered our study with PROSPERO (CRD42023420384)^34^ and reported according to the Preferred Reporting Items for Systematic Reviews and Meta-Analyses (PRISMA) guideline.^35^ The Imperial College Research Ethics Committee approved analysis of secondary data for this study (ICREC #6365329).

### Role of funding source

The funders of the study had no role in study design, data collection, data analysis, data interpretation, or writing of the report.

## Results

### Search results and scope

**Fig. 1:**
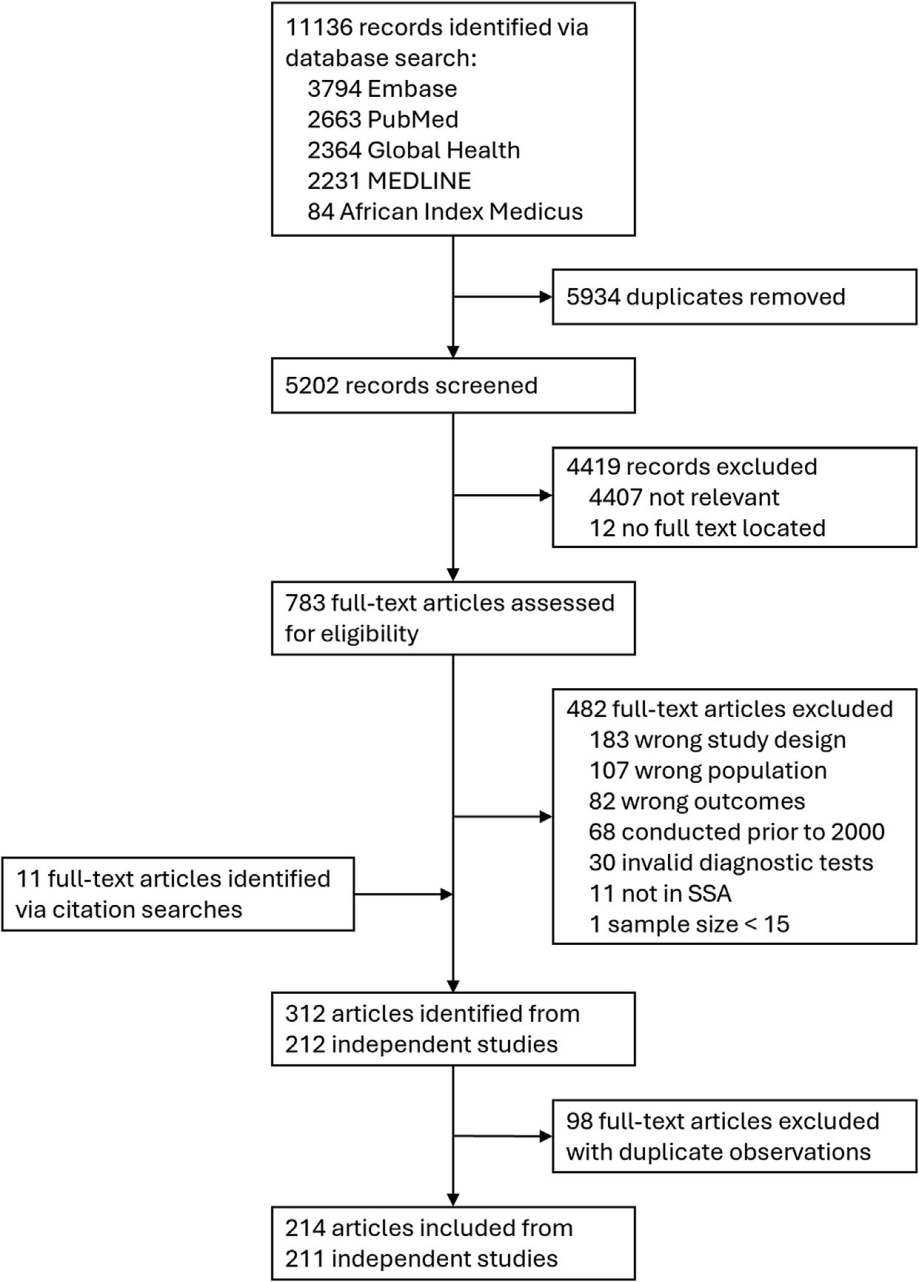
**Study selection flowchart.** Flowchart of study selection for systematic review.

We identified 11,136 records through the database search, of which 5934 were duplicates and 5202 were screened (Fig. 1). Of these, 783 full-text articles were assessed and 301 met eligibility criteria. After adding 11 articles identified through citation searching and excluding 98 articles with duplicate study observations, we included 214 articles from 211 unique studies, from which we extracted 614 prevalence observations (NG = 203, CT = 203, TV = 208; Supplementary Table S6). Most studies were in Eastern Africa (N = 90/211, 42.7%), followed by Western and Central (N = 71/211, 33.6%), and Southern (N = 59/211, 28.0%) Africa (Table 1). Studies were conducted in 28 different sub-Saharan African countries, with most in South Africa (N = 53/211, 25.1%), Nigeria (N = 38/211, 18.0%), Kenya (N = 26/211, 12.3%), United Republic of Tanzania (N = 20/211, 9.5%), and Uganda (N = 19/211, 9.0%) (Supplementary Fig. S1). Study populations were predominantly ANC attendees (N = 85/211, 40.3%), primary healthcare or outpatient department attendees (N = 30/211, 14.2%), and HIV/STI prevention trial participants (N = 24/211, 11.4%). Few studies were among population-representative survey samples (N = 11/211, 5.2%). Most studies were solely among females (N = 174/211, 82.5%). A limited number included both sexes (N = 29/211, 13.7%) or males only (N = 8/211, 3.8%). Studies were predominantly among all adults (N = 180/211, 85.3%), rather than restricted to younger adults (N = 31/211, 14.6%). Across all studies, most enrolled within the 15–49-year age range (N = 109/211, 51.7%), while others included younger and/or older participants (N = 45/211, 21.3%), reported only a minimum adult age (N = 37/211, 17.5%), or did not report age ranges (N = 20/211, 9.5%).

**Table 1:** Summary of characteristics for included studies.

Variable	Group	Chlamydia (N = 139)	Gonorrhoea (N = 140)	Trichomoniasis (N = 162)	Total (N = 211)
Sub-region^a^	Western Africa	20 (14.4%)	20 (14.3%)	51 (31.5%)	59 (28.0%)
	Central Africa^b^	10 (7.2%)	8 (5.7%)	8 (4.9%)	12 (5.7%)
	Eastern Africa	69 (49.6%)	70 (50.0%)	63 (38.9%)	90 (42.7%)
	Southern Africa	49 (35.3%)	51 (36.4%)	46 (28.4%)	59 (28.0%)
	Multiple sub-regions^c^	1 (0.7%)	1 (0.7%)	1 (0.6%)	1 (0.5%)
Study midpoint year^a^^,^^d^	2000–2004	27 (19.4%)	32 (22.9%)	34 (21.0%)	45 (21.3%)
	2005–2009	18 (12.9%)	19 (13.6%)	21 (13.0%)	27 (12.8%)
	2010–2014	35 (25.2%)	31 (22.1%)	38 (23.5%)	51 (24.2%)
	2015–2019	42 (30.2%)	41 (29.3%)	54 (33.3%)	64 (30.3%)
	2020–2024	18 (12.9%)	17 (12.1%)	15 (9.3%)	25 (11.8%)
Population group^a^	ANC attendees	39 (28.1%)	42 (30.0%)	75 (46.3%)	85 (40.3%)
	FP attendees	12 (8.6%)	12 (8.6%)	11 (6.8%)	17 (8.1%)
	GYN attendees	6 (4.3%)	8 (5.7%)	11 (6.8%)	14 (6.6%)
	PHC/OPD attendees	22 (15.8%)	22 (15.7%)	20 (12.3%)	30 (14.2%)
	Students	15 (10.8%)	11 (7.9%)	15 (9.3%)	19 (9.0%)
	Community members^e^	15 (10.8%)	16 (11.4%)	6 (3.7%)	17 (8.1%)
	HIV/STI prevention trial participants^f^	22 (15.8%)	22 (15.7%)	22 (13.6%)	24 (11.4%)
	Population-representative survey participants	10 (7.2%)	10 (7.1%)	8 (4.9%)	11 (5.2%)
Sex	Female only	106 (76.3%)	109 (77.9%)	143 (88.3%)	174 (82.5%)
	Male only	7 (5.0%)	5 (3.6%)	7 (4.3%)	8 (3.8%)
	Both sexes (stratified)	26 (18.7%)	26 (18.6%)	12 (7.4%)	29 (13.7%)
Age group^g^	All adults (12+ years)	112 (80.6%)	116 (82.9%)	140 (86.4%)	180 (85.3%)
	Younger adults (12–25 years)	27 (19.4%)	25 (17.9%)	22 (13.6%)	31 (14.7%)
HIV status^a^^,^^h^	HIV-negative	37 (26.6%)	38 (27.1%)	38 (23.5%)	46 (21.8%)
	Non-stratified	103 (74.1%)	103 (73.6%)	125 (77.2%)	166 (78.7%)

N: number of studies, ANC: antenatal care, FP: family planning, GYN: gynaecology clinic, PHC/OPD: Primary healthcare or outpatient department.

a The same study is included in more than one subcategory if it reports across different variable levels or when multiple levels are relevant.

b Western and Central Africa combined for analysis.

c Study without country- or region-specific reporting was allocated to Eastern Africa for analysis, according to where most participants were recruited.

d Midpoint year between start and end of data collection period.

e Community members recruited through non-clinical venues, such as social spaces or places of worship.

f Studies reporting baseline data among trial participants.

g Age groups are not mutually exclusive due to limitations in study reporting.

h Studies exclusively among populations living with HIV were excluded. Data were preferentially extracted and analysed without stratifying by HIV status if both were reported.

### Sexually transmitted infection prevalence

**Fig. 2:**
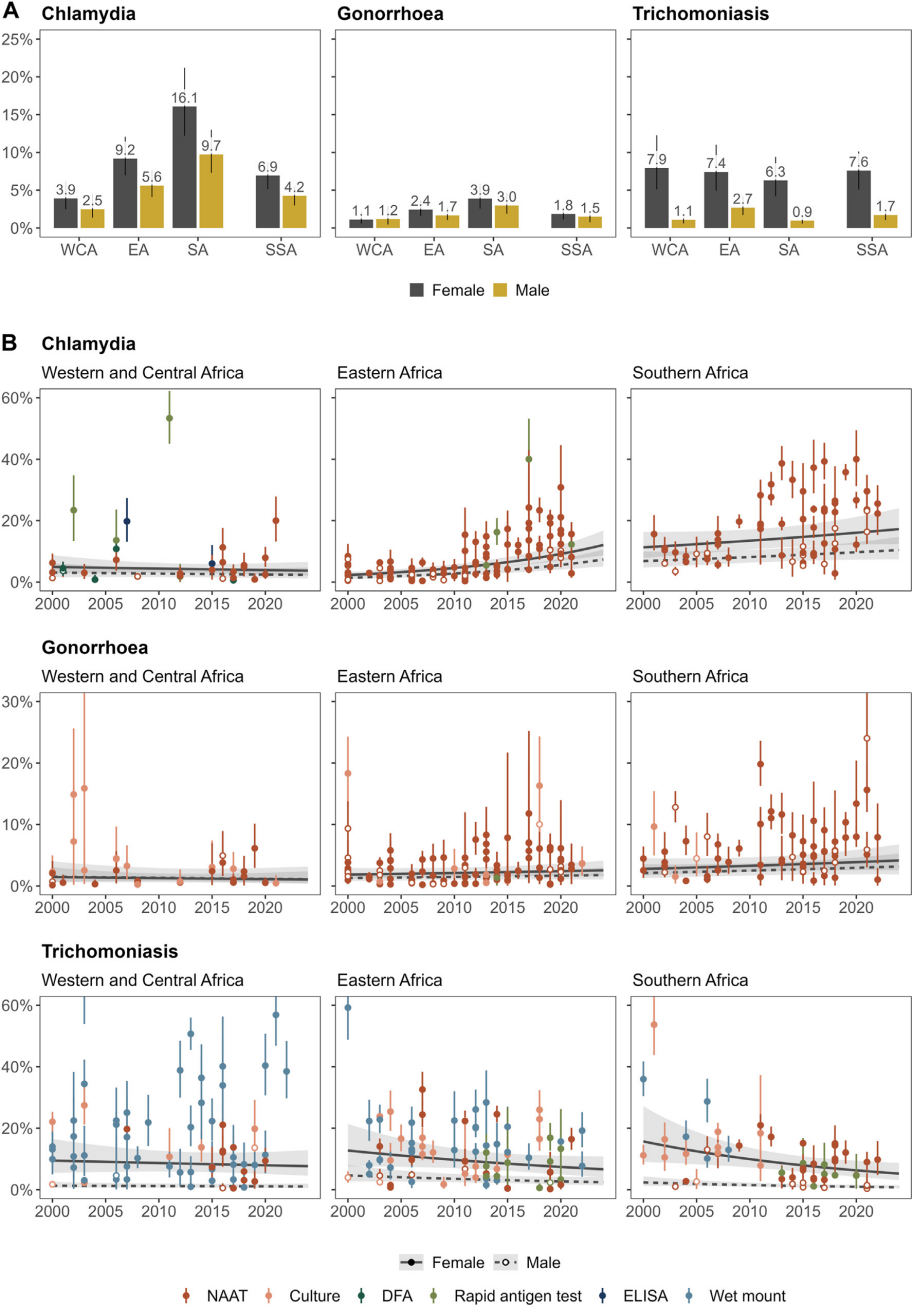
**Sexually transmitted infection prevalence per sub-region in sub-Saharan Africa.** Estimates of chlamydia, gonorrhoea, and trichomoniasis prevalence by sex and sub-region. Sub-regional estimates generated using log-binomial generalised linear mixed-effects models with study observations between 2000 and 2024. Sub-Saharan African estimates represent sex-matched population-weighted means. Panel A: Prevalence estimates in 2020. Bars and error lines depict mean prevalence estimates with 95% confidence intervals. Panel B: Prevalence estimates between 2000 and 2024. Lines and shaded areas depict mean prevalence estimates (solid line: females, dashed line: males) with 95% confidence intervals. Points and error lines represent study prevalence observations (solid point: female, open point: male) with 95% confidence intervals, colour coded according to diagnostic test category. Y-axes truncated. DFA: Direct fluorescent antibody, EA: Eastern Africa, NAAT: nucleic acid amplification test, SA: Southern Africa, SSA: Sub-Saharan Africa, WCA: Western and Central Africa.

In 2020, estimated prevalence of chlamydia in sub-Saharan Africa was 6.9% (95% CI: 5.2–8.7%, number of observations (n) = 169) among females and 4.2% (3.0–5.5%, n = 33) among males, gonorrhoea was 1.8% (1.1–2.5%, n = 171) and 1.5% (0.8–2.2%, n = 31), and trichomoniasis was 7.6% (5.1–10.2%, n = 188) and 1.7% (1.1–2.4%, n = 19), respectively (Fig. 2A, Supplementary Table S7). From 2010 to 2020, chlamydia prevalence increased by 34.5% (11.1–62.9%) in sub-Saharan Africa, while trends for gonorrhoea (3.3% increase [−20.2 to 33.9%]) and trichomoniasis (17.8% increase [−3.8 to 34.9%]) were not statistically significant (Table 2, Fig. 2B).

**Table 2:** Adjusted prevalence ratios for chlamydia, gonorrhoea, and trichomoniasis in sub-Saharan Africa, estimated via log-binomial generalised linear mixed-effects models.

Variable	Chlamydia aPR (95% CI)	Gonorrhoea aPR (95% CI)	Trichomoniasis aPR (95% CI)
Intercept	0.14 (0.11–0.18)	0.03 (0.02–0.05)	0.09 (0.07–0.12)
Sub-region			
Western and Central	0.31 (0.22–0.43)	0.36 (0.23–0.57)	0.94 (0.68–1.31)
Eastern	0.38 (0.35–0.41)	0.64 (0.56–0.74)	1.01 (0.85–1.21)
Southern	Ref	Ref	Ref
Sub-region:sex^a^			
Western and Central:Male	0.63 (0.43–0.93)	1.05 (0.54–2.04)	0.14 (0.09–0.20)
Eastern:Male	0.61 (0.53–0.70)	0.69 (0.56–0.85)	0.36 (0.30–0.44)
Southern:Male	0.61 (0.55–0.66)	0.76 (0.65–0.90)	0.15 (0.11–0.20)
Sub-region:year^b^			
Western and Central:Year	0.99 (0.95–1.03)	0.99 (0.94–1.04)	0.99 (0.96–1.02)
Eastern:Year	1.07 (1.05–1.09)	1.01 (0.99–1.04)	0.97 (0.94–1.01)
Southern:Year	1.02 (1.00–1.04)	1.02 (0.99–1.05)	0.96 (0.92–0.99)
Population			
ANC attendees	Ref	Ref	Ref
FP attendees	0.95 (0.63–1.45)	0.90 (0.50–1.64)	0.54 (0.32–0.90)
GYN attendees	1.03 (0.57–1.88)	0.87 (0.42–1.82)	1.34 (0.80–2.25)
PHC/OPD attendees	0.81 (0.57–1.14)	1.49 (0.92–2.39)	0.94 (0.61–1.43)
Students	1.06 (0.69–1.63)	1.14 (0.56–2.31)	1.23 (0.76–1.99)
Community members	0.97 (0.66–1.44)	1.10 (0.65–1.87)	0.96 (0.50–1.86)
HIV/STI prevention trial participants	0.89 (0.61–1.28)	1.20 (0.72–1.99)	1.00 (0.62–1.63)
Population-representative survey participants	0.90 (0.58–1.42)	1.19 (0.64–2.20)	1.32 (0.71–2.44)
Age group^c^			
All adults (12+ years)	Ref	Ref	Ref
Younger adults (12–25 years)	1.14 (0.85–1.52)	1.12 (0.75–1.68)	0.55 (0.35–0.88)
HIV status			
Non-stratified	Ref	Ref	Ref
HIV negative	1.42 (1.06–1.90)	1.06 (0.71–1.59)	0.79 (0.56–1.14)
Diagnostic test			
NAAT	Ref	Ref	Ref
Culture	–	1.60 (1.00–2.54)	1.36 (0.89–2.09)
DFA	0.70 (0.32–1.51)	–	–
ELISA	2.26 (0.86–5.98)	–	–
Rapid antigen test	2.94 (1.76–4.92)	0.52 (0.06–4.61)	1.03 (0.56–1.90)
Wet mount	–	–	1.61 (1.14–2.29)
Model variance			
*τ*^2^ fixed	0.51 (54.9%)	0.16 (16.3%)	0.45 (38.0%)
*τ*^2^ random	0.34 (36.4%)	0.60 (60.8%)	0.60 (50.9%)
*τ*^2^ distribution	0.08 (8.7%)	0.23 (22.9%)	0.13 (11.0%)
*τ*^2^ total	0.93 (100%)	0.98 (100%)	1.19 (100%)
Number groups			
Number studies	139	140	162
Number observations	202	202	207

Generalised linear mixed-effects models on the risk of chlamydia, gonorrhoea, and trichomoniasis in sub-Saharan Africa, using all study observations adjusted for diagnostic test performance.

aPR: adjusted prevalence ratio, 95% CI: 95% confidence interval, ANC: antenatal care, DFA: direct fluorescent antibody, FP: family planning clinic, GYN: gynaecology clinic, NAAT: nucleic acid amplification test, PHC/OPD: primary health care or outpatient department, Ref: Reference group, *τ*^2^: variance.

a Reference sex is female.

b Midpoint year between start and end of data collection period; centred at 2012.

c Age groups are not mutually exclusive due to limitations in study reporting.

Sub-regional variations in prevalence were similar for chlamydia and gonorrhoea (Table 2, Fig. 2A). Among females, relative to Southern Africa, the prevalence of chlamydia and gonorrhoea were lowest in Western and Central Africa (aPR_CT_: 0.31 [0.22–0.43], aPR_NG_: 0.36 [0.23–0.57]), followed by Eastern Africa (aPR_CT_: 0.38 [0.35–0.41], aPR_NG_: 0.64 [0.56–0.74]). Similarly for males, prevalence was lower in Western and Central (aPR_CT_: 0.38 [0.32–0.46], aPR_NG_: 0.58 [0.44–0.78]) and Eastern Africa (aPR_CT_: 0.32 [0.19–0.53], aPR_NG_: 0.50 [0.23–1.10]) compared to Southern Africa. For trichomoniasis, female prevalence was similar across sub-regions (WCA aPR_TV_: 0.94 [0.68–1.31], EA aPR_TV_: 1.01 [0.85–1.21]), while male prevalence was higher in Eastern (aPR_TV_: 2.45 [1.66–3.61]) than Southern Africa. Time trends for each infection varied across sub-regions. Chlamydia prevalence increased in Eastern (aPR_CT_ per year: 1.07 [1.05–1.09]) and Southern Africa (aPR_CT_: 1.02 [1.00–1.04]), while trichomoniasis prevalence decreased in Southern Africa (aPR_TV_: 0.96 [0.92–0.99]) only. Gonorrhoea prevalence was stable across all three sub-regions (Table 2, Fig. 2B).

Across study populations, there was no evidence that the prevalence of any infection differed compared to ANC attendees, except for lower trichomoniasis prevalence among family planning clinic attendees (aPR_TV_: 0.54 [0.32–0.90]). Studies diagnosing chlamydia with a rapid antigen test (aPR_CT_: 2.94 [1.76–4.92]), gonorrhoea with culture (aPR_NG_: 1.60 [1.00–2.54]), and trichomoniasis with wet mount (aPR_TV_: 1.61 [1.14–2.29]) had higher prevalence than those that used NAAT, after accounting for diagnostic test performance (Table 2). Study observations were heterogenous for all three infections, with observation-level random effect variance comprising 36.4%, 60.8%, and 50.9% of total variance for chlamydia, gonorrhoea, and trichomoniasis, respectively (Table 2).

### Male-to-female prevalence ratio estimates

**Fig. 3:**
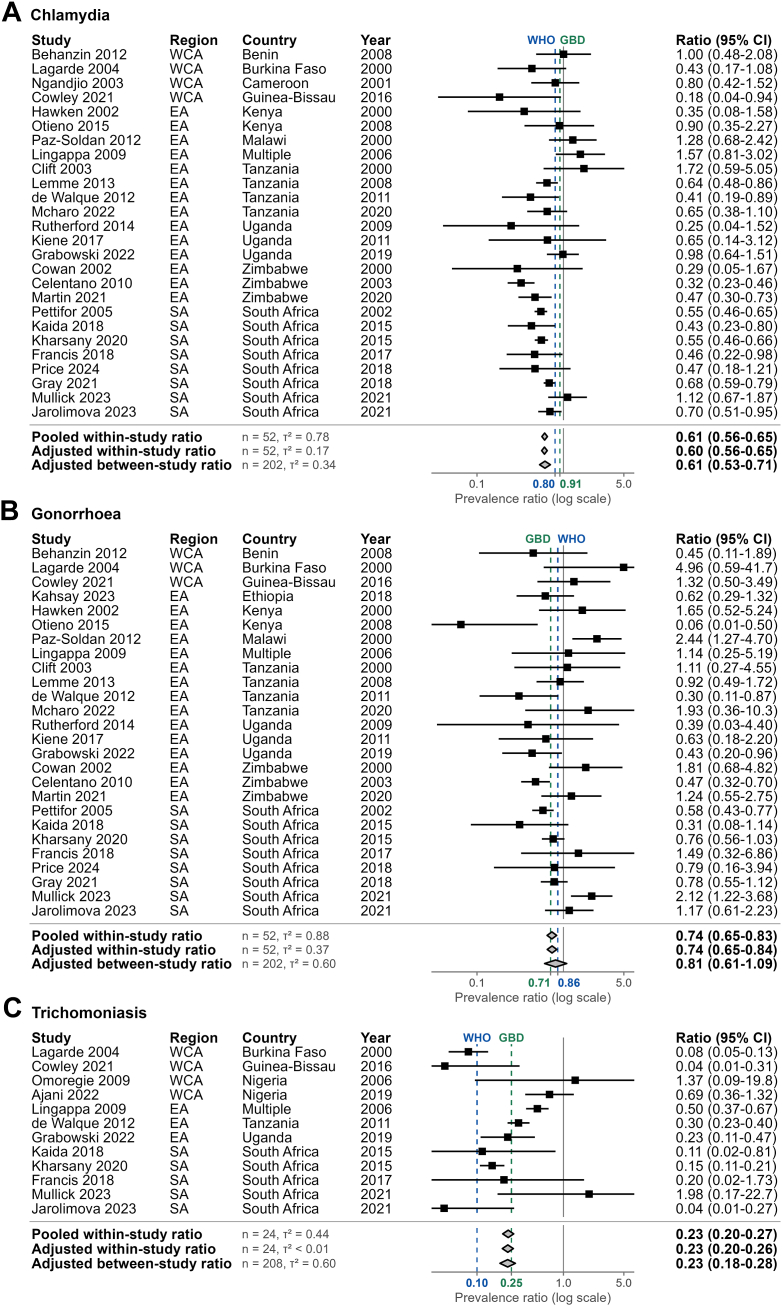
**Sexually transmitted infection male-to-female prevalence ratio estimates in sub-Saharan Africa.** Study observations and aggregate estimates of male-to-female prevalence ratios for (A) chlamydia, (B) gonorrhoea and (C) trichomoniasis. Ratios estimated using log-binomial generalised linear mixed-effects models per infection. Models for within-study ratios used study observations among both sexes and between-study ratios used all study observations. All models included sex as a fixed effect and study-level random intercepts. Pooled ratio models did not adjust for additional covariates. Adjusted ratio models included fixed effects for region, year, age-group, HIV-status, diagnostic test, and population group (between-study only). Points and error lines depict ratios and 95% confidence intervals for studies with observations among both sexes. Diamonds depict population-weighted mean ratios and 95% confidence intervals for sub-Saharan Africa, with adjusted ratios reported for 2020. Blue dotted lines and x-axis labels represent WHO global male-to-female prevalence ratios.^5^^,^^8^ Green dotted lines and x-axis labels represent the Global Burden of Disease study male-to-female prevalence ratios.^10^ X-axes are truncated. EA: Eastern Africa, SA: Southern Africa, WCA: Western and Central Africa, n: number of observations, τ^2^: variance for study random intercept.

In sub-Saharan Africa, male-to-female prevalence ratio estimates for each infection were similar when estimated using adjusted between-study ratios (all available data), adjusted within-study ratios (paired data), or unadjusted pooled within-study ratios (Fig. 3, Table 2, Supplementary Table S8). Male-to-female prevalence ratio estimates (adjusted between-study) for sub-Saharan Africa in 2020 were 0.61 (0.53–0.71) for chlamydia, 0.81 (0.61–1.09) for gonorrhoea, and 0.23 (0.18–0.28) for trichomoniasis (Fig. 3, Table 2). Between-study prevalence ratios were consistent across sub-regions for chlamydia (WCA:0.63 [0.43–0.93], EA:0.61 [0.53–0.70], SA:0.61 [0.55–0.66]), but varied for gonorrhoea (WCA:1.05 [0.54–2.04], EA:0.69 [0.56–0.85], SA:0.76 [0.65–0.90]) and trichomoniasis (WCA:0.14 [0.09–0.20], EA:0.36 [0.30–0.44], SA:0.15 [0.11–0.20]; Table 2).

### Sensitivity analyses and risk of bias

Prevalence estimates in 2020 were consistent for all three infections when derived using all available or NAAT-only observations, with or without accounting for diagnostic test performance (Table 2, Supplementary Fig. S2 and Tables S9–S11). Temporal trends for chlamydia and gonorrhoea also remained consistent across sub-regions. However, for trichomoniasis, all-observation models showed a prevalence decline in Southern Africa (aPR_TV_: 0.96 [0.92–0.99] with and without test performance), while NAAT-only models did not (with test performance: 0.96 [0.90–1.02]; without: 0.96 [0.92–1.01]; Table 2, Supplementary Fig. S3 and Tables S9–S11). Between-study and within-study male-to-female prevalence ratios were consistent for all three infections, whether derived using all available or NAAT-only observations. However, accounting for test performance led to higher ratios for gonorrhoea and lower ratios for chlamydia and trichomoniasis (Table 2, Supplementary Fig. S4 and Tables S9–S14).

The risk of selection bias was high, as most studies lacked probability-based sampling (N = 174/211, 82.5%) or sufficient participant characterisation (N = 133/211, 63.0%). The risk of measurement bias was low, with nearly all studies using consistent diagnostic methods in all participants (N = 198/211, 93.8%). Study precision, defined as sample size greater than 100, was generally adequate (N = 201/211, 95.3%, Supplementary Table S15). Trichomoniasis prevalence was higher in studies with insufficient reporting on participant characteristics (aPR_TV_: 1.52 [1.22–1.89]) compared to those with more complete reporting (Supplementary Table S16). Chlamydia and gonorrhoea prevalence were higher in studies with sample sizes less than 100 (aPR_CT_: 1.37 [1.06–1.76]; aPR_NG_: 1.93 [1.32–2.82]) than those with higher precision. There was no evidence that other critical appraisal criteria influenced prevalence estimates (Supplementary Table S16).

## Discussion

Our analysis estimated the prevalence of chlamydia, gonorrhoea and trichomoniasis among general populations in sub-Saharan Africa between 2000 and 2024. We estimated a higher burden of curable STIs among females (6.9% chlamydia, 1.8% gonorrhoea, and 7.6% trichomoniasis in 2020) than males (4.2%, 1.5%, and 1.7% respectively), which exceeded global prevalence estimates (3.2%, 0.7%, and 2.7% for both sexes in 2020, respectively).^9^ Geographic variation was substantial; prevalence of chlamydia and gonorrhoea was highest in Southern Africa, followed by Eastern Africa and then Western and Central Africa. Trichomoniasis prevalence was similar across sub-regions for females but was highest in Eastern Africa for males. The prevalence of chlamydia increased considerably in Eastern and Southern Africa over the study period, while gonorrhoea was stable, and trichomoniasis decreased in Southern Africa.

Our central estimates of STI prevalence in 2020 differed from those reported by WHO for the African region and GBD for sub-Saharan Africa.^9^^,^^10^ For females, our estimates were higher for chlamydia (6.9% vs. WHO:5.5%, GBD:4.5%), similar for gonorrhoea (1.8% vs. WHO:1.6%, GBD:1.7%), and lower for trichomoniasis (7.6% vs. WHO:12.0%, GBD:12.6%). For males, our estimates were higher for chlamydia (4.2% vs. WHO:4.0%, GBD:2.5%), gonorrhoea (1.5% vs. WHO:1.2%, GBD:1.2%), and trichomoniasis (1.7% vs. WHO:1.3%, GBD:1.5%).^9^^,^^10^ These differences reflect our broader study inclusion criteria, incorporation of more recent studies, and analysis of temporal trends to reflect sub-regional changes in prevalence. Compared to existing global male-to-female prevalence ratios, our sub-Saharan Africa ratio estimates were lower for chlamydia (WHO: 0.80 and GBD: 0.91), and intermediate for gonorrhoea (WHO: 0.86 and GBD: 0.71) and trichomoniasis (WHO: 0.10 and GBD: 0.25; Fig. 3).^5^^,^^8^^,^^10^ Future estimation exercises should therefore prioritise using region-specific ratios to capture local epidemiologic differences, although sparse male prevalence data can hinder ratio estimation.

The spatial distribution of chlamydia and gonorrhoea prevalence mirror that of HIV prevalence in sub-Saharan Africa, characterised by highest burden in Southern Africa, followed by Eastern Africa, and lowest in Western and Central Africa.^36^ This is likely due to shared behavioural risk factors and biological synergies that facilitate co-infection.^37^ Despite evidence suggesting that trichomoniasis increases HIV transmission and acquisition risk,^38^ prevalence variations were not observed across sub-regions. This could reflect limitations in study availability and diagnostics, missing covariates, or incomplete adjustment for covariates such as age.^39^ Greater integration of STI and HIV services remains important for their effective mitigation in high-burden areas.^1^

The increasing prevalence of chlamydia in Eastern and Southern Africa and decreasing prevalence of trichomoniasis in Southern Africa have not been documented previously. Previous STI burden assessments reported stable prevalence over time for chlamydia and gonorrhoea in Kenya and South Africa^40^^,^^41^ or did not estimate time trends for curable STIs in sub-Saharan Africa.^8^^,^^11^, ^12^, ^13^, ^14^ Our temporal trend estimates were highly uncertain, predominantly due to high heterogeneity across included studies. For chlamydia, even the lower bound of our estimates suggest an upward trend. For trichomoniasis, however, declines in prevalence warrant cautious interpretation due to the transition from wet mount microscopy to more sensitive NAAT in recent studies.

Increases in chlamydia prevalence are particularly concerning given the severe, long-term health consequences of untreated infection, including chronic pelvic pain, infertility, ectopic pregnancy, and other adverse pregnancy outcomes, along with an increased risk of HIV transmission.^1^ Co-infection with HIV and other STIs additionally compounds the risk of adverse maternal and neonatal outcomes,^42^ making prevention and control strategies particularly critical in high-burden settings.

Globally, there are major concerns about the impact of rising antimicrobial resistance on gonorrhoea incidence.^43^ We did not find evidence of increasing gonorrhoea prevalence in sub-Saharan Africa. Data on antimicrobial resistance are sparse in the African region, but available evidence suggests that current first-line and dual therapies continue to be effective, despite resistance to older antibiotics.^44^

Differences in prevalence by sex arise from an interplay of biological, behavioural, and structural factors. Men are more like to experience symptoms of STI infection than women, leading to higher rates of treatment-seeking.^45^^,^^46^ Men also have a substantially shorter duration of trichomoniasis infection than women; despite potentially comparable incidence, faster clearance in men results in particularly low prevalence and thus a low male-to-female prevalence ratio.^46^ Disparities in treatment coverage, due to healthcare access barriers, sociocultural norms, and stigma,^1^ may also influence sex-prevalence differences. These factors, which may have changed over time, drive sub-regional variations in both prevalence and sex-prevalence ratios, but data are limited to attribute effects.

Our analysis was primarily limited by sparse and heterogenous data. Our systematic search identified only 211 studies in 28 of 48 sub-Saharan African countries. There were particularly few studies in Central Africa (12 studies in 6 countries) and among men (37 studies in 15 countries). We were therefore unable to generate estimates specific to Central Africa or to determine whether temporal prevalence trends differed by sex. Our estimates for Southern Africa relied predominantly on studies conducted in South Africa (51 of 59 studies in the sub-region), which limited our ability to assess the representativeness of both these studies and our estimates for the broader sub-region.

Limitations related to study representativeness and reporting, and adjustments made in our analysis, may have influenced our estimates. Given the lack of population-representative studies, we used ANC attendees as the reference category for our prevalence estimates. At least 65% of studies focused exclusively on sexually active individuals, including antenatal, family planning, and gynaecology clinic attendees, and HIV/STI prevention trial participants. We considered these groups to be representative of the general population, which may result in overestimating STI prevalence compared to the total adult population. Variability in reporting across studies hindered our adjustment of estimates for HIV prevalence, granular and mutually exclusive age groups, or urban/rural location, which likely influenced sub-regional heterogeneities. Furthermore, our adjustments for diagnostic test performance may have overestimated prevalence, particularly for trichomoniasis, due to the poor sensitivity of widely used wet mount microscopy. Our reliance on previously published WHO performance data, without accounting for improvements in diagnostic accuracy over time, may have resulted in over-adjusting recent study observations and thus overestimating temporal trends.

Despite these limitations, our systematic review provides a comprehensive analysis of chlamydia, gonorrhoea, and trichomoniasis prevalence in sub-Saharan Africa. We have addressed gaps in previous prevalence estimates by examining temporal trends and sub-regional variation, while incorporating studies from a range of populations considered representative of the general population. Our multiple analytical approaches yielded similar male-to-female prevalence ratio estimates, and our extensive sensitivity analyses of diagnostic test performance adjustments have strengthened the robustness of our findings.

Our analysis underscores the urgent need to strengthen STI control in sub-Saharan Africa, particularly in areas with a high burden and rising chlamydia prevalence. However, more comprehensive surveillance through population-representative studies, sentinel surveys, and research in underrepresented countries and population groups is essential for designing targeted mitigation strategies.^2^ These data will enable more strategic allocation of resources and interventions at national and sub-national levels.

In conclusion, we estimated a high and geographically varied prevalence of three curable STIs in sub-Saharan Africa, with concerning increases in chlamydia prevalence over time. Male-to-female prevalence ratios for sub-Saharan Africa and its sub-regions differed from existing WHO and GBD global ratios, emphasising the need to account for epidemiologic variations in future STI burden estimates and control strategies. Strengthening aetiologic surveillance through regular sentinel surveys and population-representative prevalence studies, with greater focus on male populations and underrepresented geographies, is essential for progressing toward WHO targets and reducing the STI burden in sub-Saharan Africa.

## Contributors

JM, JR, JWI-E, and M-CB conceptualised the study. DMR, EM, JM, JWI-E, JK, LH, MKW, OE, OS, QH, RLA, and SK screened abstracts and full text articles for inclusion. JM, LH, RLA, and QH extracted data from included studies. JM, LH, RLA, QH, and JR verified the data. JM assessed diagnostic test validity, collated diagnostic test performance characteristics, and performed the data analysis. JM wrote the initial manuscript draft, which was revised with input from all authors. All authors approved the final manuscript.

## Data sharing statement

Data extracted from included studies and used for analysis are available as supplementary material. Code and data reproducing the analysis are available from https://github.com/juliamichalow/sti-prevalence-ratios.

## Declaration of interests

JWI-E declares grants from UNAIDS and Gates Foundation, consulting fees from BAO systems, and meeting travel support from UNAIDS, Gates Foundation, and International AIDS Society outside the submitted work. AC declares grants from UK National Institute of Health Research and the Academy of Medical Science outside the submitted work. The authors alone are responsible for the views expressed in this article and they do not necessarily represent the views, decisions or policies of the institutions with which they are affiliated.
