# Supplementary material for: Prevalence of chlamydia, gonorrhoea, and trichomoniasis among male and female general populations in sub-Saharan Africa from 2000 to 2024: a systematic review and meta-regression analysis
Source: eClinicalMedicine. 2025 Apr 24;83:103210. doi: 10.1016/j.eclinm.2025.103210 (PMC12235391; doi:10.1016/j.eclinm.2025.103210)
Supplement: Supplementary Figs. S1–S4 and Tables S1–S16 [file mmc1.pdf]

Prevalence of chlamydia, gonorrhoea, and trichomoniasis among male and female general populations in sub-Saharan Africa from 2000-2024: A systematic review and meta-regression analysis

Supplementary material

Julia Michalow[1*], Lauren Hall[1], Jane Rowley PhD[2], Rebecca Anderson[1], Quinton Hayre[3], R Matthew Chico PhD[4], Olanrewaju Edun[1], Jesse Knight PhD[1], Salome Kuchukhidze PhD[3], Evidence Majaya[5], Domonique Reed PhD[3], Oliver Stevens[1], Magdelene K Walters[1], Remco PH Peters PhD[2], Anne Cori PhD[1], Prof Marie-Claude Boily PhD[1], Jeffrey W. Imai-Eaton PhD[1,3]

[1] MRC Centre for Global Infectious Disease Analysis, School of Public Health, Imperial College London, London, United Kingdom

[2] Department of Global HIV, Hepatitis and Sexually Transmitted Infections Programmes, World Health Organization, Geneva, Switzerland

[3] Center for Communicable Disease Dynamics, Department of Epidemiology, Harvard T.H. Chan School of Public Health, Boston, MA, USA

[4] Department of Disease Control, Faculty of Infectious and Tropical Diseases, London School of Hygiene & Tropical Medicine, London, United Kingdom

[5] Centre for Infectious Disease Epidemiology and Research, School of Public Health and Family Medicine, University of Cape Town, Cape Town, South Africa

[*] Corresponding author: Julia Michalow (j.michalow21@imperial.ac.uk)

List of Tables

S1 Search strategy for systematic review . . . 4

S2 Classification of included countries by sub-region in sub-Saharan Africa . . . 6

S3 Variables extracted from included studies . . . 6

S4 Sensitivity and specificity of diagnostic tests . . . 8

S5 Critical appraisal criteria . . . 9

S6 Overview of studies included in the systematic review . . . 10

S7 Sexually transmitted infection prevalence in 2010 and 2020 . . . 22

S8 Adjusted prevalence ratios for chlamydia, gonorrhoea, and trichomoniasis in sub-Saharan Africa, estimated via log-binomial generalised linear mixed-effects models: *within-study analysis using observations adjusted for diagnostic test performance* . . . 23

S9 Adjusted prevalence ratios for chlamydia, gonorrhoea, and trichomoniasis in sub-Saharan Africa, estimated via log-binomial generalised linear mixed-effects models: *between-study sensitivity analysis using observations unadjusted for diagnostic test performance* . . . 24

S10 Adjusted prevalence ratios for chlamydia, gonorrhoea, and trichomoniasis in sub-Saharan Africa, estimated via log-binomial generalised linear mixed-effects models: *between-study sensitivity analysis using NAAT-diagnosed observations adjusted for test performance* . 25

S11 Adjusted prevalence ratios for chlamydia, gonorrhoea, and trichomoniasis in sub-Saharan Africa, estimated via log-binomial generalised linear mixed-effects models: *between-study sensitivity analysis using NAAT-diagnosed observations unadjusted for test performance* 26

S12 Adjusted prevalence ratios for chlamydia, gonorrhoea, and trichomoniasis in sub-Saharan Africa, estimated via log-binomial generalised linear mixed-effects models: *within-study sensitivity analysis using observations unadjusted for diagnostic test performance* . . . 27

S13 Adjusted prevalence ratios for chlamydia, gonorrhoea, and trichomoniasis in sub-Saharan Africa, estimated via log-binomial generalised linear mixed-effects models: *within-study sensitivity analysis using NAAT-diagnosed observations adjusted for test performance* . 28

S14 Adjusted prevalence ratios for chlamydia, gonorrhoea, and trichomoniasis in sub-Saharan Africa, estimated via log-binomial generalised linear mixed-effects models: *within-study sensitivity analysis using NAAT-diagnosed observations unadjusted for test performance* 29

S15 Summary of critical appraisal criteria across studies . . . 33

S16 Adjusted prevalence ratios for chlamydia, gonorrhoea, and trichomoniasis in sub-Saharan Africa, estimated via log-binomial generalised linear mixed-effects models: *assessment of critical appraisal criteria using observations adjusted for diagnostic test performance* 34

List of Figures

S1 Number of studies included per country in sub-Saharan Africa. 21
S2 Sexually transmitted infection prevalence in sub-Saharan Africa in 2020, with and without accounting for diagnostic test performance. 30
S3 Sexually transmitted infection prevalence among females in sub-Saharan Africa between 2000 and 2024, with and without accounting for diagnostic test performance. 31
S4 Sexually transmitted infection male-to-female prevalence ratio estimates in sub-Saharan Africa, with and without accounting for diagnostic test performance. 32

Table S1: Search strategy for systematic review

**EMBASE:** Search conducted 17 September 2024 with 3794 articles retrieved.
**STI domain:** exp gonorrhea/ or exp Neisseria gonorrhoeae/ or exp Chlamydia trachomatis/ or exp Chlamydia trachomatis infection/ or exp vaginal trichomoniasis/ or exp trichomoniasis/ or exp Trichomonas vaginalis/ or (gonorrhea* or gonorrhoea* or gonorrhoeae* or chlamydia* or trichomonas or trichomoniasis).ab,ti,kw.
**AND sub-Saharan Africa domain:** exp Africa south of the Sahara/ or exp africa, eastern/ or exp africa, western/ or exp africa, central/ or exp africa, southern/ or (Africa or Angola or Benin or Botswana or Motswana or Batswana or Burkina or Burundi or "Cabo Verde" or "Cape verde" or Cameroon or "Central African Republic" or "Republique centrafricaine" or Chad or Comoros or Congo or "Democratic Republic of Congo" or "Republique democratique du Congo" or DRC or "Cote d'Ivoire" or "Ivory Coast" or Djibouti or Guinea or Eritrea or Erythree or Ethiopia or Gabon or Gambia or Ghana or Guinea or "Equatorial Guinea" or "Guinee Equatoriale" or "Equatoguinean" or "Guinea-Bissau" or Kenya or Lesotho or Basotho or Liberia or Madagascar or Malawi or Mali or Mauritania or Mozambique or Namibia or Niger or Nigeria or Rwanda or Rouanda or Ruanda or "Sao Tome" or Senegal or Seychelles or "Sierra Leone" or Somalia or Somali or "South Africa" or "Afrique du Sud" or "South Sudan" or "Soudan de sud" or Sudan or Swaziland or eSwatini or Tanzania or Togo or Uganda or Ouganda or Zambia or Zimbabwe).mp.
**AND** Limit publication year = "January 1, 2000 - Current"
**PubMed:** Search conducted 17 September 2024 with 2663 articles retrieved.
**STI domain:** "Neisseria gonorrhoeae"[MeSH Terms] OR "Gonorrhea"[MeSH Terms] OR "Chlamydia Infections"[MeSH Terms] OR "Chlamydia"[MeSH Terms] OR "Chlamydia trachomatis"[MeSH Terms] OR "Trichomonas vaginalis"[MeSH Terms] OR "Trichomonas Infections"[MeSH Terms] OR "Trichomonas"[MeSH Terms] OR "Trichomonas Vaginitis"[MeSH Terms] or gonorrhea*[Title/Abstract] OR gonorrhoea*[Title/Abstract] OR gonorrhoeae*[Title/Abstract] OR chlamydia*[Title/Abstract] OR trichomonas[Title/Abstract] OR trichomoniasis[Title/Abstract]
**AND sub-Saharan Africa domain:** "Africa south of the Sahara"[MeSH Terms] OR "africa, eastern"[MeSH Terms] OR "africa, western"[MeSH Terms] OR "africa, central"[MeSH Terms] OR "africa, southern"[MeSH Terms] OR "Africa"[All fields] OR "Angola"[All fields] OR "Benin"[All fields] OR "Botswana"[All fields] OR "Motswana"[All fields] OR "Batswana"[All fields] OR "Burkina"[All fields] OR "Burundi"[All fields] OR "Cabo Verde"[All fields] OR "Cape verde"[All fields] OR "Cameroon"[All fields] OR "Central African Republic"[All fields] OR "Republique centrafricaine"[All fields] OR "Chad"[All fields] OR "Comoros"[All fields] OR "Congo"[All fields] OR "Democratic Republic of Congo"[All fields] OR "Republique democratique du Congo"[All fields] OR "DRC"[All fields] OR "Cote d'Ivoire"[All fields] OR "Ivory Coast"[All fields] OR "Djibouti"[All fields] OR "Guinea"[All fields] OR "Eritrea"[All fields] OR "Ethiopia"[All fields] OR "Gabon"[All fields] OR "Gambia"[All fields] OR "Ghana"[All fields] OR "Guinea"[All fields] OR "Equatorial Guinea"[All fields] OR "Equatoguinean"[All fields] OR "Guinea-Bissau"[All fields] OR "Kenya"[All fields] OR "Lesotho"[All fields] OR "Basotho"[All fields] OR "Liberia"[All fields] OR "Madagascar"[All fields] OR "Malawi"[All fields] OR "Mali"[All fields] OR "Mauritania"[All fields] OR "Mozambique"[All fields] OR "Namibia"[All fields] OR "Niger"[All fields] OR "Nigeria"[All fields] OR "Rwanda"[All fields] OR "Rouanda"[All fields] OR "Ruanda"[All fields] OR "Sao Tome"[All fields] OR "Senegal"[All fields] OR "Seychelles"[All fields] OR "Sierra Leone"[All fields] OR "Somalia"[All fields] OR "Somali"[All fields] OR "South Africa"[All fields] OR "Afrique du Sud"[All fields] OR "South Sudan"[All fields] OR "Sudan"[All fields] OR "Swaziland"[All fields] OR "eSwatini"[All fields] OR "Tanzania"[All fields] OR "Togo"[All fields] OR "Uganda"[All fields] OR "Ouganda"[All fields] OR "Zambia"[All fields] OR "Zimbabwe"[All fields]
**AND** Limit publication year = "January 1, 2000 - Current"

Global Health: Search conducted 17 September 2024 with 2364 articles retrieved.

STI domain: exp gonorrhoea/ or exp Neisseria gonorrhoeae/ or exp Chlamydia trachomatis/ or exp Chlamydia/ or exp trichomoniasis/ or exp Trichomonas vaginalis/ or (gonorrhea* or gonorrhoea* or gonorrhoeae* or chlamydia* or trichomonas or trichomoniasis).ab,ti,mp.

AND sub-Saharan Africa domain: exp "Africa South of Sahara"/ or exp East Africa/ or exp Africa/ or exp Central Africa/ or exp Southern Africa/ or exp West Africa/ or (Africa or Angola or Benin or Botswana or Motswana or Batswana or Burkina or Burundi or "Cabo Verde" or "Cape verde" or Cameroon or "Central African Republic" or "Republique centrafricaine" or Chad or Comoros or Congo or "Democratic Republic of Congo" or "Republique democratique du Congo" or DRC or "Cote d'Ivoire" or "Ivory Coast" or Djibouti or Guinea or Eritrea or Erythree or Ethiopia or Gabon or Gambia or Ghana or Guinea or "Equatorial Guinea" or "Guinee Equatoriale" or "Equatoguinean" or "Guinea-Bissau" or Kenya or Lesotho or Basotho or Liberia or Madagascar or Malawi or Mali or Mauritania or Mozambique or Namibia or Niger or Nigeria or Rwanda or Rouanda or Ruanda or "Sao Tome" or Senegal or Seychelles or "Sierra Leone" or Somalia or Somali or "South Africa" or "Afrique du Sud" or "South Sudan" or "Soudan de sud" or Sudan or Swaziland or eSwatini or Tanzania or Togo or Uganda or Ouganda or Zambia or Zimbabwe).mp.

AND Limit publication year = "January 1, 2000 - Current"

MEDLINE: Search conducted 17 September 2024 with 2231 articles retrieved.

STI domain: exp Neisseria gonorrhoeae/ or exp Gonorrhea/ or exp Chlamydia Infections/ or exp Chlamydia/ or exp Chlamydia trachomatis/ or exp Trichomonas vaginalis/ or exp Trichomonas Infections/ or exp Trichomonas/ or exp Trichomonas Vaginitis/ or (gonorrhea* or gonorrhoea* or gonorrhoeae* or chlamydia* or trichomonas* or trichomoniasis*).ab,ti,kw.

AND sub-Saharan Africa domain: exp Africa, Western/ or exp Africa, Central/ or exp "Africa South of the Sahara"/ or exp Africa/ or exp Africa, Eastern/ or exp Africa, Southern/ or (Africa or Angola or Benin or Botswana or Motswana or Batswana or Burkina or Burundi or "Cabo Verde" or "Cape verde" or Cameroon or "Central African Republic" or "Republique centrafricaine" or Chad or Comoros or Congo or "Democratic Republic of Congo" or "Republique democratique du Congo" or DRC or "Cote d'Ivoire" or "Ivory Coast" or Djibouti or Guinea or Eritrea or Erythree or Ethiopia or Gabon or Gambia or Ghana or Guinea or "Equatorial Guinea" or "Guinee Equatoriale" or "Equatoguinean" or "Guinea-Bissau" or Kenya or Lesotho or Basotho or Liberia or Madagascar or Malawi or Mali or Mauritania or Mozambique or Namibia or Niger or Nigeria or Rwanda or Rouanda or Ruanda or "Sao Tome" or Senegal or Seychelles or "Sierra Leone" or Somalia or Somali or "South Africa" or "Afrique du Sud" or "South Sudan" or "Soudan de sud" or Sudan or Swaziland or eSwatini or Tanzania or Togo or Uganda or Ouganda or Zambia or Zimbabwe).mp.

AND Limit publication year = "January 1, 2000 - Current"

African Index Medicus: Search conducted 17 September 2024 with 84 articles retrieved.

STI domain: mh:("Chlamydia Infections" OR "Chlamydia Infection" OR "Chlamydia trachomatis" OR "Chlamydia", OR "Gonorrhoea" OR "Neisseria gonorrhoeae" OR "Trichomonas" OR "Trichomoniasis" OR "Trichomonas vaginalis" OR "Trichomonas infections" OR "Trichomonas infection") or tw:(gonorrhea* or gonorrhoea* or gonorrhoeae* or chlamydia* or trichomonas or trichomoniasis)

AND Limit publication year = "January 1, 2000 - Current"

Table S2: Classification of included countries by sub-region in sub-Saharan Africa

Sub-region	Country
Central Africa	Angola, Cameroon, Central African Republic, Chad, Congo, Democratic Republic of Congo, Equatorial Guinea, Gabon, Sao Tome and Principe
Western Africa	Benin, Burkina Faso, Cabo Verde, Cote d'Ivoire, Gambia, Ghana, Guinea-Bissau, Guinea, Liberia, Mali, Mauritania, Niger, Nigeria, Senegal, Sierra Leone, Togo
Eastern Africa	Burundi, Comoros, Djibouti, Eritrea, Ethiopia, Kenya, Madagascar, Malawi, Mauritius, Mozambique, Rwanda, Seychelles, Somalia, South Sudan, Uganda, United Republic of Tanzania, Zambia, Zimbabwe
Southern Africa	Botswana, Eswatini, Lesotho, Namibia, South Africa

Countries classified according to UN M49 Standard.[1]

Table S3: Variables extracted from included studies

Category	Variable
Study characteristics	• Authors<br>• Publication title<br>• Publication year<br>• Dates of data collection<br>• Country of study<br>• Sub-national region or city of study<br>• Study name (as applicable)
Participant characteristics	• Study population category<br>• Study population age (mean, SD, median, IQR, range)<br>• Study population HIV status<br>• Study population HIV prevalence
Diagnostic methodology	• Diagnostic test per infection<br>• Diagnostic specimen per infection<br>• Number tested per infection<br>• Number positive per infection<br>• Prevalence of each infection

Text S1: Adjustment of study observations for diagnostic test performance

We estimated the true prevalence (p^{true}) of infection by adjusting observed prevalence (p^{observed}) for diagnostic test sensitivity and specificity, using the Rogan-Gladen equation (Equation 1). To avoid yielding negative values for p^{true} when p^{observed} fell below the false positive rate, we used a Bayesian statistical framework that incorporated the sample size (N) and positive test count (n) for observed data with a binomial probability distribution (Equation 2). We implemented a uniform prior probability for p^{true} (Equation 3).[2,3]

The model was fit using the Markov Chain Monte Carlo NUTS (No-U-Turn Sampler) algorithm with four chains of 2000 iterations each. Convergence was assessed qualitatively using trace plots and quantitatively using the Gelman-Rubin statistic (R-hat). Adjusted numerators (n^{adjusted}) and denominators (N^{adjusted}) were calculated for each observation using the posterior mean estimate ($\hat{p}^{\text{true}}$) and standard error (se^{true}), which were then used for pooling in mixed-effects models (Equations 4 and 5).

$$p^{\text{observed}} = p^{\text{true}} \cdot \text{sensitivity} + (1 - p^{\text{true}})(1 - \text{specificity}) \tag{1}$$

$$n \sim \text{binomial}(N, p^{\text{observed}}) \tag{2}$$

$$p^{\text{true}} \sim \text{uniform}(0, 1) \tag{3}$$

$$N^{\text{adjusted}} = \frac{\hat{p}^{\text{true}}(1 - \hat{p}^{\text{true}})}{(se^{\text{true}})^2} \tag{4}$$

$$n^{\text{adjusted}} = \hat{p}^{\text{true}} \cdot N^{\text{adjusted}} \tag{5}$$

Table S4: Sensitivity and specificity of diagnostic tests

STI	Test	Specimen	N	Sensitivity (%)	Specificity (%)	Source
**Female**						
CT	DFA	Genital fluid	2	82.0	98.5	WHO 1999[4]
	DFA	Urine	1	82.0	98.5	WHO 1999[4]
	ELISA	Genital fluid	2	65.0	100.0	WHO 2011[5]
	NAAT	Genital fluid	105	88.6	99.6	WHO 2011[5]
	NAAT	Urine	41	87.0	99.8	WHO 2011[5]
	NAAT	Genital fluid or urine	9	87.0	99.6	Combined estimate[5,i]
	Rapid antigen test	Genital fluid	8	56.0	99.0	Grillo-Ardila 2020,[6] Zhou 2021[7]
	DFA and NAAT	genital fluid	1	82.0	99.6	Combined estimate[4,5,ii]
NG	Culture	Genital fluid	23	75.7	100.0	WHO 2011[5]
	NAAT	Genital fluid	98	93.3	99.2	WHO 2011[5]
	NAAT	Urine	40	91.6	100.0	WHO 2011[5]
	NAAT	Genital fluid or urine	8	91.6	99.2	Combined estimate[5,i]
	Rapid antigen test	Genital fluid	1	70.0	96.0	Watchirs Smith 2013[8]
	Culture or NAAT	Genital fluid or urine	1	75.7	99.2	Combined estimate[5,i]
TV	Culture	Genital fluid	32	68.8	100.0	WHO 2011[5]
	Culture	Urine	1	68.8	100.0	Assumed equivalent to genital fluid test.
	NAAT	Genital fluid	47	95.0	98.0	WHO 2011[5]
	NAAT	Urine	7	66.9	98.3	WHO 2011[5]
	NAAT	Genital fluid or urine	1	66.9	98.0	Combined estimate[5,i]
	Rapid antigen test	Genital fluid	17	83.3	98.8	Gaydos 2017[9]
	Wet mount	Genital fluid	71	52.0	100.0	WHO 2011[5]
	Wet mount	Urine	2	52.0	100.0	Assumed equivalent to genital fluid test.
	Wet mount	Genital fluid and urine	4	52.0	100.0	Assumed equivalent to genital fluid test.
	Culture and wet mount	Genital fluid	5	52.0	100.0	Combined estimate[5,ii]
	Culture or NAAT	Genital fluid or urine	1	66.9	98.0	Combined estimate[5,i]
	Wet mount and NAAT	Genital fluid	1	52.0	100.0	Combined estimate[5,ii]
**Male**						
CT	NAAT	Genital fluid	1	87.5	99.2	WHO 2011[5]
	NAAT	Urine	32	87.8	99.3	WHO 2011[5]
	DFA and NAAT	Genital fluid	1	82.0	99.2	Combined estimate[4,5,ii]
NG	Culture	Genital fluid	1	87.6	100.0	WHO 2011[5]
	NAAT	Genital fluid	1	96.1	99.0	WHO 2011[5]
	NAAT	Urine	29	80.9	99.9	WHO 2011[5]
	Culture or NAAT	Urine	1	80.9	99.9	Combined estimate[5,i]
TV	Culture	Urine	3	87.6	100.0	WHO 2011[5]
	Culture	Genital fluid or urine	1	87.6	100.0	Combined estimate[5,i]
	NAAT	Urine	13	96.0	97.7	WHO 2011[5]
	Rapid antigen test	Genital fluid	1	68.5	97.4	Assumed equivalent to test for females.
	Wet mount	Urine	1	44.0	100.0	WHO 2011[5]
	Culture or NAAT	Urine	1	87.6	97.7	Combined estimate[5,i]

N: Number of observations. CT: *Chlamydia trachomatis*, NG: *Neisseria gonorrhoeae*, TV: *Trichomonas vaginalis*. DFA: Direct fluorescent antibody, ELISA: Enzyme-linked immunosorbent assay, NAAT: Nucleic acid amplification test.

Sensitivity and specificity values collated per approach in Michalow 2024.[10] Combined performance characteristics were estimated by: (i) using the lower sensitivity and lower specificity values when at least one of two diagnostic approaches identified a positive case, and (ii) using the lower sensitivity and higher specificity values when both diagnostic approaches needed to be positive.

Table S5: Critical appraisal criteria

Category	Criteria	Definition of lower risk	Definition of higher risk
Selection bias	Participant sampling	Probability-based sampling with a stated randomisation method.	Non-probability based sampling, such as consecutive or convenient enrolment, or recruitment method not reported.
Selection bias	Participant characterisation	Participants described in sufficient detail to assess representativeness, with complete reporting on HIV prevalence, age range, mean or median age, and study location.	Participants not described in sufficient detail, with incomplete reporting on one or more key characteristics.
Measurement bias	Diagnostic method consistency	Standardised mode of sample collection and a single diagnostic technique consistently applied to all participants.	Variation in sample collection methods or use of multiple diagnostic techniques within the same study.
Study precision	Sample size adequacy	Study tested ≥ 100 participants.	Study tested < 100 participants.

Criteria adapted from the Joanna Briggs Institute critical appraisal tool for prevalence studies.[11]

Table S6: Overview of studies included in the systematic review

Region	Reference	Year	Country	Population	Sex	Age	Age range	HIV status	Infections
WA	Lagarde 2004[12]	2000	Burkina Faso	Population-representative survey participants	F, M	Adult	13 to 49	Non-stratified	CT, NG, TV
WA	Lafort 2003[13]	2000	Côte d'Ivoire	FP attendees	F	Adult	18 to 53	Non-stratified	CT, NG, TV
WA	Aboyeji 2003[14]	2000	Nigeria	ANC attendees	F	Adult	19 to 43	Non-stratified	NG, TV
WA	Donbraye 2010[15]	2000	Nigeria	ANC attendees	F	Adult	NR	Non-stratified	TV
WA	Apea-Kubi 2004[16]	2001	Ghana	ANC attendees	F	Adult	16+	Non-stratified	CT, NG
WA	Apea-Kubi 2005[17]	2002	Ghana	ANC attendees	F	Adult	16+	Non-stratified	TV
WA	Adejuwon 2005[18]	2002*	Nigeria	FP attendees	F	Adult	NR	Non-stratified	NG, TV
WA	Obiajuru 2005[19]	2002	Nigeria	Community members	F	Adult	NR	Non-stratified	NG, TV
WA	Tukur 2006[20]	2002	Nigeria	FP attendees	F	Adult	NR	Non-stratified	CT
WA	Balaka 2005[21]	2002	Togo	ANC attendees	F	Adult	16 to 42	Non-stratified	TV
WA	Kirakoya-Samadoulougou 2008[22]	2003	Burkina Faso	ANC attendees	F	Adult	14 to 49	Non-stratified	TV
WA	Siemer 2008[23]	2003	Ghana	ANC attendees	F	Adult	NR	Non-stratified	CT
WA	Chigbu 2006[24]	2003*	Nigeria	ANC attendees, GYN attendees	F	Adult	15 to 65	Non-stratified	NG, TV
WA	Inabo 2006[25]	2003*	Nigeria	ANC attendees	F	Adult	18 to 47	Non-stratified	TV
WA	Jatau 2006[26]	2003*	Nigeria	ANC attendees	F	Adult	16+	Non-stratified	TV
WA	Sagay 2005[27]	2003	Nigeria	ANC attendees	F	Adult	NR	Non-stratified	TV
WA	Yirenya-Tawiah 2014[28]	2006	Ghana	Community members	F	Adult	15 to 49	Non-stratified	CT, NG
WA	Fayemiwo 2018[29]	2006	Nigeria	FP attendees	F	Adult	19 to 54	Non-stratified	CT, NG, TV
WA	Omoregie 2009[30]	2006*	Nigeria	PHC/OPD attendees	F, M	Adult	NR	HIV negative	TV
WA	Kengne 2010[31]	2007*	Côte d'Ivoire	ANC attendees	F	Adult	NR	Non-stratified	CT, NG, TV
WA	Chinyere 2012[32]	2007	Nigeria	ANC attendees	F	Adult	15 to 40	Non-stratified	TV
WA	Niemogha 2010[33]	2007*	Nigeria	FP attendees, GYN attendees, Students	F	Adult	NR	Non-stratified	TV

Continued...

Region	Reference	Year	Country	Population	Sex	Age	Age range	HIV status	Infections
WA	Behanzin 2012[34]	2008	Benin	Population-representative survey participants	F, M	Adult	15 to 49	Non-stratified	CT, NG
WA	Usanga 2011[35]	2008	Nigeria	ANC attendees	F	Adult	15 to 49	Non-stratified	NG, TV
WA	Sam-Wobo 2012[36]	2009*	Nigeria	ANC attendees	F	Adult	16 to 50	Non-stratified	TV
WA	Arinze 2014[37]	2011*	Nigeria	Students	F	Adult	15 to 30	Non-stratified	CT
WA	Bolaji 2013[38]	2011	Nigeria	ANC attendees	F	Adult	20 to 40	Non-stratified	TV
WA	Tchelougou 2013[39]	2011	Togo	ANC attendees	F	Adult	NR	Non-stratified	TV
WA	Volker 2017[40]	2012	Ghana	ANC attendees	F	Adult	14 to 48	Non-stratified	CT, NG
WA	Adesiji 2015[41]	2012*	Nigeria	FP attendees	F	Adult	20+	Non-stratified	CT
WA	Olowe 2014[42]	2012	Nigeria	ANC attendees	F	Adult	21 to 40	Non-stratified	TV
WA	Samuel 2015[43]	2012*	Nigeria	ANC attendees	F	Adult	21 to 50	Non-stratified	TV
WA	Etuketu 2015[44]	2013	Nigeria	ANC attendees	F	Adult	15 to 44	Non-stratified	TV
WA	Nnaemeka 2016[45]	2013*	Nigeria	Population-representative survey participants	F	Adult	22 to 42	Non-stratified	TV
WA	Olusegun-Joseph 2016[46]	2013*	Nigeria	PHC/OPD attendees, Students	F	Adult	16 to 55	Non-stratified	TV
WA	Akinbo 2017[47]	2014*	Nigeria	Students	F	Youth	13 to 17	Non-stratified	TV
WA	Oyeyemi 2016[48]	2014	Nigeria	ANC attendees	F	Adult	21+	Non-stratified	TV
WA	Wokem 2015[49]	2014	Nigeria	ANC attendees	F	Adult	11 to 60	Non-stratified	TV
WA	Sangare 2021[50]	2015	Burkina Faso	ANC attendees	F	Adult	15 to 49	Non-stratified	TV
WA	Konadu 2019[51]	2015	Ghana	ANC attendees	F	Adult	12 to 54	Non-stratified	TV
WA	Alexander 2018[52]	2015	Nigeria	ANC attendees	F	Adult	15 to 60	Non-stratified	TV
WA	Ebhodaghe 2017[53]	2015	Nigeria	ANC attendees	F	Adult	19 to 43	HIV negative	CT, NG, TV
WA	Asmah 2017[54]	2016	Ghana	ANC attendees	F	Adult	NR	Non-stratified	TV
WA	Squire 2019[55]	2016	Ghana	GYN attendees	F	Adult	16+	Non-stratified	TV
WA	Cowley 2021[56]	2016	Guinea-Bissau	Population-representative survey participants	F, M	Adult	16 to 49	Non-stratified	CT, NG, TV
WA	Ezeanya 2019[57]	2016*	Nigeria	Students	F	Adult	15 to 39	Non-stratified	CT, TV
WA	Ukatu 2019[58]	2016*	Nigeria	ANC attendees	F	Adult	18 to 45	Non-stratified	TV

Continued...

Region	Reference	Year	Country	Population	Sex	Age	Age range	HIV status	Infections
WA	Kashibu 2018[59]	2017	Nigeria	ANC attendees	F	Adult	15 to 39	Non-stratified	TV
WA	Odaranle 2020[60]	2017	Nigeria	FP attendees	F	Adult	20 to 45	Non-stratified	TV
WA	Isara 2021[61]	2017	Gambia	ANC attendees	F	Adult	15 to 44	Non-stratified	CT, NG, TV
WA	Jary 2021[62]	2018	Mali	PHC/OPD attendees	F	Adult	18+	Non-stratified	CT, NG, TV
WA	Rasheed 2021[63]	2018	Nigeria	ANC attendees	F	Adult	18+	Non-stratified	TV
WA	Ajani 2022[64]	2019	Nigeria	Students	F, M	Adult	15 to 30	Non-stratified	TV
WA	Auta 2020[65]	2019	Nigeria	ANC attendees	F	Adult	15+	Non-stratified	TV
WA	Maureen 2022[66]	2019*	Nigeria	ANC attendees	F	Adult	18+	Non-stratified	CT, NG, TV
WA	Lingani 2021[67]	2020	Burkina Faso	ANC attendees	F	Adult	16 to 45	Non-stratified	CT
WA	Agabi 2023[68]	2020*	Nigeria	GYN attendees	F	Adult	16 to 57	Non-stratified	TV
WA	Enwuru 2024[69]	2020	Nigeria	ANC attendees	F	Adult	15+	HIV negative	TV
WA	Butcher 2023[70]	2020	Gambia	Prevention trial participants	F	Adult	15 to 69	Non-stratified	CT, NG, TV
WA	Ngom 2023[71]	2021	Senegal	ANC attendees	F	Adult	16 to 46	Non-stratified	NG, TV
CA	Ngandjio 2003[72]	2001	Cameroon	Students	F, M	Youth	NR	Non-stratified	CT
CA	Kinoshita-Moleka 2008[73]	2004	Democratic Republic of the Congo	ANC attendees	F	Adult	15 to 45	Non-stratified	CT, NG
CA	Mbu 2008[74]	2006	Cameroon	ANC attendees	F	Adult	NR	Non-stratified	CT, NG, TV
CA	Alexandre 2015[75]	2012	Angola	PHC/OPD attendees	F	Adult	14 to 52	Non-stratified	CT, NG
CA	Vieira-Baptista 2017[76]	2015	Sao Tome and Principe	GYN attendees	F	Adult	21 to 60	Non-stratified	CT, NG, TV
CA	Compain 2019[77]	2017	Chad	Community members	F	Adult	20 to 65	Non-stratified	CT, NG, TV
CA	Nodjikouambaye 2019[78]	2017	Chad	GYN attendees	F	Adult	18+	Non-stratified	CT, NG, TV
CA	Gadoth 2019[79]	2017	Democratic Republic of the Congo	ANC attendees	F	Adult	18+	HIV negative	CT, NG, TV
CA	Mbah 2022[80]	2018	Cameroon	ANC attendees	F	Adult	15 to 46	Non-stratified	CT, NG, TV
CA	Payne 2020[81]	2018	Cameroon	PHC/OPD attendees	F	Adult	15 to 55	Non-stratified	TV

Continued...

Region	Reference	Year	Country	Population	Sex	Age	Age range	HIV status	Infections
CA	Ngombe Mouabata 2024[82]	2021	Congo	GYN attendees	F	Adult	21 to 71	Non-stratified	CT
CA	Eyong 2023[83]	2022	Cameroon	PHC/OPD attendees	F	Adult	17 to 53	Non-stratified	TV
EA	Hawken 2002[84]	2000	Kenya	Population-representative survey participants	F, M	Adult	15 to 49	Non-stratified	CT, NG
EA	Kaydos-Daniels 2003[85]	2000	Malawi	PHC/OPD attendees	M	Adult	18+	Non-stratified	TV
EA	Paz-Soldan 2012[86]	2000	Malawi	Population-representative survey participants	F, M	Adult	15-44	Non-stratified	CT, NG
EA	Menendez 2010[87]	2000	Mozambique	ANC attendees	F	Adult	14 to 61	Non-stratified	CT, NG, TV
EA	Clift 2003[88]	2000	United Republic of Tanzania	Community members	F, M	Adult	16 to 54	Non-stratified	CT, NG
EA	Cowan 2002[89]	2000	Zimbabwe	Community members	F, M	Youth	16 to 19	Non-stratified	CT, NG
EA	van de Wijgert 2009[90]	2002	Uganda, Zimbabwe	FP attendees	F	Adult	18 to 35	HIV negative	CT, NG, TV
EA	Munjoma 2010[91]	2002	Zimbabwe	ANC attendees	F	Adult	NR	Non-stratified	TV
EA	Bailey 2007[92]	2003	Kenya	Prevention trial participants	M	Youth	18 to 24	HIV negative	CT, NG, TV
EA	Ghebremichael 2009[93]	2003	United Republic of Tanzania	Community members	F	Adult	20 to 44	Non-stratified	CT, NG, TV
EA	Ghebremichael 2011[94]	2003	United Republic of Tanzania	Community members	M	Adult	20+	Non-stratified	CT, TV
EA	Mapingure 2010[95]	2003	United Republic of Tanzania, Zimbabwe	ANC attendees	F	Adult	14 to 43	Non-stratified	TV
EA	Msuya 2009[96]	2003	United Republic of Tanzania	ANC attendees	F	Adult	14 to 43	Non-stratified	NG, TV
EA	Celentano 2010[97]	2003	Zimbabwe	Community members	F, M	Adult	18 to 30	Non-stratified	CT, NG, TV
EA	Mensch 2008[98]	2004	Malawi	Population-representative survey participants	F	Youth	15 to 21	Non-stratified	CT, NG, TV
EA	Lujan 2008[99]	2004	Mozambique	ANC attendees	F	Adult	15 to 45	Non-stratified	CT, NG
EA	Kamali 2010[100]	2004	Uganda	Prevention trial participants	F	Adult	18 to 45	Non-stratified	CT, NG, TV

Continued...

Region	Reference	Year	Country	Population	Sex	Age	Age range	HIV status	Infections
EA	Tann 2006[101]	2004	Uganda	ANC attendees	F	Adult	15 to 40	Non-stratified	CT, NG, TV
EA	Ramjee 2008[102]	2004	Zambia	Prevention trial participants	F	Adult	18+	Non-stratified	CT, NG, TV
EA	Venkatesh 2011[103]	2004	Zimbabwe	Prevention trial participants	F	Adult	18 to 49	HIV negative	CT, NG, TV
EA	Gray 2009[104]	2005	Uganda	Community members	F	Adult	15 to 49	HIV negative	TV
EA	Lingappa 2009[105]	2006	Botswana, Kenya, Rwanda, South Africa, United Republic of Tanzania, Uganda, Zambia	Prevention trial participants	F, M	Adult	18+	HIV negative	CT, NG, TV
EA	Chersich 2009[106]	2006	Kenya	PHC/OPD attendees	F	Adult	16 to 45	Non-stratified	TV
EA	Guffey 2014[107]	2006	Malawi, Zambia, Zimbabwe	Prevention trial participants	F	Adult	18+	HIV negative	CT, NG, TV
EA	McCormack 2010[108]	2007	United Republic of Tanzania, Uganda, Zambia	Prevention trial participants	F	Adult	16+	HIV negative	CT, NG, TV
EA	Crucitti 2010[109]	2007*	Zambia	ANC attendees, Students	F	Adult	15 to 42	Non-stratified	TV
EA	Otieno 2015[110]	2008	Kenya	Community members	F, M	Adult	18 to 34	HIV negative	CT, NG
EA	Mocumbi 2017[111]	2008	Mozambique	Prevention trial participants	F	Adult	17 to 59	HIV negative	CT, NG, TV
EA	Lemme 2013[112]	2008	United Republic of Tanzania	Community members	F, M	Youth	15 to 30	Non-stratified	CT, NG
EA	Muvunyi 2011[113]	2009	Rwanda	Community members	F	Adult	21 to 45	Non-stratified	CT, NG
EA	Rutherford 2014[114]	2009	Uganda	Students	F, M	Youth	19 to 25	Non-stratified	CT, NG, TV
EA	Ademe 2013[115]	2010	Ethiopia	ANC attendees	F	Adult	15 to 49	Non-stratified	TV
EA	Chiduo 2012[116]	2010	United Republic of Tanzania	ANC attendees	F	Adult	18 to 44	Non-stratified	CT, NG, TV
EA	Downs 2012[117]	2010	United Republic of Tanzania	PHC/OPD attendees	F	Adult	18 to 50	Non-stratified	CT, NG
EA	Jespers 2014[118]	2011	Kenya	ANC attendees, FP attendees	F	Adult	18 to 35	Non-stratified	CT, NG, TV

Continued...

Region	Reference	Year	Country	Population	Sex	Age	Age range	HIV status	Infections
EA	de Walque 2012[119]	2011	United Republic of Tanzania	Prevention trial participants	F, M	Adult	18 to 30	Non-stratified	CT, NG, TV
EA	Lazenby 2014[120]	2011*	United Republic of Tanzania	GYN attendees	F	Adult	30 to 60	Non-stratified	CT, NG, TV
EA	Kiene 2017[121]	2011	Uganda	PHC/OPD attendees	F, M	Adult	18+	Non-stratified	CT, NG
EA	Ogilvie 2013[122]	2011	Uganda	Community members	F	Adult	26 to 69	Non-stratified	CT, NG
EA	Eshete 2013[123]	2012	Ethiopia	ANC attendees	F	Adult	15 to 36	Non-stratified	TV
EA	Kerubo 2016[124]	2012	Kenya	Students	F	Youth	14 to 17	Non-stratified	CT, NG, TV
EA	Kinuthia 2015[125]	2012	Kenya	ANC attendees	F	Adult	14+	HIV negative	CT, NG, TV
EA	Ravindran 2021[126]	2012	Kenya	ANC attendees	F	Adult	14+	HIV negative	CT, NG, TV
EA	Nkhoma 2017[127]	2012	Malawi	ANC attendees	F	Adult	15+	Non-stratified	TV
EA	Hokororo 2015[128]	2012	United Republic of Tanzania	ANC attendees	F	Youth	14 to 20	Non-stratified	CT, NG, TV
EA	Stephen 2017[129]	2012	Zimbabwe	ANC attendees	F	Adult	18+	Non-stratified	CT
EA	Mulu 2015[130]	2013	Ethiopia	ANC attendees	F	Adult	15 to 49	Non-stratified	NG, TV
EA	Maina 2016[131]	2013	Kenya	FP attendees	F	Adult	20 to 49	Non-stratified	CT, NG, TV
EA	Palanee-Phillips 2015[132]	2013	Malawi, Uganda, Zimbabwe	Prevention trial participants	F	Adult	18 to 45	HIV negative	CT, NG, TV
EA	Kestelyn 2018[133]	2013	Rwanda	Prevention trial participants	F	Adult	18 to 35	Non-stratified	CT, NG, TV
EA	Donders 2016[134]	2013*	Uganda	PHC/OPD attendees	F	Adult	NR	Non-stratified	CT, NG, TV
EA	Nakubulwa 2015[135]	2013	Uganda	ANC attendees	F	Adult	18+	Non-stratified	CT, TV
EA	Schonfeld 2018	2014	Ethiopia	ANC attendees	F	Adult	NR	Non-stratified	CT, NG, TV
EA	Kanyina 2017[136]	2014	Kenya	GYN attendees	F	Adult	15+	Non-stratified	TV
EA	Oliver 2018[137]	2014	Kenya	Community members	F	Adult	18 to 34	Non-stratified	CT, NG
EA	Franceschi 2016[138]	2014	Rwanda	Students	F	Youth	18 to 20	Non-stratified	CT
EA	Francis 2019[139]	2014	United Republic of Tanzania	Students	F	Youth	17 to 18	Non-stratified	CT, NG, TV
EA	Homsy 2019[140]	2014	Uganda	ANC attendees	F	Adult	18 to 49	HIV negative	TV
EA	Moses 2015[141]	2014	Uganda	Community members	F	Adult	30 to 65	Non-stratified	CT, NG

Continued...

Region	Reference	Year	Country	Population	Sex	Age	Age range	HIV status	Infections
EA	Chaponda 2021[142]	2014	Zambia	ANC attendees	F	Adult	NR	Non-stratified	CT, NG, TV
EA	Tadesse 2016[143]	2015	Ethiopia	GYN attendees	F	Adult	15 to 49	Non-stratified	NG
EA	Masese 2017[144]	2015	Kenya	Students	F	Youth	15 to 24	Non-stratified	CT, NG, TV
EA	Masha 2017[145]	2015	Kenya	ANC attendees	F	Adult	18 to 45	Non-stratified	CT, NG, TV
EA	Yuh 2020[146]	2015	Kenya	Students	F	Youth	16 to 20	HIV negative	CT, NG, TV
EA	Mukanyangezi 2018[147]	2015	Rwanda	GYN attendees	F	Adult	18+	HIV negative	TV
EA	Maufi 2016[148]	2015	United Republic of Tanzania	ANC attendees	F	Adult	17 to 46	Non-stratified	TV
EA	Yegorov 2018[149]	2015	Uganda	PHC/OPD attendees	F	Adult	18 to 45	HIV negative	CT, NG, TV
EA	Deese 2021[150]	2016	Kenya, Zambia	FP attendees	F	Adult	16 to 35	HIV negative	CT, NG
EA	Mgodi 2021[151]	2017	Kenya, Malawi, Mozambique, United Republic of Tanzania, Zimbabwe	Prevention trial participants	F	Adult	18 to 50	HIV negative	CT, NG
EA	Baussano 2021[152]	2017	Rwanda	Students	F	Youth	17 to 21	Non-stratified	CT
EA	Nsereko 2020[153]	2017	Rwanda	ANC attendees	F	Adult	18 to 49	Non-stratified	TV
EA	Masatu 2022[154]	2017	United Republic of Tanzania	FP attendees	F	Adult	18+	Non-stratified	CT
EA	Kahsay 2023[155]	2018	Ethiopia	PHC/OPD attendees	F, M	Adult	15+	Non-stratified	NG
EA	Madanitsa 2023[156]	2018	Kenya, Malawi, United Republic of Tanzania	ANC attendees	F	Adult	NR	HIV negative	CT, NG, TV
EA	Mehta 2023[157]	2018	Kenya	Students	F	Youth	14 to 22	Non-stratified	CT, NG, TV
EA	Celum 2022[158]	2019	Kenya	FP attendees	F	Youth	16 to 25	HIV negative	CT, NG
EA	Lokken 2022[159]	2019	Kenya	FP attendees	F	Adult	18 to 45	HIV negative	CT, NG, TV
EA	Juliana 2020[160]	2019	United Republic of Tanzania	ANC attendees	F	Adult	16 to 48	Non-stratified	CT, NG, TV
EA	Chitneni 2020[161]	2019	Uganda	PHC/OPD attendees	F	Adult	18 to 40	HIV negative	CT, NG, TV

Continued...

Region	Reference	Year	Country	Population	Sex	Age	Age range	HIV status	Infections
EA	Grabowski 2022[162]	2019	Uganda	Population-representative survey participants	F, M	Adult	18 to 49	Non-stratified	CT, NG, TV
EA	Husen 2023[163]	2020*	Ethiopia	ANC attendees	F	Adult	17 to 37	Non-stratified	TV
EA	Zenebe 2021[164]	2020	Ethiopia	ANC attendees	F	Adult	17 to 41	Non-stratified	CT, NG, TV
EA	Heffron 2021[165]	2020	Kenya	GYN attendees	F	Adult	15 to 30	HIV negative	CT, NG
EA	Mcharo 2022[166]	2020	United Republic of Tanzania	Students	F, M	Youth	18 to 24	Non-stratified	CT, NG
EA	Nair 2023[167]	2020	Uganda, Zimbabwe	Prevention trial participants	F	Youth	16 to 21	HIV negative	CT, NG, TV
EA	Martin 2021[168]	2020	Zimbabwe	Community members	F, M	Youth	16 to 24	Non-stratified	CT, NG
EA	Nyakambi 2022[169]	2021	Kenya	PHC/OPD attendees	F	Adult	18 to 49	Non-stratified	CT
EA	Oware 2023[170]	2021	Kenya	Prevention trial participants	F	Adult	18 to 30	HIV negative	CT, NG
EA	van der Veer 2024[171]	2021	Malawi	ANC attendees	F	Adult	NR	Non-stratified	CT, NG, TV
EA	Sineque 2024[172]	2021	Mozambique	PHC/OPD attendees	F	Adult	30 to 55	Non-stratified	CT, NG
EA	Mbuvi 2024[173]	2022	Kenya	PHC/OPD attendees	F	Adult	15 to 44	Non-stratified	NG, TV
EA	Senkoro 2024[174]	2022	United Republic of Tanzania	GYN attendees	F	Adult	18 to 45	Non-stratified	NG, TV
SA	Romoren 2007[175]	2000	Botswana	ANC attendees	F	Adult	15 to 43	Non-stratified	NG, TV
SA	Kleinschmidt 2007[176]	2000	South Africa	FP attendees	F	Adult	18 to 40	HIV negative	NG, TV
SA	Sturm 2004[177]	2001*	South Africa	ANC attendees	F	Adult	NR	Non-stratified	CT, NG, TV
SA	Paz-Bailey 2005[178]	2002	Botswana	FP attendees	F	Adult	NR	Non-stratified	NG, TV
SA	Pettifor 2005[179]	2002	South Africa	PHC/OPD attendees	F, M	Youth	15 to 24	Non-stratified	CT, NG
SA	van de Wijgert 2006[180]	2002	South Africa	PHC/OPD attendees	F	Adult	18 to 69	Non-stratified	CT, NG, TV
SA	Odendaal 2006[181]	2003	South Africa	ANC attendees	F	Adult	NR	Non-stratified	CT, NG
SA	Sobngwi-Tambekou 2009[182]	2003	South Africa	Prevention trial participants	M	Adult	18 to 24	Non-stratified	CT, NG, TV
SA	Ramjee 2008[102]	2004	South Africa	Prevention trial participants	F	Adult	18+	Non-stratified	CT, NG, TV
SA	Sebitloane 2011[183]	2004	South Africa	ANC attendees	F	Adult	18+	HIV negative	TV
SA	Venkatesh 2011[103]	2004	South Africa	Prevention trial participants	F	Adult	18 to 49	HIV negative	CT, NG, TV

Continued...

Region	Reference	Year	Country	Population	Sex	Age	Age range	HIV status	Infections
SA	Black 2008[184]	2005	South Africa	PHC/OPD attendees	M	Adult	NR	Non-stratified	CT, NG, TV
SA	Guffey 2014[107]	2006	South Africa	Prevention trial participants	F	Adult	18+	HIV negative	CT, NG, TV
SA	Lewis 2008[185]	2006	South Africa	PHC/OPD attendees	M	Adult	17 to 73	Non-stratified	CT, NG, TV
SA	De Jongh 2010[186]	2007*	South Africa	GYN attendees	F	Adult	13 to 41	Non-stratified	CT, NG, TV
SA	McCormack 2010[108]	2007	South Africa	Prevention trial participants	F	Adult	18+	HIV negative	CT, NG, TV
SA	Botswana Ministry of Health 2011[187]	2008	Botswana	FP attendees	F	Adult	NR	Non-stratified	CT, NG
SA	Thigpen 2012[188]	2008	Botswana	Prevention trial participants	F	Adult	18 to 39	HIV negative	TV
SA	Moodley 2015[189]	2009	South Africa	ANC attendees	F	Adult	18+	Non-stratified	CT, NG, TV
SA	Jespers 2014[118]	2011	South Africa	ANC attendees, FP attendees	F	Adult	18 to 35	Non-stratified	CT, NG, TV
SA	Kleppa 2015[190]	2011	South Africa	Students	F	Youth	15 to 31	Non-stratified	CT, NG, TV
SA	Peters 2014[191]	2011	South Africa	PHC/OPD attendees	F	Adult	18 to 49	Non-stratified	CT, NG
SA	Galappaththi-Arachchige 2016[192]	2012	South Africa	Students	F	Youth	16 to 20	Non-stratified	CT, NG, TV
SA	Shukla 2023[193]	2012	South Africa	Students	F	Youth	16 to 22	Non-stratified	CT, NG, TV
SA	Jewanraj 2021[194]	2013	South Africa	Prevention trial participants	F	Adult	20 to 44	HIV negative	CT, NG, TV
SA	Jongen 2021[195]	2013	South Africa	PHC/OPD attendees	F	Youth	16 to 24	HIV negative	CT, NG
SA	Palanee-Phillips 2015[132]	2013	South Africa	Prevention trial participants	F	Adult	18 to 45	HIV negative	CT, NG, TV
SA	Barnabas 2018[196]	2014	South Africa	PHC/OPD attendees	F	Youth	16 to 22	HIV negative	CT, NG, TV
SA	Le Roux 2017[197]	2014*	South Africa	PHC/OPD attendees	M	Adult	17 to 65	Non-stratified	CT, NG, TV
SA	Ginindza 2017[198]	2015	Eswatini	PHC/OPD attendees	F	Adult	15 to 49	Non-stratified	CT, NG, TV
SA	Abbai-Shaik 2016[199]	2015	South Africa	PHC/OPD attendees	M	Adult	18+	Non-stratified	CT
SA	Huyveneers 2023[200]	2015	South Africa	Prevention trial participants	F	Adult	18 to 45	HIV negative	CT, NG, TV
SA	Kaida 2018[201]	2015	South Africa	Community members	F, M	Youth	16 to 24	Non-stratified	CT, NG, TV
SA	Kharsany 2020[202]	2015	South Africa	Population-representative survey participants	F, M	Adult	15 to 49	Non-stratified	CT, NG, TV
SA	Wynn 2018[203]	2016	Botswana	ANC attendees	F	Adult	18+	Non-stratified	CT, NG, TV

Continued...

Region	Reference	Year	Country	Population	Sex	Age	Age range	HIV status	Infections
SA	Deese 2021[150]	2016	Eswatini, South Africa	FP attendees	F	Adult	16 to 35	HIV negative	CT, NG
SA	Gorgens 2020[204]	2016	Eswatini	Prevention trial participants	F	Youth	15 to 22	HIV negative	TV
SA	Gill 2020[205]	2016	South Africa	Prevention trial participants	F	Youth	15 to 19	HIV negative	CT, NG, TV
SA	Hoffman 2019[206]	2016	South Africa	PHC/OPD attendees	F	Adult	18 to 75	Non-stratified	CT, NG, TV
SA	Mgodi 2021[151]	2017	Botswana, South Africa	Prevention trial participants	F	Adult	18 to 50	HIV negative	CT, NG
SA	Delany-Moretlwe 2023[207]	2017	South Africa, Zimbabwe	Prevention trial participants	F	Youth	16 to 25	HIV negative	CT, NG, TV
SA	Francis 2018[208]	2017	South Africa	Population-representative survey participants	F, M	Youth	15 to 24	Non-stratified	CT, NG, TV
SA	Dessai 2020[209]	2018	South Africa	ANC attendees	F	Adult	18 to 43	Non-stratified	TV
SA	Govender 2023[210]	2018	South Africa	ANC attendees	F	Adult	15+	HIV negative	CT, NG, TV
SA	Gray 2021[211]	2018	South Africa	Prevention trial participants	F, M	Adult	18 to 35	HIV negative	CT, NG, TV
SA	Joseph Davey 2019[212]	2018	South Africa	ANC attendees	F	Adult	18+	Non-stratified	CT, NG, TV
SA	Naicker 2021[213]	2018	South Africa	ANC attendees	F	Adult	18+	Non-stratified	TV
SA	Price 2024[214]	2018	South Africa	Community members	F, M	Youth	12 to 19	HIV negative	CT, NG
SA	Taku 2021[215]	2018	South Africa	PHC/OPD attendees	F	Adult	30+	Non-stratified	CT, NG, TV
SA	Celum 2022[158]	2019	South Africa	FP attendees	F	Youth	16 to 25	HIV negative	CT, NG
SA	Chetty 2020[216]	2019	South Africa	ANC attendees	F	Adult	18+	Non-stratified	TV
SA	Oree 2021[217]	2019	South Africa	ANC attendees	F	Adult	20 to 40	Non-stratified	NG
SA	de Voux 2023[218]	2020	South Africa	ANC attendees	F	Adult	16+	HIV negative	CT, NG, TV
SA	Mabaso 2022[219]	2020	South Africa	ANC attendees	F	Adult	18+	Non-stratified	CT
SA	Nair 2023[167]	2020	South Africa	Prevention trial participants	F	Youth	16 to 21	HIV negative	CT, NG, TV
SA	Jarolimova 2023[220]	2021	South Africa	Population-representative survey participants	F, M	Youth	16 to 29	Non-stratified	CT, NG, TV
SA	Mullick 2023[221]	2021	South Africa	PHC/OPD attendees	F, M	Youth	15 to 24	HIV negative	CT, NG, TV
SA	Mussa 2023[222]	2022	Botswana	ANC attendees	F	Adult	15+	Non-stratified	CT, NG
SA	de Voux 2024[223]	2022	South Africa	ANC attendees	F	Adult	18+	Non-stratified	CT, NG, TV

Continued...

Region	Reference	Year	Country	Population	Sex	Age	Age range	HIV status	Infections
Multiple	Delany-Moretlwe 2022[224]	2019	Botswana, Eswatini, Kenya, Malawi, South Africa, Uganda, Zimbabwe	Prevention trial participants	F	Adult	18 to 45	HIV negative	CT, NG, TV

Region — CA: Central Africa, EA: Eastern Africa, SA: Southern Africa, WA: Western Africa.
Year — Midpoint year between start and end of data collection.*For studies without data collection dates reported, year was estimated by subtracting the median publication lag from the publication year (three years, based on difference among studies with dates reported).
Population — ANC: antenatal care, FP: family planning clinic, GYN: gynaecology clinic, PHC/OPD: primary healthcare or outpatient department.
Sex — F: Female, M: Male.
Age — Youth: 12–25 years, Adult: 12+ years.
Infections — CT: *Chlamydia trachomatis*, NG: *Neisseria gonorrhoeae*, TV: *Trichomonas vaginalis*.

The full study database is included as a separate supplementary file.

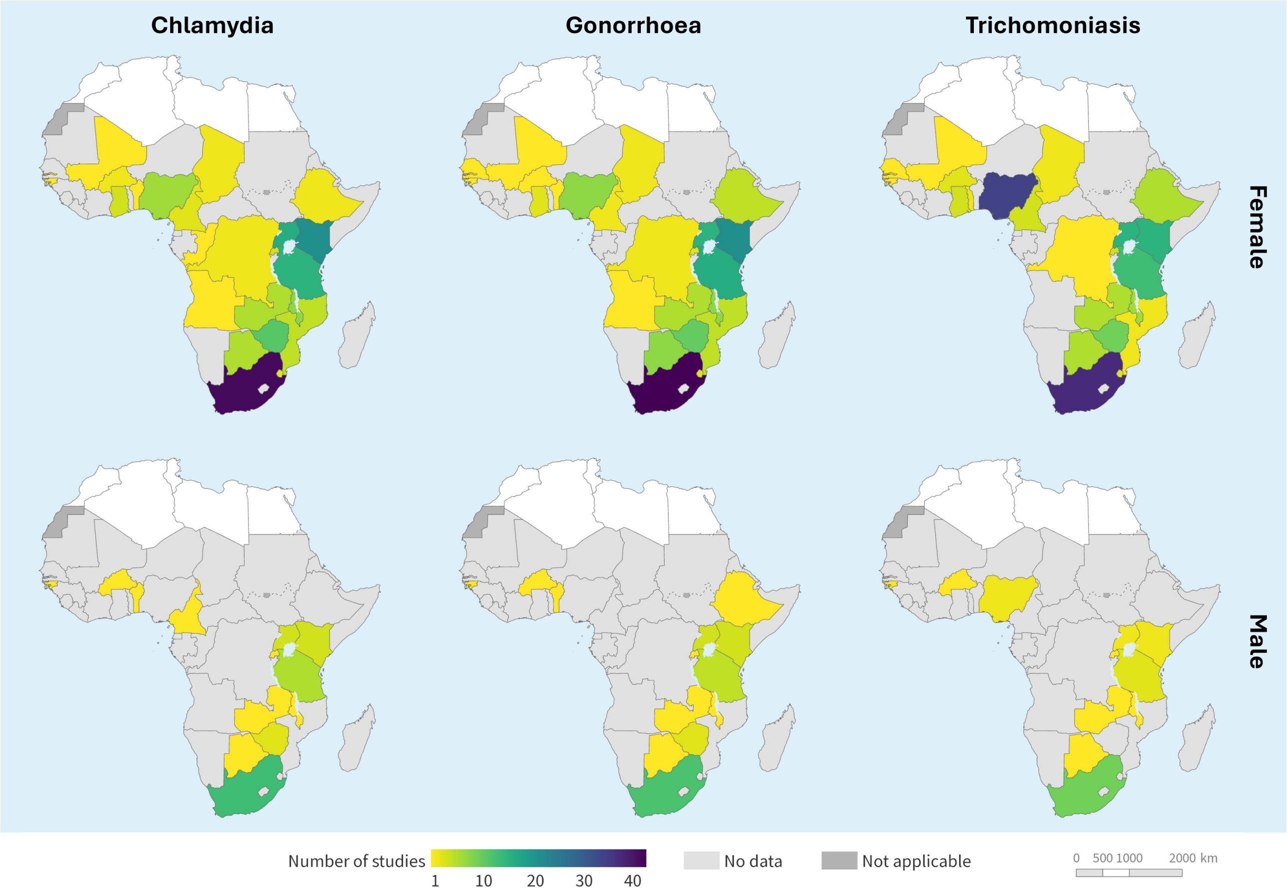

© World Health Organization 2025

Figure S1: Number of studies included per country in sub-Saharan Africa.

Number of studies assessing the prevalence of chlamydia, gonorrhoea, and trichomoniasis among females and males in sub-Saharan Africa. Grey shading represents countries with no identified studies. White shading represents countries outside of sub-Saharan Africa, according to the UN M49 Standard.[1] Total included studies were 139 for chlamydia, 140 for gonorrhoea, and 162 for trichomoniasis. Source for base map data is Natural Earth.[225]

The designations employed and the presentation of the material in this publication do not imply the expression of any opinion whatsoever on the part of WHO concerning the legal status of any country, territory, city or area or of its authorities, or concerning the delimitation of its frontiers or boundaries. Dotted and dashed lines on maps represent approximate border lines for which there may not yet be full agreement.

Table S7: Sexually transmitted infection prevalence in 2010 and 2020

Region	Female, % (95% CI)		Male, % (95% CI)	
	2010	2020	2010	2020
**Chlamydia**				
Western and Central Africa	4.4 (3.1-6.2)	3.9 (2.5-6.2)	2.8 (1.7-4.6)	2.5 (1.4-4.5)
Eastern Africa	4.6 (3.7-5.8)	9.2 (7.0-12.1)	2.8 (2.1-3.7)	5.6 (4.1-7.6)
Southern Africa	13.5 (10.6-17.1)	16.1 (12.2-21.2)	8.2 (6.3-10.5)	9.7 (7.3-13.0)
**Sub-Saharan Africa**	**5.1 (3.9-6.3)**	**6.9 (5.2-8.7)**	**3.2 (2.2-4.1)**	**4.2 (3.0-5.5)**
**Gonorrhoea**				
Western and Central Africa	1.3 (0.8-2.0)	1.1 (0.6-2.2)	1.3 (0.6-2.9)	1.2 (0.5-2.9)
Eastern Africa	2.1 (1.5-3.0)	2.4 (1.6-3.6)	1.5 (1.0-2.2)	1.7 (1.1-2.7)
Southern Africa	3.3 (2.3-4.7)	3.9 (2.6-5.9)	2.5 (1.7-3.7)	3.0 (1.9-4.6)
**Sub-Saharan Africa**	**1.8 (1.2-2.3)**	**1.8 (1.1-2.5)**	**1.5 (0.8-2.2)**	**1.5 (0.8-2.2)**
**Trichomoniasis**				
Western and Central Africa	8.6 (5.8-12.6)	7.9 (5.1-12.4)	1.2 (0.7-2.0)	1.1 (0.6-1.9)
Eastern Africa	9.7 (7.0-13.4)	7.4 (5.0-11.0)	3.5 (2.4-5.1)	2.7 (1.7-4.2)
Southern Africa	9.9 (7.2-13.6)	6.3 (4.2-9.4)	1.5 (1.0-2.3)	0.9 (0.6-1.6)
**Sub-Saharan Africa**	**9.1 (6.2-12.0)**	**7.6 (5.1-10.2)**	**2.1 (1.4-2.9)**	**1.7 (1.1-2.4)**

Estimates of chlamydia, gonorrhoea, and trichomoniasis prevalence by sex and sub-region in 2010 and 2020. Sub-regional estimates generated using log-binomial generalised linear mixed-effects models for each infection. Sub-Saharan African estimates represent sex-matched population-weighted means.

Table S8: Adjusted prevalence ratios for chlamydia, gonorrhoea, and trichomoniasis in sub-Saharan Africa, estimated via log-binomial generalised linear mixed-effects models: *within-study analysis using observations adjusted for diagnostic test performance*

Variable	Chlamydia aPR (95% CI)	Gonorrhoea aPR (95% CI)	Trichomoniasis aPR (95% CI)
**Intercept**	0.13 (0.08-0.21)	0.04 (0.02-0.07)	0.21 (0.17-0.26)
**Sub-region**			
Western and Central	0.28 (0.13-0.61)	0.54 (0.17-1.66)	0.46 (0.28-0.74)
Eastern	0.43 (0.27-0.68)	0.52 (0.26-1.04)	1.20 (0.86-1.67)
Southern	Ref	Ref	Ref
**Sub-region:year***			
Western and Central:Year	1.04 (0.96-1.12)	1.18 (1.04-1.33)	1.02 (1.00-1.04)
Eastern:Year	1.07 (1.03-1.10)	1.00 (0.95-1.05)	1.06 (0.82-1.39)
Southern:Year	1.05 (1.00-1.11)	1.05 (0.97-1.14)	0.89 (0.84-0.95)
**Sex**			
Female	Ref	Ref	Ref
Male	0.61 (0.56-0.65)	0.73 (0.65-0.83)	0.23 (0.20-0.27)
**Age group**			
Adult	Ref	Ref	Ref
Youth	1.17 (0.76-1.80)	1.25 (0.66-2.34)	0.36 (0.22-0.57)
**HIV status**			
Non-stratified	Ref	Ref	Ref
HIV negative	1.08 (0.67-1.73)	1.05 (0.52-2.14)	0.73 (0.19-2.70)
**Diagnostic test**			
NAAT	Ref	Ref	Ref
Culture	-	7.87 (1.92-32.19)	2.48 (1.50-4.10)
DFA	1.76 (0.52-5.94)	-	-
Rapid antigen test	-	-	0.40 (0.05-3.40)
Wet mount	-	-	0.88 (0.13-6.02)
**Model variance**			
τ^2 fixed	0.68	0.59	1.16
τ^2 random	0.17	0.37	<0.01
**Number groups**			
Number studies	26	26	12
Number observations	52	52	24

*Midpoint year between start and end of data collection period; centred at 2012. aPR: adjusted prevalence ratio, 95% CI: 95% confidence interval, DFA: direct fluorescent antibody, NAAT: nucleic acid amplification test, τ^2: variance.

Table S9: Adjusted prevalence ratios for chlamydia, gonorrhoea, and trichomoniasis in sub-Saharan Africa, estimated via log-binomial generalised linear mixed-effects models: *between-study sensitivity analysis using observations unadjusted for diagnostic test performance*

Variable	Chlamydia aPR (95% CI)	Gonorrhoea aPR (95% CI)	Trichomoniasis aPR (95% CI)
**Intercept**	0.13 (0.10-0.16)	0.03 (0.02-0.05)	0.10 (0.07-0.13)
**Sub-region**			
Western and Central	0.29 (0.21-0.40)	0.32 (0.20-0.52)	0.89 (0.64-1.23)
Eastern	0.36 (0.34-0.40)	0.63 (0.56-0.72)	0.99 (0.85-1.17)
Southern	Ref	Ref	Ref
**Sub-region:year***			
Western and Central:Year	0.99 (0.95-1.02)	0.99 (0.93-1.04)	0.99 (0.96-1.02)
Eastern:Year	1.07 (1.05-1.10)	1.01 (0.99-1.04)	0.97 (0.94-1.01)
Southern:Year	1.01 (0.99-1.03)	1.01 (0.98-1.04)	0.96 (0.92-0.99)
**Sub-region:sex†**			
Western and Central:Male	0.79 (0.57-1.08)	0.86 (0.45-1.66)	0.15 (0.10-0.23)
Eastern:Male	0.73 (0.65-0.82)	0.58 (0.48-0.70)	0.53 (0.46-0.62)
Southern:Male	0.64 (0.59-0.69)	0.60 (0.51-0.69)	0.26 (0.22-0.30)
**Population**			
ANC attendees	Ref	Ref	Ref
FP attendees	0.97 (0.64-1.48)	0.87 (0.46-1.66)	0.50 (0.29-0.85)
GYN attendees	0.97 (0.53-1.76)	0.69 (0.31-1.56)	1.47 (0.89-2.43)
PHC/OPD attendees	0.77 (0.55-1.09)	1.43 (0.86-2.35)	0.85 (0.56-1.29)
Students	1.05 (0.69-1.62)	1.21 (0.58-2.56)	1.12 (0.70-1.79)
Community members	0.99 (0.67-1.46)	1.14 (0.65-1.99)	0.88 (0.46-1.68)
Prevention trial participants	0.87 (0.60-1.26)	1.10 (0.64-1.89)	0.97 (0.60-1.55)
Population-representative survey participants	0.92 (0.59-1.44)	1.25 (0.65-2.40)	1.29 (0.71-2.35)
**Age group**			
Adult	Ref	Ref	Ref
Youth	1.12 (0.84-1.49)	1.01 (0.66-1.54)	0.63 (0.40-0.99)
**HIV status**			
Non-stratified	Ref	Ref	Ref
HIV negative	1.45 (1.09-1.94)	1.21 (0.79-1.85)	0.82 (0.57-1.17)
**Diagnostic test**			
NAAT	Ref	Ref	Ref
Culture	-	1.04 (0.63-1.71)	0.84 (0.55-1.28)
DFA	0.78 (0.37-1.64)	-	-
ELISA	1.55 (0.58-4.12)	-	-
Rapid antigen test	1.92 (1.16-3.18)	1.95 (0.35-10.89)	0.87 (0.48-1.58)
Wet mount	-	-	0.74 (0.53-1.04)
**Model variance**			
τ^2 fixed	0.47	0.22	0.22
τ^2 random	0.34	0.69	0.59
**Number groups**			
Number studies	139	140	162
Number observations	202	202	207

*Midpoint year between start and end of data collection period; centred at 2012. †Reference sex is female. aPR: adjusted prevalence ratio, 95% CI: 95% confidence interval, DFA: direct fluorescent antibody, ELISA: enzyme-linked immunosorbent assay, NAAT: nucleic acid amplification test, τ^2: variance.

Table S10: Adjusted prevalence ratios for chlamydia, gonorrhoea, and trichomoniasis in sub-Saharan Africa, estimated via log-binomial generalised linear mixed-effects models: *between-study sensitivity analysis using NAAT-diagnosed observations adjusted for test performance*

**Variable**	**Chlamydia aPR (95% CI)**	**Gonorrhoea aPR (95% CI)**	**Trichomoniasis aPR (95% CI)**
**Intercept**	0.15 (0.12-0.18)	0.04 (0.03-0.06)	0.14 (0.09-0.23)
**Sub-region**			
Western and Central	0.29 (0.21-0.40)	0.28 (0.17-0.47)	0.99 (0.40-2.46)
Eastern	0.38 (0.35-0.41)	0.64 (0.56-0.73)	0.58 (0.36-0.93)
Southern	Ref	Ref	Ref
**Sub-region:year***			
Western and Central:Year	1.01 (0.97-1.05)	1.06 (0.99-1.13)	0.88 (0.75-1.03)
Eastern:Year	1.07 (1.05-1.09)	1.02 (0.99-1.04)	0.95 (0.88-1.02)
Southern:Year	1.02 (1.00-1.04)	1.02 (0.99-1.05)	0.96 (0.90-1.02)
**Sub-region:sex†**			
Western and Central:Male	0.55 (0.33-0.91)	1.13 (0.57-2.21)	0.04 (0.01-0.33)
Eastern:Male	0.61 (0.53-0.70)	0.70 (0.56-0.86)	0.39 (0.31-0.47)
Southern:Male	0.61 (0.56-0.67)	0.80 (0.69-0.94)	0.15 (0.11-0.20)
**Population**			
ANC attendees	Ref	Ref	Ref
FP attendees	0.94 (0.61-1.45)	0.93 (0.50-1.73)	0.06 (0.00-0.82)
GYN attendees	0.98 (0.56-1.71)	0.65 (0.27-1.56)	0.69 (0.28-1.70)
PHC/OPD attendees	0.79 (0.57-1.11)	1.25 (0.77-2.03)	0.97 (0.48-1.93)
Students	0.84 (0.54-1.30)	0.92 (0.48-1.76)	2.24 (1.05-4.77)
Community members	0.93 (0.64-1.35)	0.82 (0.48-1.39)	0.68 (0.23-2.02)
Prevention trial participants	0.84 (0.59-1.19)	1.07 (0.65-1.73)	0.95 (0.39-2.31)
Population-representative survey participants	0.91 (0.60-1.40)	1.13 (0.63-2.02)	0.79 (0.31-1.99)
**Age group**			
Adult	Ref	Ref	Ref
Youth	1.22 (0.93-1.62)	1.24 (0.85-1.79)	0.33 (0.16-0.69)
**HIV status**			
Non-stratified	Ref	Ref	Ref
HIV negative	1.42 (1.07-1.88)	0.96 (0.65-1.42)	0.62 (0.32-1.22)
**Model variance**			
τ^2 fixed	0.53	0.24	1.07
τ^2 random	0.29	0.48	0.65
**Number groups**			
Number studies	127	115	56
Number observations	188	176	68

*Midpoint year between start and end of data collection period; centred at 2012. †Reference sex is female. aPR: adjusted prevalence ratio, 95% CI: 95% confidence interval, NAAT: nucleic acid amplification test, τ^2: variance.

Table S11: Adjusted prevalence ratios for chlamydia, gonorrhoea, and trichomoniasis in sub-Saharan Africa, estimated via log-binomial generalised linear mixed-effects models: *between-study sensitivity analysis using NAAT-diagnosed observations unadjusted for test performance*

**Variable**	**Chlamydia aPR (95% CI)**	**Gonorrhoea aPR (95% CI)**	**Trichomoniasis aPR (95% CI)**
**Intercept**	0.13 (0.11-0.16)	0.04 (0.03-0.06)	0.16 (0.11-0.24)
**Sub-region**			
Western and Central	0.27 (0.20-0.38)	0.28 (0.17-0.47)	0.98 (0.45-2.14)
Eastern	0.37 (0.34-0.40)	0.63 (0.56-0.71)	0.64 (0.44-0.94)
Southern	Ref	Ref	Ref
**Sub-region:year***			
Western and Central:Year	1.01 (0.97-1.05)	1.05 (0.99-1.12)	0.89 (0.78-1.02)
Eastern:Year	1.07 (1.05-1.10)	1.02 (0.99-1.05)	0.94 (0.89-1.00)
Southern:Year	1.01 (0.99-1.03)	1.02 (0.99-1.05)	0.96 (0.92-1.01)
**Sub-region:sex†**			
Western and Central:Male	0.74 (0.50-1.10)	0.90 (0.46-1.74)	0.00 (0.00-Inf)
Eastern:Male	0.73 (0.65-0.82)	0.57 (0.47-0.69)	0.58 (0.49-0.67)
Southern:Male	0.64 (0.59-0.69)	0.62 (0.54-0.72)	0.26 (0.22-0.30)
**Population**			
ANC attendees	Ref	Ref	Ref
FP attendees	0.98 (0.64-1.51)	0.97 (0.51-1.85)	0.19 (0.03-1.08)
GYN attendees	0.92 (0.52-1.61)	0.50 (0.20-1.27)	0.64 (0.29-1.40)
PHC/OPD attendees	0.76 (0.54-1.06)	1.15 (0.70-1.90)	0.83 (0.46-1.50)
Students	0.84 (0.55-1.31)	0.95 (0.48-1.88)	1.71 (0.90-3.25)
Community members	0.95 (0.66-1.37)	0.83 (0.48-1.43)	0.49 (0.19-1.23)
Prevention trial participants	0.83 (0.58-1.17)	0.99 (0.60-1.65)	0.82 (0.39-1.74)
Population-representative survey participants	0.93 (0.61-1.42)	1.14 (0.62-2.08)	0.71 (0.32-1.56)
**Age group**			
Adult	Ref	Ref	Ref
Youth	1.21 (0.92-1.59)	1.14 (0.78-1.68)	0.44 (0.24-0.82)
**HIV status**			
Non-stratified	Ref	Ref	Ref
HIV negative	1.45 (1.10-1.92)	1.07 (0.72-1.60)	0.64 (0.36-1.14)
**Model variance**			
τ^2 fixed	0.51	0.26	6.56
τ^2 random	0.29	0.54	0.48
**Number groups**			
Number studies	127	115	56
Number observations	188	176	68

*Midpoint year between start and end of data collection period; centred at 2012. †Reference sex is female. aPR: adjusted prevalence ratio, 95% CI: 95% confidence interval, NAAT: nucleic acid amplification test, τ^2: variance.

Table S12: Adjusted prevalence ratios for chlamydia, gonorrhoea, and trichomoniasis in sub-Saharan Africa, estimated via log-binomial generalised linear mixed-effects models: *within-study sensitivity analysis using observations unadjusted for diagnostic test performance*

**Variable**	**Chlamydia aPR (95% CI)**	**Gonorrhoea aPR (95% CI)**	**Trichomoniasis aPR (95% CI)**
**Intercept**	0.12 (0.08-0.19)	0.04 (0.02-0.08)	0.22 (0.18-0.25)
**Sub-region**			
Western and Central	0.28 (0.13-0.57)	0.45 (0.15-1.35)	0.35 (0.23-0.52)
Eastern	0.44 (0.28-0.67)	0.48 (0.24-0.95)	0.80 (0.63-1.02)
Southern	Ref	Ref	Ref
**Sub-region:year***			
Western and Central:Year	1.03 (0.95-1.11)	1.19 (1.05-1.35)	1.02 (1.00-1.04)
Eastern:Year	1.07 (1.03-1.10)	1.00 (0.95-1.05)	0.95 (0.80-1.12)
Southern:Year	1.05 (1.00-1.10)	1.04 (0.96-1.13)	0.90 (0.86-0.94)
**Sex**			
Female	Ref	Ref	Ref
Male	0.67 (0.63-0.71)	0.59 (0.52-0.66)	0.34 (0.31-0.38)
**Age group**			
Adult	Ref	Ref	Ref
Youth	1.08 (0.72-1.62)	1.04 (0.56-1.93)	0.38 (0.27-0.54)
**HIV status**			
Non-stratified	Ref	Ref	Ref
HIV negative	1.14 (0.73-1.77)	1.17 (0.59-2.32)	0.60 (0.26-1.40)
**Diagnostic test**			
NAAT	Ref	Ref	Ref
Culture	-	6.18 (1.55-24.62)	2.07 (1.31-3.25)
DFA	1.71 (0.55-5.31)	-	-
Rapid antigen test	-	-	1.13 (0.28-4.62)
Wet mount	-	-	0.00 (0.00-Inf)
**Model variance**			
τ^2 fixed	0.61	0.66	33.86
τ^2 random	0.15	0.35	<0.01
**Number groups**			
Number studies	26	26	12
Number observations	52	52	24

*Midpoint year between start and end of data collection period; centred at 2012. aPR: adjusted prevalence ratio, 95% CI: 95% confidence interval, DFA: direct fluorescent antibody, NAAT: nucleic acid amplification test, τ^2: variance.

Table S13: Adjusted prevalence ratios for chlamydia, gonorrhoea, and trichomoniasis in sub-Saharan Africa, estimated via log-binomial generalised linear mixed-effects models: *within-study sensitivity analysis using NAAT-diagnosed observations adjusted for test performance*

**Variable**	**Chlamydia aPR (95% CI)**	**Gonorrhoea aPR (95% CI)**	**Trichomoniasis aPR (95% CI)**
**Intercept**	0.13 (0.08-0.21)	0.04 (0.02-0.08)	0.21 (0.17-0.26)
**Sub-region**			
Western and Central	0.28 (0.12-0.62)	0.54 (0.17-1.70)	0.48 (0.30-0.77)
Eastern	0.43 (0.27-0.69)	0.52 (0.25-1.05)	1.18 (0.85-1.65)
Southern	Ref	Ref	Ref
**Sub-region:year***			
Western and Central:Year	1.04 (0.95-1.12)	1.18 (1.04-1.33)	-
Eastern:Year	1.07 (1.03-1.10)	1.00 (0.95-1.05)	1.07 (0.82-1.40)
Southern:Year	1.05 (1.00-1.11)	1.05 (0.97-1.14)	0.89 (0.84-0.95)
**Sex**			
Female	Ref	Ref	Ref
Male	0.60 (0.56-0.65)	0.74 (0.65-0.84)	0.25 (0.22-0.30)
**Age group**			
Adult	Ref	Ref	Ref
Youth	1.17 (0.75-1.81)	1.24 (0.65-2.36)	0.35 (0.22-0.56)
**HIV status**			
Non-stratified	Ref	Ref	Ref
HIV negative	1.08 (0.66-1.75)	1.05 (0.51-2.18)	0.74 (0.20-2.76)
**Model variance**			
τ^2 fixed	0.70	0.49	1.09
τ^2 random	0.17	0.39	<0.01
**Number groups**			
Number studies	25	25	8
Number observations	50	50	16

*Midpoint year between start and end of data collection period; centred at 2012. †Reference sex is female. aPR: adjusted prevalence ratio, 95% CI: 95% confidence interval, NAAT: nucleic acid amplification test, τ^2: variance.

Table S14: Adjusted prevalence ratios for chlamydia, gonorrhoea, and trichomoniasis in sub-Saharan Africa, estimated via log-binomial generalised linear mixed-effects models: *within-study sensitivity analysis using NAAT-diagnosed observations unadjusted for test performance*

**Variable**	**Chlamydia aPR (95% CI)**	**Gonorrhoea aPR (95% CI)**	**Trichomoniasis aPR (95% CI)**
**Intercept**	0.12 (0.08-0.19)	0.04 (0.02-0.08)	0.21 (0.18-0.25)
**Sub-region**			
Western and Central	0.28 (0.13-0.58)	0.45 (0.15-1.37)	0.37 (0.25-0.55)
Eastern	0.43 (0.28-0.68)	0.48 (0.24-0.96)	0.80 (0.63-1.01)
Southern	Ref	Ref	Ref
**Sub-region:year***			
Western and Central:Year	1.03 (0.95-1.11)	1.19 (1.05-1.35)	-
Eastern:Year	1.07 (1.03-1.10)	1.00 (0.95-1.05)	0.95 (0.80-1.13)
Southern:Year	1.05 (0.99-1.10)	1.04 (0.96-1.13)	0.90 (0.86-0.94)
**Sex**			
Female	Ref	Ref	Ref
Male	0.66 (0.62-0.71)	0.59 (0.52-0.66)	0.37 (0.33-0.41)
**Age group**			
Adult	Ref	Ref	Ref
Youth	1.08 (0.72-1.63)	1.04 (0.56-1.95)	0.38 (0.26-0.54)
**HIV status**			
Non-stratified	Ref	Ref	Ref
HIV negative	1.14 (0.72-1.79)	1.17 (0.58-2.35)	0.61 (0.26-1.43)
**Model variance**			
τ^2 fixed	0.63	0.58	0.79
τ^2 random	0.15	0.36	<0.01
**Number groups**			
Number studies	25	25	8
Number observations	50	50	16

*Midpoint year between start and end of data collection period; centred at 2012. †Reference sex is female. aPR: adjusted prevalence ratio, 95% CI: 95% confidence interval, NAAT: nucleic acid amplification test, τ^2: variance.

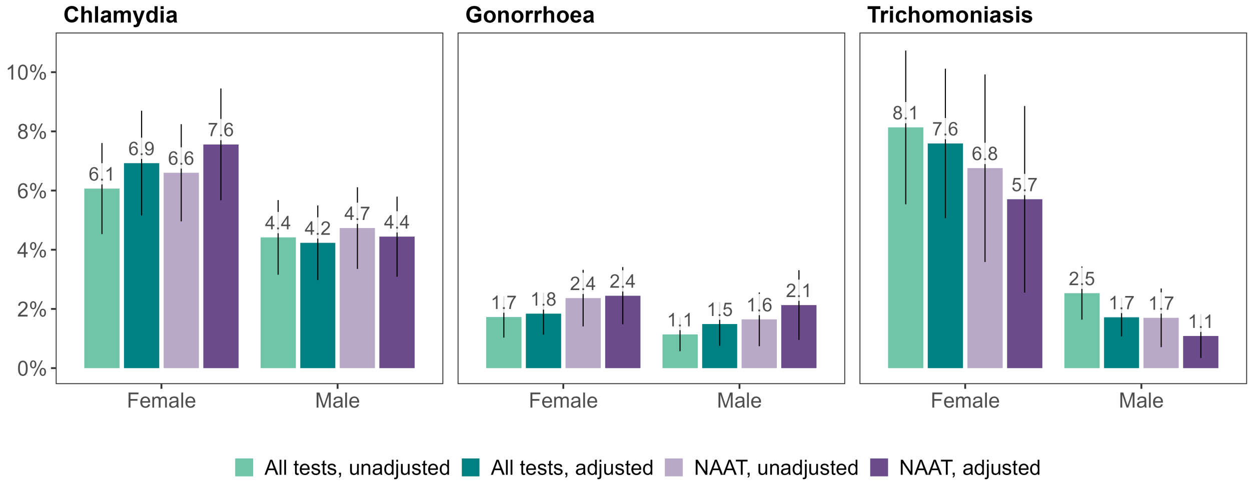

Figure S2: Sexually transmitted infection prevalence in sub-Saharan Africa in 2020, with and without accounting for diagnostic test performance.

Estimates of chlamydia, gonorrhoea, and trichomoniasis prevalence by sex for sub-Saharan Africa in 2020. Sub-regional estimates generated using log-binomial generalised linear mixed-effects models for each infection, using either observations as reported or adjusted for diagnostic test performance, with all diagnostic tests or NAAT only. Sub-Saharan African estimates represent sex-matched population-weighted means. Bars and error lines depict mean prevalence estimates with 95% confidence intervals. NAAT: Nucleic acid amplification test.

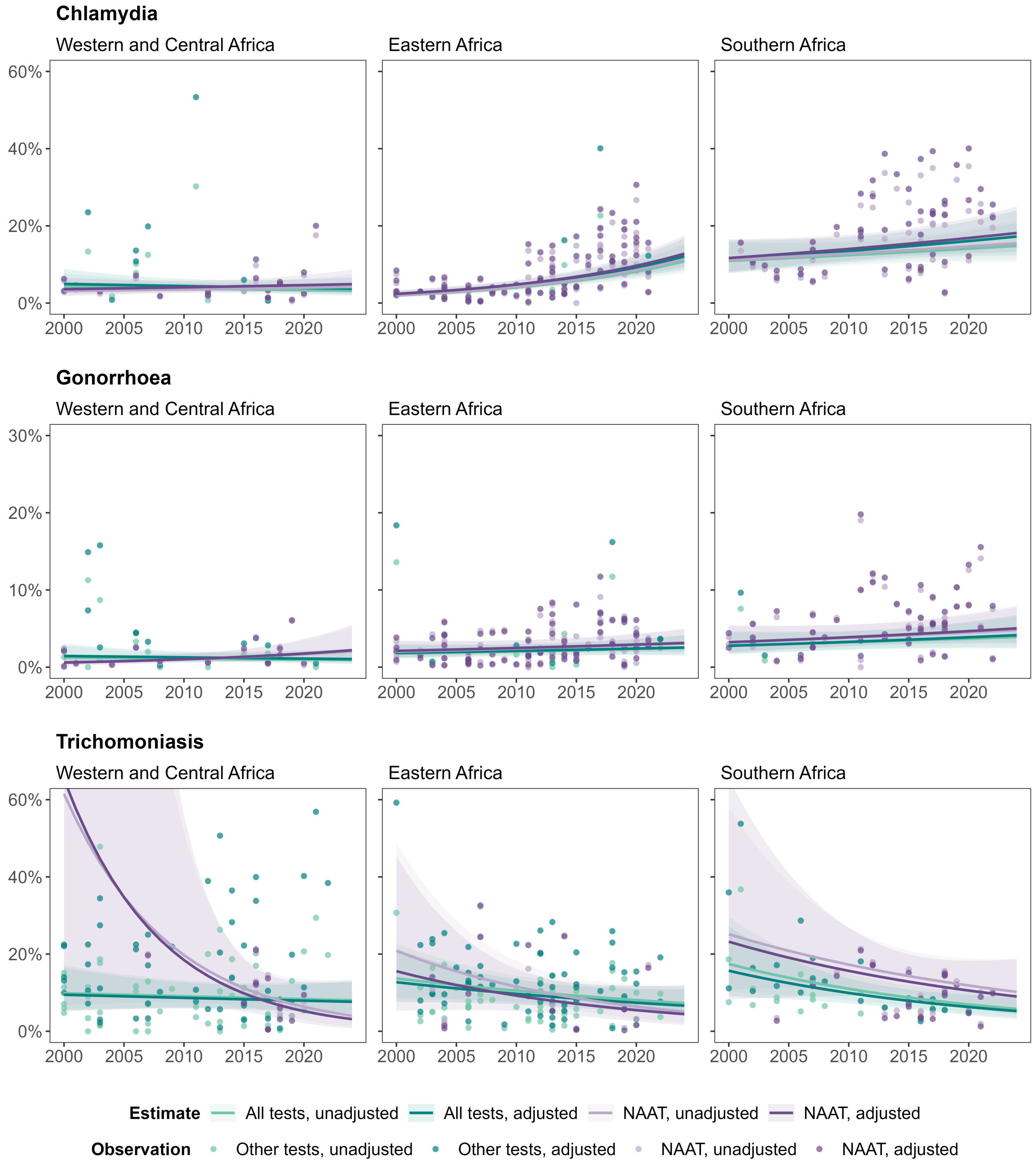

Figure S3: Sexually transmitted infection prevalence among females in sub-Saharan Africa between 2000 and 2024, with and without accounting for diagnostic test performance.

Estimates of chlamydia, gonorrhoea, and trichomoniasis prevalence among females between 2000 and 2024. Sub-regional estimates generated using log-binomial generalised linear mixed-effects models for each infection, using either observations as reported or adjusted for diagnostic test performance, with all diagnostic tests or NAAT only. Lines and shading depict mean prevalence estimates with 95% confidence intervals. Points represent study observations. NAAT: Nucleic acid amplification test.

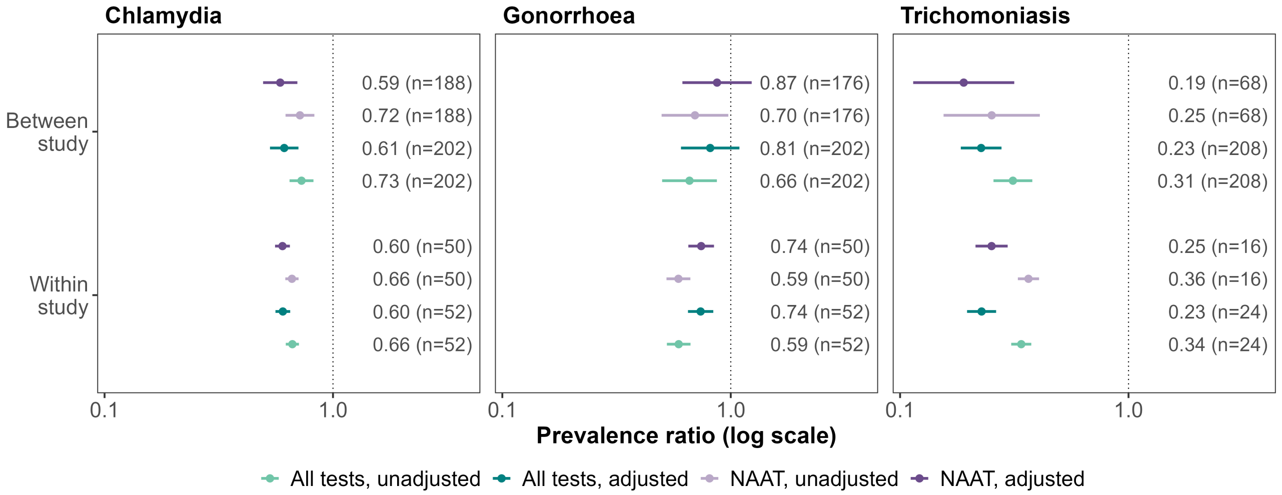

Figure S4: Sexually transmitted infection male-to-female prevalence ratio estimates in sub-Saharan Africa, with and without accounting for diagnostic test performance.

Male-to-female prevalence ratios for chlamydia, gonorrhoea, and trichomoniasis. Ratios estimated using log-binomial generalized linear mixed-effects models per infection, using either observations as reported or adjusted for diagnostic test performance, with all diagnostic tests or NAAT only. Models for within-study ratios used study observations among both sexes and between-study ratios used all study observations. Points and error lines depict population-weighted mean ratios and 95% confidence intervals for sub-Saharan Africa in 2020.

Table S15: Summary of critical appraisal criteria across studies

Criteria	Chlamydia (N = 139)	Gonorrhoea (N = 140)	Trichomoniasis (N = 162)	Total (N = 211)
**Selection bias: Participant sampling**				
Lower risk	19 (13.7%)	21 (15.0%)	29 (17.9%)	37 (17.5%)
Higher risk	120 (86.3%)	119 (85.0%)	133 (82.1%)	174 (82.5%)
**Selection bias: Participant characterisation**				
Lower risk	64 (46.0%)	64 (45.7%)	58 (35.8%)	78 (37.0%)
Higher risk	75 (54.0%)	76 (54.3%)	104 (64.2%)	133 (63.0%)
**Measurement bias: Diagnostic method consistency**				
Lower risk	135 (97.1%)	136 (97.1%)	151 (93.2%)	198 (93.8%)
Higher risk	4 (2.9%)	4 (2.9%)	11 (6.8%)	13 (6.2%)
**Precision: Sample size adequacy**				
Lower risk	135 (97.1%)	135 (96.4%)	153 (94.4%)	201 (95.3%)
Higher risk	4 (2.9%)	5 (3.5%)	9 (5.6%)	9 (4.7%)

Table S16: Adjusted prevalence ratios for chlamydia, gonorrhoea, and trichomoniasis in sub-Saharan Africa, estimated via log-binomial generalised linear mixed-effects models: *assessment of critical appraisal criteria using observations adjusted for diagnostic test performance*

Variable	Chlamydia aPR (95% CI)	Gonorrhoea aPR (95% CI)	Trichomoniasis aPR (95% CI)
**Intercept**	0.17 (0.10-0.26)	0.03 (0.01-0.05)	0.09 (0.05-0.14)
**Sub-region**			
Western and Central	0.31 (0.22-0.43)	0.37 (0.24-0.58)	0.78 (0.55-1.11)
Eastern	0.38 (0.34-0.41)	0.63 (0.54-0.72)	0.98 (0.82-1.17)
Southern	Ref	Ref	Ref
**Sub-region:year***			
Western and Central:Year	0.99 (0.95-1.03)	0.98 (0.93-1.04)	1.00 (0.96-1.03)
Eastern:Year	1.07 (1.05-1.09)	1.01 (0.98-1.04)	0.97 (0.94-1.00)
Southern:Year	1.02 (1.00-1.04)	1.02 (0.99-1.05)	0.96 (0.92-1.00)
**Sub-region:sex†**			
Western and Central:Male	0.66 (0.44-0.98)	1.15 (0.56-2.35)	0.14 (0.09-0.20)
Eastern:Male	0.61 (0.53-0.70)	0.69 (0.57-0.85)	0.36 (0.30-0.44)
Southern:Male	0.60 (0.55-0.66)	0.74 (0.63-0.87)	0.15 (0.11-0.20)
**Population**			
ANC attendees	Ref	Ref	Ref
FP attendees	1.00 (0.66-1.52)	0.96 (0.54-1.71)	0.68 (0.40-1.17)
GYN attendees	1.07 (0.59-1.93)	0.84 (0.41-1.73)	1.32 (0.79-2.20)
PHC/OPD attendees	0.77 (0.54-1.09)	1.40 (0.87-2.23)	0.93 (0.60-1.46)
Students	0.97 (0.62-1.51)	1.07 (0.53-2.13)	1.23 (0.75-2.03)
Community members	0.91 (0.61-1.36)	1.07 (0.63-1.82)	0.94 (0.47-1.91)
HIV/STI prevention trial participants	0.88 (0.61-1.28)	1.21 (0.74-1.99)	1.04 (0.63-1.72)
Population-representative survey participants	0.80 (0.47-1.36)	1.34 (0.67-2.71)	1.14 (0.57-2.26)
**Age group**			
Adult	Ref	Ref	Ref
Youth	1.25 (0.93-1.67)	1.32 (0.90-1.94)	0.55 (0.34-0.89)
**HIV status**			
Non-stratified	Ref	Ref	Ref
HIV negative	1.34 (0.99-1.80)	0.96 (0.64-1.45)	0.94 (0.64-1.37)
**Diagnostic test**			
NAAT	Ref	Ref	Ref
Culture	-	1.62 (1.01-2.59)	1.27 (0.81-1.99)
DFA	0.61 (0.28-1.36)	-	-
ELISA	2.35 (0.89-6.16)	-	-
Rapid antigen test	3.00 (1.81-5.00)	0.53 (0.06-4.60)	0.85 (0.45-1.62)
Wet mount	-	-	1.55 (1.08-2.24)
**Risk of selection bias: participant sampling**			
Lower risk	Ref	Ref	Ref
Higher risk	0.88 (0.60-1.29)	1.29 (0.79-2.11)	0.81 (0.55-1.21)

Continued...

Variable	Chlamydia aPR (95% CI)	Gonorrhoea aPR (95% CI)	Trichomoniasis aPR (95% CI)
**Risk of selection bias: participant characterisation**			
Lower risk	Ref	Ref	Ref
Higher risk	0.91 (0.72-1.14)	1.00 (0.73-1.36)	1.52 (1.22-1.89)
**Risk of measurement bias: diagnostic method consistency**			
Lower risk	Ref	Ref	Ref
Higher risk	1.64 (0.91-2.94)	1.24 (0.59-2.62)	1.10 (0.66-1.83)
**Risk of poor precision: sample size adequacy**			
Lower risk	Ref	Ref	Ref
Higher risk	1.37 (1.06-1.76)	1.93 (1.32-2.82)	1.52 (0.95-2.43)
**Model variance**			
τ^2 fixed	0.56	0.22	0.45
τ^2 random	0.34	0.57	0.65
**Number groups**			
Number studies	139	140	162
Number observations	202	202	207

*Midpoint year between start and end of data collection period; centred at 2012. †Reference sex is female. aPR: adjusted prevalence ratio, 95% CI: 95% confidence interval, DFA: direct fluorescent antibody, ELISA: enzyme-linked immunosorbent assay, NAAT: nucleic acid amplification test, τ^2: variance.

Bibliography

1. UN Statistics Division. Standard country or area codes for statistical use (M49). Geneva; 2023. Available from: https://unstats.un.org/unsd/methodology/m49/.

2. Flor M, Weiß M, Selhorst T, Müller-Graf C, Greiner M. Comparison of Bayesian and frequentist methods for prevalence estimation under misclassification. BMC public health. 2020 12;20(1):1135.

3. Speybroeck N, Devleesschauwer B, Joseph L, Berkvens D. Misclassification errors in prevalence estimation: Bayesian handling with care. International Journal of Public Health. 2013;58(5):791-5.

4. World Health Organization. Laboratory tests for the detection of reproductive tract infections. Geneva; 1999.

5. World Health Organization. Prevalence and incidence of selected sexually transmitted infections: Methods and results used by WHO to generate 2005 estimates. Geneva; 2011.

6. Grillo-Ardila CF, Torres M, Gaitán HG. Rapid point of care test for detecting urogenital Chlamydia trachomatis infection in nonpregnant women and men at reproductive age. The Cochrane database of systematic reviews. 2020 1;1(1).

7. Zhou Y, Jiang TT, Li J, Yin YP, Chen XS. Performance of point-of-care tests for the detection of chlamydia trachomatis infections: A systematic review and meta-analysis. EClinicalMedicine. 2021 7;37.

8. Watchirs Smith LA, Hillman R, Ward J, Whiley DM, Causer L, Skov S, et al. Point-of-care tests for the diagnosis of Neisseria gonorrhoeae infection: a systematic review of operational and performance characteristics. Sexually Transmitted Infections. 2013 6;89(4):320-6.

9. Gaydos CA, Klausner JD, Pai NP, Kelly H, Coltart C, Peeling RW. Rapid and point-of-care tests for the diagnosis of Trichomonas vaginalis in women and men. Sexually Transmitted Infections. 2017 12;93(S4):S31-5.

10. Michalow J, Walters MK, Olanrewaju E, Wybrant M, Davies B, Kufa T, et al. Aetiology of vaginal discharge, urethral discharge, and genital ulcer in sub-Saharan Africa: A systematic review and meta-regression. PLOS Medicine. 2024 5;21(5):e1004385.

11. Munn Z, Moola S, Lisy K, Riitano D, Tufanaru C. Chapter 5: Systematic reviews of prevalence and incidence. In: Aromataris E, Munn Z, editors. JBI Manual for Evidence Synthesis. JBI; 2020. .

12. Lagarde E, Congo Z, Meda N, Baya B, Yaro S, Sangli G, et al. Epidemiology of HIV infection in urban Burkina Faso. International Journal of STD & AIDS. 2004 6;15(6):395-402.

13. Lafort Y, Sawadogo Y, Delvaux T, Vuylsteke B, Laga M. Should family planning clinics provide clinical services for sexually transmitted infections? A case study from Côte d'Ivoire. Tropical Medicine and International Health. 2003 6;8(6):552-60.

14. Aboyeji A, Nwabuisi C. Prevalence of sexually transmitted diseases among pregnant women in Ilorin, Nigeria. Journal of Obstetrics and Gynaecology. 2003 11;23(6):637-9.

15. Donbraye E, Donbraye-Emmanuel OB, Okonko I, Okedeji IO, Alli JA, Nwanze J. Detection and prevalence of Trichomonas vaginalis among pregnant women in Ibadan, Southwestern Nigeria. World applied sciences journal. 2010;12(12):1512-7.

16. Apea-Kubi KA, Yamaguchi S, Sakyi B, Kishimoto T, Ofori-Adjei D, Hagiwara T. Neisseria gonorrhoea, Chlamydia trachomatis, and Treponema pallidum infection in antenatal and gynecological patients at Korle-Bu Teaching Hospital, Ghana. Japanese journal of infectious diseases. 2004;57(6):253–256.

17. Apea-Kubi KA, Sakyi B, Yamaguchi S, Ofori-Adjei D. Bacterial vaginosis, Candida albicans and Trichomonas vaginalis infection in antenatal and gynaecological patients in Ghana. Tropical Journal of Obstetrics and Gynaecology. 2005 5;22(2).

18. Adejuwon AO, Adejuwon CA. Infection among women using intra-uterine contraceptive devices. Journal of Medical Sciences. 2005 3;5(2):120-4.

19. Obiajuru I, Ogbulie J. Comparative study of the prevalence of sexually transmitted diseases between pregnant and non-pregnants women in Imo State, Nigeria. Global Journal of Pure and Applied Sciences. 2005;11(3).

20. Tukur J, Fwacs M, Shittu SO, Drh MF, Abdul AM. A case control study of active genital Chlamydia trachomatis infection among patients with tubal infertility in northern Nigeria; 2006.

21. Balaka B, Agbèrè A, Dagnra A, Baeta S, Kessie K, Assimadi K. Genital bacterial carriage during the last trimester of pregnancy and early-onset neonatal sepsis. Archives de pédiatrie: organe officiel de la Société française de pédiatrie. 2005;12(5):514–519.

22. Kirakoya-Samadoulogou F, Nagot N, Defer M, Yaro S, Meda N, Robert A. Bacterial Vaginosis Among Pregnant Women in Burkina Faso. Sexually transmitted diseases. 2008;35(12):985–989.

23. Siemer J, Theile O, Larbi Y, Fasching PA, Danso KA, Kreienberg R, et al. Chlamydia trachomatis Infection as a Risk Factor for Infertility among Women in Ghana, West Africa; 2008.

24. Chigbu LN, Aluka C, Eke RA. Trichomoniasis as an Indicator for Existing Sexually Transmitted Infections in Women in Aba, Nigeria; 2006. 1.

25. Inabo HI, Adewumi AA, Ishaya A. Prevalence of T. vaginalis in pregnant women attending antenatal clinics in Kaduna, Nigeria. Global Journal of Pure and Applied Sciences. 2006;12(3):323-5.

26. Jatau ED, Olonitola OS, Olayinka AT. Prevalence of Trichomonas Infection among Women Attending Antenatal Clinics in; 2006. 4.

27. Sagay AS, Kapiga SH, Imade GE, Sankale JL, Idoko J, Kanki P. HIV infection among pregnant women in Nigeria. International Journal of Gynecology and Obstetrics. 2005;90(1):61-7.

28. Yirenya-Tawiah D, Annang TN, Apea-Kubi KA, Lomo G, Mensah D, Akyeh L, et al. Chlamydia Trachomatis and Neisseria Gonorrhoeae prevalence among women of reproductive age living in urogenital schistosomiasis endemic area in Ghana. BMC Research Notes. 2014 12;7(1):349.

29. Fayemiwo SA, Makanjuola OB, Fatiregun AA. Vulvo-vaginal candidosis in a cohort of hormonal contraceptive users in Ibadan, Nigeria. African Journal of Clinical and Experimental Microbiology. 2018 11;19(1):38.

30. Omoregie R, Eghafona NO. Urinary tract infection among asymptomatic HIV patients in Benin City, Nigeria. British Journal of Biomedical Science. 2009;66(4):190-3.

31. Kengne P, Dosso M. Evaluation of reverse hybridization diagnostic strategy for simultaneous detection of Chlamydia trachomatis and Neisseria gonorrhoeae. Sciences and Medicine in Africa. 2010;2(1).

32. Chinyere O. Trichomonas vaginalis Associated with Adverse Pregnancy Outcomes: Implications for Maternal Health Care Delivery System in South Eastern Nigeria. British Journal of Medicine and Medical Research. 2012 1;2(4):568-74.

33. Niemogha MT, Smith SI, Goodluck HA, Gbaja-biamila T, Fesobi T, Umurhuru A, et al. Chlamydia and Vaginitis in Sexually Active Females: Classical Identification Methods for Effective Control; 2010. 2.

34. Béhanzin L, Diabaté S, Minani I, Lowndes CM, Boily MC, Labbé AC, et al. Decline in HIV Prevalence among Young Men in the General Population of Cotonou, Benin, 1998–2008. PLoS ONE. 2012 8;7(8):43818.

35. Usanga V, Abia-Bassey L, Inyang-Etoh P, Udoh S, Ani F, Archibong E. Prevalence of sexually transmitted diseases in pregnant and non-pregnant women in Calabar, Cross River State, Nigeria. The Internet Journal of Gynecology and Obstetrics. 2011;14(2).

36. Sam-Wobo SO, Ajao OK, Adeleke MA, Ekpo UF. Trichomoniasis among ante-natal attendees in a tertiary health facility. Munis Entomology and Zoology. 2012;7(1).

37. Arinze AH, Onyebuchi N, Isreal J. Genital chlamydia trachomatis infection among female undergraduate students of University of Port Harcourt, Nigeria. Nigerian Medical Journal. 2014;55(1):9.

38. Bolaji O, Adejare FO, Adeyeba OA, Ojurongbe O. Comparison of methods of diagnosis of Trichomoniasis in pregnancy among antenatal patients in some parts of Oyo state, Nigeria. International Journal of Pharma Medicine and Biological Sciences. 2013;2(3):5–12.

39. Tchelougou D, Karou DS, Kpotsra A, Balaka A, Assih M, Bamoke M, et al. [Vaginal infections in pregnant women at the Regional Hospital of Sokode (Togo) in 2010 and 2011]. Medecine et sante tropicales. 2013;23(1):49-54.

40. Völker F, Cooper P, Bader O, Uy A, Zimmermann O, Lugert R, et al. Prevalence of pregnancy-relevant infections in a rural setting of Ghana. BMC Pregnancy and Childbirth. 2017 6;17(1).

41. Adesiji YO, Iyere SI, Ogah IJ. Low prevalence of Chlamydia trachomatis infection in women from southern Nigeria. Nitte University Journal of Health and Allied Sciences. 2015 3;05(01):004-8.

42. Olowe O, Makanjuola O, Olowe R, Adekanle D. Prevalence of vulvovaginal candidiasis, trichomoniasis and bacterial vaginosis among pregnant women receiving antenatal care in Southwestern Nigeria. European Journal of Microbiology and Immunology. 2014 12;4(4):193-7.

43. Samuel BO, Soliu AT, Bello SA, Olusola O, Adegboyega AO. Prevalence of Trichomonas vaginalis among pregnant women in selected hospitals in Ilorin metropolis. Journal of Pharmaceutical & Scientific Innovation. 2015 4;4(2):108-11.

44. Etuketu IM, Mogaji HO, Alabi OM, Adeniran AA, Oluwol AS, Ekpo UF. Prevalence and risk factors of Trichomonas vaginalis infection among pregnant women receiving antenatal care in Abeokuta, Nigeria. African Journal of Infectious Diseases. 2015 5;9(2):51-6.

45. Nnaemeka AM, Iyioku UU, Oluwabusuyi OJ. Co-infection of Trichomonas vaginalis and Candida albcians among women of childbearing age in Ebonyi LGA, Ebonyistate, Nigeria. Asian Journal of Microbiology Biotechnology and Environmental Sciences. 2016;18(4).

46. Olusegun-Joseph TS, Killaney VM. Survey of possible pathogenic organisms found in urine and vaginal swab samples of selected female population in Lagos, Nigeria. International Journal of Biological and Chemical Sciences. 2017 3;10(4):1840.

47. Akinbo FO, Oronsaye IS. Trichomonas vaginalis infection among adolescent girls in some secondary schools in Benin city, Edo state, Nigeria. African Journal of Clinical and Experimental Microbiology. 2017;18(4).

48. Oyeyemi OT, Fadipe O, Oyeyemi IT. Trichomonas vaginalis infection in Nigerian pregnant women and risk factors associated with sexually transmitted infections. International Journal of STD and AIDS. 2016 11;27(13):1187-93.

49. Wokem G, Ndukwu C. Re-evaluation of vulvovaginal trichomoniasis among women in Niger delta region, Nigeria. Global Journal of Pure and Applied Sciences. 2015;21(1):13-9.

50. Sangaré I, Cissé M, Sirima C, Sanou S, Bazié WW, Konaté I, et al. Prevalence and factors associated of Trichomonas vaginalis infection among pregnant women in Bobo-Dioulasso, Burkina Faso. Annals of parasitology. 2021;67(2):321-8.

51. Konadu DG, Owusu-Ofori A, Yidana Z, Boadu F, Iddrisu LF, Adu-Gyasi D, et al. Prevalence of vulvovaginal candidiasis, bacterial vaginosis and trichomoniasis in pregnant women attending antenatal clinic in the middle belt of Ghana. BMC Pregnancy and Childbirth. 2019;19(1):341.

52. Alexander SI, Idowu ET, Otubanjo OA, Ajayi MB. Trichomoniasis among pregnant women in Ifako Ijaiye, Shomolu
}and Agege Local Government Areas of Lagos State, Nigeria. Nigerian Journal of Parasitology. 2018;39(1):96-100.

53. Ebhodaghe BI, Ako-Nai KA, Aderoba AK. Evaluation of risk factors in MTCT among HIV-seropositive pregnant women in selected centers in Akure, South Western Nigeria. Annals of Tropical Medicine and Public Health. 2017 1;10(1):165-81.

54. Asmah RH, Blankson HNA, Seanefu KA, Obeng-Nkrumah N, Awuah-Mensah G, Cham M, et al. Trichomoniasis and associated co-infections of the genital tract among pregnant women presenting at two hospitals in Ghana. BMC Women's Health. 2017 12;17(1):130.

55. Squire DS, Lymbery AJ, Walters J, Ahmed H, Asmah RH, Andrew Thompson RC. Trichomonas vaginalis infection in southern Ghana: Clinical signs associated with the infection. Transactions of the Royal Society of Tropical Medicine and Hygiene. 2019 7;113(7):359-69.

56. Cowley G, Milne G, Teixeira da Silva E, Nakutum J, Rodrigues A, Vasileva H, et al. Prevalence of and risk factors for curable sexually transmitted infections on Bubaque Island, Guinea Bissau. Sexually Transmitted Infections. 2021 2;97(1):51-5.

57. Ezeanya CC, Agbakoba NR, Enweani IB, Oguejiofor C. Predominance of cervicitis agents with minimal testing rate within the student population in Benin city, Nigeria. Journal of Obstetrics and Gynaecology. 2019 8;39(6):840-4.

58. Ukatu VE, Nebenu I, Chilaka CG, Attah OA. Evaluating Trichomonas vaginalis infection in pregnant women from two health facilities, Sokoto, Nigeria. Nigerian Journal of Parasitology. 2019 3;40(1):46-50.

59. Kashibu E, Victor O, Ojumah I, Akafa R, Rikwentishe E. Trichomonas vaginalis infection: Prevalence and risk factors among ante-natal attendees in a tertiary facility in Taraba state, North-East Nigeria. Nigerian Journal of Parasitology. 2018 9;39(2):199-203.

60. Odaranle AI, Olajuyigbe OO, Adeoye-Isijola MO, Adedayo O, Coopoosamy RM. Microbial Flora and Antimicrobial Susceptibility of Microorganisms from Asymptomatic Contraceptive Users and Non-Users of Reproductive Age. Journal of Clinical and Diagnostic Research. 2020;14(1):01– 07.

61. Isara A, Baldeh AK. Prevalence of sexually transmitted infections among pregnant women attending antenatal clinics in west coast region of the gambia. African Health Sciences. 2021 6;21(2):585-92.

62. Jary A, Teguete I, Sidibé Y, Kodio A, Dolo O, Burrel S, et al. Prevalence of cervical HPV infection, sexually transmitted infections and associated antimicrobial resistance in women attending cervical cancer screening in Mali. International Journal of Infectious Diseases. 2021 7;108:610-6.

63. Rasheed FA, Yakasai IA, Takai IU, Yusuf I, Ibrahim UM. Cervical cytopathological changes in pregnancy: An experience from a low resource setting. Annals of African medicine. 2021 7;20(3):212-21.

64. Ajani TA, Elikwu CJ, Fayemiwo SA, Nwadike V, Tayo B, Anaedobe CG, et al. Trichomonas vaginalis among asymptomatic undergraduate students in a private university in Ogun State, Nigeria. Annals of Ibadan postgraduate medicine. 2022 12;20(2):135-42.

65. Auta I, Ibrahim B, Henry D. Prevalence of Trichomonas vaginalis among pregnant women attending antenatal clinic in two health facilities within Kaduna metropolis, Kaduna, Nigeria. Science World Journal. 2020;15(1):2020.

66. Maureen EC, Chinyere U, Charity O N, Chidera O. Concomitant malarial infection and pathogenic microbiota in the reproductive tract of pregnant women in Orlu, Imo State, Nigeria. Asian Journal of Microbiology, Biotechnology and Environmental Sciences. 2022 6:408-12.

67. Lingani M, Zango SH, Valéa I, Bonko MdA, Samadoulougou SO, Rouamba T, et al. Malaria and curable sexually transmitted and reproductive tract coinfection among pregnant women in rural Burkina Faso. Tropical Medicine and Health. 2021 12;49(1).

68. Agabi YA, Kilson MD, Uneze SB, Ali M, Jwan ZH, Silas VT, et al. Candida albicans and Trichomonas vaginalis: High prevalence and risk factors in women attending a gynaecology clinic in Jos, Nigeria. Microbes and Infectious Diseases. 2023 8;4(3):1065-71.

69. Enwuru CA, Aiyedobgon AS, Ajayi MB, Osuolale KA. Bacterial vaginosis (BV) and Trichomonas vaginalis (TV) co-infection, and bacterial antibiogram profile of pregnant women studied in Lagos, Nigeria. BMC Women's Health. 2024 12;24(1).

70. Butcher R, Jarju S, Obayemi D, Bashorun AO, Vasileva H, Bransbury-Hare H, et al. Prevalence of five treatable sexually transmitted infections among women in Lower River region of The Gambia. BMC Infectious Diseases. 2023 12;23(1):1-8.

71. Ngom NS, Gassama O, Dieng A, Diakhaby EB, Ndiaye SML, Tine A, et al. Vaginal Carriage of Group B Streptococcus (GBS) in Pregnant Women, Antibiotic Sensitivity and Associated Risk Factors in Dakar, Senegal. Microbiology Insights. 2023 1;16:1-10.

72. Ngandjio A, Clerc M, Fonkoua MC, Thonnon J, Njock F, Pouillot R, et al. Screening of volunteer students in Yaounde (Cameroon, Central Africa) for Chlamydia trachomatis infection and genotyping of isolated C. trachomatis strains. Journal of Clinical Microbiology. 2003 9;41(9):4404-7.

73. Kinoshita-moleka R, Smith JS, Atibu J, Tshefu A, Hemingway-Foday J, Hobbs M, et al. Low prevalence of HIV and other selected sexually transmitted infections in 2004 in pregnant women from Kinshasa, the Democratic Republic of the Congo. Epidemiology and Infection. 2008 9;136(9):1290-6.

74. Mbu ER, Kongnyuy EJ, Mbopi-Keou FX, Tonye RN, Nana PN, Leke RJI. Gynaecological morbidity among HIV positive pregnant women in Cameroon. Reproductive Health. 2008;5(1).

75. Alexandre I, Justel M, Martinez P, Ortiz De Lejarazu R, Pastor JC. First attempt to implement ophthalmia neonatorum prophylaxis in Angola: Microorganisms, efficacy, and obstacles. Journal of Ophthalmology. 2015;2015.

76. Vieira-Baptista P, Grinceviciene S, Bellen G, Sousa C, Saldanha C, Broeck DV, et al. Genital Tract Infections in an Isolated Community: 100 Women of the Príncipe Island. Infectious Diseases in Obstetrics and Gynecology. 2017;2017.

77. Compain F, Nodjikouambaye ZA, Sadjoli D, Moussa AM, Adawaye C, Bouassa RSM, et al. Low Prevalence of Common Sexually Transmitted Infections Contrasting with High Prevalence of Mycoplasma Asymptomatic Genital Carriage: A Community-Based Cross-Sectional Survey in Adult Women Living in N'Djamena, Chad. The Open Microbiology Journal. 2019 9;13(1):222-9.

78. Nodjikouambaye ZA, Compain F, Sadjoli D, Mboumba Bouassa RS, Péré H, Veyer D, et al. Accuracy of curable sexually transmitted infections and genital mycoplasmas screening by multiplex real-time PCR using a self-collected veil among adult women in Sub-Saharan Africa. Infectious Diseases in Obstetrics and Gynecology. 2019 7;2019:1-15.

79. Gadoth A, Mvumbi G, Hoff NA, Musene K, Mukadi P, Ashbaugh HR, et al. Urogenital Schistosomiasis and Sexually Transmitted Coinfections among Pregnant Women in a Schistosome-Endemic Region of the Democratic Republic of Congo. The American journal of tropical medicine and hygiene. 2019 10;101(4):828-36.

80. Mbah CE, Jasani A, Aaron KJ, Akoachere JF, Tita ATN, Geisler WM, et al. Association between Chlamydia trachomatis, Neisseria gonorrhea, Mycoplasma genitalium, and Trichomonas vaginalis and Secondary Infertility in Cameroon: A case-control study. PLoS ONE. 2022 2;17(2 February).

81. Payne VK, Florence Cécile TT, Cedric Y, Christelle Nadia NA, José O. Risk Factors Associated with Prevalence of Candida albicans, Gardnerella vaginalis, and Trichomonas vaginalis among Women at the District Hospital of Dschang, West Region, Cameroon. International Journal of Microbiology. 2020;2020.

82. Ngombe Mouabata DFL, Boumba ALM, Massengo NRB, Pouki FS, Moukassa D, Ennaji MM. Prevalence of co-infection with human papillomavirus and Chlamydia trachomatis and risk factors associated with cervical cancer in Congolese women. Microbes and Infection. 2024 3;26(3):105287.

83. Eyong EEJ, Landred K, Njimated NONe, Katamssadan TH. Prevalence and risk factors of trichomoniasis in patients attending two medical centres in urban and rural areas in the North West Region, Cameroon. International Journal of Biological and Chemical Sciences. 2023 8;17(3):848-63.

84. Hawken MP, Melis RDJ, Ngombo DT, Mandaliya KN, Ng'ang'a LW, Price J, et al. Opportunity for Prevention of HIV and Sexually Transmitted Infections in Kenyan Youth: Results of a Population-Based Survey. JAIDS Journal of Acquired Immune Deficiency Syndromes. 2002 12;31(5):529-35.

85. Kaydos-Daniels SC, Miller WC, Hoffman I, Banda T, Dzinyemba W, Martinson F, et al. Validation of a urine-based PCR-enzyme-linked immunosorbent assay for use in clinical research settings to detect Trichomonas vaginalis in men. Journal of Clinical Microbiology. 2003 1;41(1):318-23.

86. Paz-Soldan V, Hoffman I, deGraft J, Bisika J, Kazembe P, Feluzi H, et al. Sexually Transmitted Infection (STI) screening, case and contact treatment, and condom promotion resulting in STI Reduction two years later in rural Malawi. Malawi Medical Journal. 2012 7;24(1):8-13.

87. Menéndez C, Castellsagué X, Renom M, Sacarlal J, Quintó L, Lloveras B, et al. Prevalence and risk factors of sexually transmitted infections and cervical neoplasia in women from a rural area of southern Mozambique. Infectious diseases in obstetrics and gynecology. 2010;2010.

88. Clift S, Anemona A, Watson-Jones D, Kanga Z, Ndeki L, Changalucha J, et al. Variations of HIV and STI prevalences within communities neighbouring new goldmines in Tanzania: importance for intervention design. Sexually Transmitted Infections. 2003 8;79(4):307-12.

89. Cowan FM, Langhaug LF, Mashungupa GP, Nyamurera T, Hargrove J, Jaffar S, et al. School based HIV prevention in Zimbabwe: feasibility and acceptability of evaluation trials using biological outcomes. AIDS. 2002 8;16(12):1673-8.

90. Van De Wijgert JHHM, Morrison CS, Brown J, Kwok C, Van Der Pol B, Chipato T, et al. Disentangling contributions of reproductive tract infections to hiv acquisition in african women. Sexually Transmitted Diseases. 2009 6;36(6):357-64.

91. Munjoma MW, Kurewa EN, Mapingure MP, Mashavave GV, Chirenje MZ, Rusakaniko S, et al. The prevalence, incidence and risk factors of herpes simplex virus type 2 infection among pregnant Zimbabwean women followed up nine months after childbirth; 2010.

92. Bailey RC, Moses S, Parker CB, Agot K, Maclean I, Krieger JN, et al. Male circumcision for HIV prevention in young men in Kisumu, Kenya: a randomised controlled trial. Lancet. 2007 2;369(9562):643-56.

93. Ghebremichael M, Paintsil E, Larsen U. Alcohol abuse, sexual risk behaviors, and sexually transmitted infections in women in moshi Urban district, Northern Tanzania. Sexually Transmitted Diseases. 2009 2;36(2):102-7.

94. Ghebremichael M, Paintsil E. High risk behaviors and sexually transmitted infections among men in Tanzania. AIDS and Behavior. 2011 7;15(5):1026-32.

95. Mapingure MP, Msuya S, Kurewa NE, Munjoma MW, Sam N, Chirenje MZ, et al. Sexual behaviour does not reflect HIV-1 prevalence differences: a comparison study of Zimbabwe and Tanzania. Journal of the International AIDS Society. 2010 1;13(1):45-5.

96. Msuya SE, Uriyo J, Hussain A, Mbizvo EM, Jeansson S, Sam NE, et al. Prevalence of sexually transmitted infections among pregnant women with known HIV status in northern Tanzania. Reproductive Health. 2009;6(1).

97. Celentano DD, Mayer KH, Pequegnat W, Abdala N, Green AM, Handsfield HH, et al. Prevalence of sexually transmitted diseases and risk behaviors from the NIMH collaborative HIV/STD prevention trial. International Journal of Sexual Health. 2010 10;22(4):272-84.

98. Mensch BS, Hewett PC, Helleringer S. Sexual Behavior and STI/HIV Status Among Adolescents in Rural Malawi: An Evaluation of the Effect of Interview Mode on Reporting NIH Public Access; 2008. 4.

99. Luján J, de Oñate WA, Delva W, Claeys P, Sambola F, Temmerman M, et al. Prevalence of sexually transmitted infections in women attending antenatal care in Tete province, Mozambique. South African medical journal. 2008 1;98(1):49-51.

100. Kamali A, Byomire H, Muwonge C, Bakobaki J, Rutterford C, Okong P, et al. A randomised placebo-controlled safety and acceptability trial of PRO 2000 vaginal microbicide gel in sexually active women in Uganda. Sexually Transmitted Infections. 2010;86(3):222.

101. Tann CJ, Mpairwe H, Morison L, Nassimu K, Hughes P, Omara M, et al. Lack of effectiveness of syndromic management in targeting vaginal infections in pregnancy in Entebbe, Uganda. Sexually Transmitted Infections. 2006 8;82(4):285-9.

102. Ramjee G, Kapiga S, Weiss S, Peterson L, Leburg C, Kelly C, et al. The Value of Site Preparedness Studies for Future Implementation of Phase 2/IIb/III HIV Prevention Trials. JAIDS Journal of Acquired Immune Deficiency Syndromes. 2008 1;47(1):93-100.

103. Venkatesh KK, Van Der Straten A, Mayer KH, Blanchard K, Ramjee G, Lurie MN, et al. African women recently infected with HIV-1 and HSV-2 have increased risk of acquiring neisseria gonorrhoeae and chlamydia trachomatis in the methods for improving reproductive health in Africa trial. Sexually Transmitted Diseases. 2011 6;38(6):562-70.

104. Gray RH, Kigozi G, Serwadda D, Makumbi F, Nalugoda F, Watya S, et al. The effects of male circumcision on female partners' genital tract symptoms and vaginal infections in a randomized trial in Rakai, Uganda. American Journal of Obstetrics and Gynecology. 2009;200(1):1-42.

105. Lingappa JR, Kahle E, Mugo N, Mujugira A, Magaret A, Baeten J, et al. Characteristics of HIV-1 discordant couples enrolled in a trial of HSV-2 suppression to reduce HIV-1 transmission: The Partners Study. PLoS ONE. 2009 4;4(4).

106. Chersich MF, Kley N, Luchters SMF, Njeru C, Yard E, Othigo MJ, et al. Maternal morbidity in the first year after childbirth in Mombasa Kenya; a needs assessment. BMC Pregnancy and Childbirth. 2009 11;9:51.

107. Guffey MB, Richardson B, Husnik M, Makanani B, Chilongozi D, Yu E, et al. HPTN 035 phase II/IIb randomised safety and effectiveness study of the vaginal microbicides BufferGel and 0.5% PRO 2000 for the prevention of sexually transmitted infections in women. Sexually Transmitted Infections. 2014 8;90(5):363-9.

108. Mccormack S, Ramjee G, Kamali A, Rees H, Crook AM, Gafos M, et al. PRO2000 vaginal gel for prevention of HIV-1 infection (Microbicides Development Programme 301): a phase 3, randomised, double-blind, parallel-group trial. Lancet. 2010;376:1329-66.

109. Crucitti T, Jespers V, Mulenga C, Khondowe S, Vandepitte J, Buvé A. Trichomonas vaginalis is Highly Prevalent in Adolescent Girls, Pregnant Women, and Commercial Sex Workers in Ndola, Zambia. Sexually Transmitted Diseases. 2010;37(4):223-7.

110. Otieno FO, Ndivo R, Oswago S, Pals S, Chen R, Thomas T, et al. Correlates of prevalent sexually transmitted infections among participants screened for an HIV incidence cohort study in Kisumu, Kenya. International Journal of STD and AIDS. 2015 3;26(4):225-37.

111. Mocumbi S, Gafos M, Munguambe K, Goodall R, McCormack S. High HIV prevalence and incidence among women in Southern Mozambique: Evidence from the MDP microbicide feasibility study. PloS one. 2017 3;12(3).

112. Lemme F, Doyle AM, Changalucha J, Andreasen A, Baisley K, Maganja K, et al. HIV Infection among Young People in Northwest Tanzania: The Role of Biological, Behavioural and Socio-Demographic Risk Factors. PLoS ONE. 2013 6;8(6).

113. Muvunyi CM, Dhont N, Verhelst R, Temmerman M, Claeys G, Padalko E. Chlamydia trachomatis infection in fertile and subfertile women in Rwanda: Prevalence and diagnostic significance of IgG and IgA antibodies testing. Human Reproduction. 2011;26(12):3319-26.

114. Rutherford GW, Anglemyer A, Bagenda D, Muyonga M, Lindan CP, Barker JL, et al. University students and the risk of HIV and other sexually transmitted infections in Uganda: The crane survey. International Journal of Adolescent Medicine and Health. 2014 5;26(2):209-15.

115. Ademe M, Kebede T, Fikrie N. Syndromic Management Approach and Wet Mount Microscopy of Trichomonas vaginalis in Pregnant Women and Sexually Transmitted Infection Clinic Attendants in Merawi Health Center, Merawi, Amhara Regional State, Ethiopia. International STD Research & Reviews. 2013 1;1(1):30-8.

116. Chiduo M, Theilgaard ZP, Bakari V, Mtatifikolo F, Bygbjerg I, Flanholc L, et al. Prevalence of sexually transmitted infections among women attending antenatal clinics in Tanga, north eastern Tanzania. International journal of STD & AIDS. 2012 5;23(5):325-9.

117. Downs JA, Van Dam GJ, Changalucha JM, Corstjens PLAM, Peck RN, De Dood CJ, et al. Association of schistosomiasis and HIV infection in Tanzania. American Journal of Tropical Medicine and Hygiene. 2012;87(5):868-73.

118. Jespers V, Crucitti T, Menten J, Verhelst R, Mwaura M, Mandaliya K, et al. Prevalence and Correlates of Bacterial Vaginosis in Different Sub-Populations of Women in Sub-Saharan Africa: A Cross-Sectional Study. PLoS ONE. 2014 10;9(10).

119. De Walque D, Dow WH, Nathan R, Abdul R, Abilahi F, Gong E, et al. Incentivising safe sex: a randomised trial of conditional cash transfers for HIV and sexually transmitted infection prevention in rural Tanzania. BMJ Open. 2012;2(1).

120. Lazenby GB, Taylor PT, Badman BS, McHaki E, Korte JE, Soper DE, et al. An association between trichomonas vaginalis and high-risk human papillomavirus in rural tanzanian women undergoing cervical cancer screening. Clinical Therapeutics. 2014 1;36(1):38-45.

121. Kiene SM, Lule H, Sileo KM, Silmi KP, Wanyenze RK. Depression, alcohol use, and intimate partner violence among outpatients in rural Uganda: Vulnerabilities for HIV, STIs and high risk sexual behavior. BMC Infectious Diseases. 2017 1;17(1).

122. Ogilvie GS, Mitchell S, Sekikubo M, Biryabarema C, Byamugisha J, Jeronimo J, et al. Results of a community-based cervical cancer screening pilot project using human papillomavirus self-sampling in Kampala, Uganda. International Journal of Gynecology and Obstetrics. 2013;122(2):118-23.

123. Eshete A, Mekonnen Z, Zeynudin A. Trichomonas vaginalis Infection among Pregnant Women in Jimma University Specialized Hospital, Southwest Ethiopia . ISRN Infectious Diseases. 2013 3;2013:1-5.

124. Kerubo E, Laserson KF, Otecko N, Odhiambo C, Mason L, Nyothach E, et al. Prevalence of reproductive tract infections and the predictive value of girls' symptom-based reporting: findings from a cross-sectional survey in rural western Kenya. Sexually Transmitted Infections. 2016 6;92(4):251-6.

125. Kinuthia J, Drake AL, Matemo D, Richardson BA, Zeh C, Osborn L, et al. HIV Acquisition During Pregnancy and Postpartum is Associated with Genital Infections and Partnership Characteristics: A Cohort Study. AIDS. 2015 9;29(15).

126. Ravindran J, Richardson BA, Kinuthia J, Unger JA, Drake AL, Osborn L, et al. Chlamydia, Gonorrhea, and Incident HIV Infection during Pregnancy Predict Preterm Birth Despite Treatment. Journal of Infectious Diseases. 2021 12;224(12):2085-93.

127. Nkhoma M, Ashorn P, Ashorn U, Dewey KG, Gondwe A, Mbotwa J, et al. Providing lipid-based nutrient supplement during pregnancy does not reduce the risk of maternal P falciparum parasitaemia and reproductive tract infections: A randomised controlled trial. BMC Pregnancy and Childbirth. 2017 1;17(1).

128. Hokororo A, Kihunrwa A, Hoekstra P, Kalluvya SE, Changalucha JM, Fitzgerald DW, et al. High Prevalence of Sexually-Transmitted Infections in Pregnant Adolescent Girls in Tanzania: a Multi-Community Cross-Sectional Study. Sexually transmitted infections. 2015 11;91(7):473.

129. Stephen S, Muchaneta-Kubara CGE, Munjoma MW, Mandozana G. Evaluation of Cortez Onestep Chlamydia RapicardTM Insta Test for the Detection of Chlamydia Trachomatis in Pregnant Women at Mbare Polyclinic in Harare, Zimbabwe. International Journal of MCH and AIDS. 2017 12;6(1):19.

130. Mulu W, Yimer M, Zenebe Y, Abera B. Common causes of vaginal infections and antibiotic susceptibility of aerobic bacterial isolates in women of reproductive age attending at Felegehiwot referral Hospital, Ethiopia: A cross sectional study. BMC Women's Health. 2015 5;15(1).

131. Maina AN, Kimani J, Anzala O. Prevalence and risk factors of three curable sexually transmitted infections among women in Nairobi, Kenya. BMC Research Notes. 2016;9(1).

132. Palanee-Phillips T, Schwartz K, Brown ER, Govender V, Mgodi N, Kiweewa FM, et al. Characteristics of women enrolled into a randomized clinical trial of dapivirine vaginal ring for HIV-1 prevention. PLoS ONE. 2015 6;10(6).

133. Kestelyn E, Agaba S, Van Nuil JI, Uwineza M, Umulisa MM, Mwambarangwe L, et al. A randomised trial of a contraceptive vaginal ring in women at risk of HIV infection in Rwanda: Safety of intermittent and continuous use. PLoS ONE. 2018 6;13(6).

134. Donders GGG, Donders F, Bellen G, Depuydt C, Eggermont N, Michiels T, et al. Screening for abnormal vaginal microflora by self-assessed vaginal pH does not enable detection of sexually transmitted infections in Ugandan women. Diagnostic Microbiology and Infectious Disease. 2016 6;85(2):227-30.

135. Nakubulwa S, Kaye DK, Bwanga F, Tumwesigye NM, Mirembe FM. Genital infections and risk of premature rupture of membranes in Mulago Hospital, Uganda: a case control study. BMC Research Notes. 2015 12;8(1):573.

136. Kanyina EW, Kamau L, Muturi M. Cervical precancerous changes and selected cervical microbial infections, Kiambu County, Kenya, 2014: A cross sectional study. BMC Infectious Diseases. 2017 9;17(1).

137. Oliver VO, Otieno G, Gvetadze R, Desai MA, Makanga M, Akelo V, et al. High prevalence of sexually transmitted infections among women screened for a contraceptive intravaginal ring study, Kisumu, Kenya, 2014. International Journal of STD and AIDS. 2018 12;29(14):1390-9.

138. Franceschi S, Chantal Umulisa M, Tshomo U, Gheit T, Baussano I, Tenet V, et al. Urine testing to monitor the impact of HPV vaccination in Bhutan and Rwanda. International Journal of Cancer. 2016 8;139(3):518-26.

139. Francis SC, Holm Hansen C, Irani J, Andreasen A, Baisley K, Jespers V, et al. Results from a cross-sectional sexual and reproductive health study among school girls in Tanzania: High prevalence of bacterial vaginosis. Sexually Transmitted Infections. 2019 5;95(3):219-27.

140. Homsy J, King R, Bannink F, Namukwaya Z, Vittinghof E, Amone A, et al. Primary HIV prevention in pregnant and lactating Ugandan women: A randomized trial. PLOS ONE. 2019 2;14(2):e0212119.

141. Moses E, Pedersen HN, Mitchell SM, Sekikubo M, Mwesigwa D, Singer J, et al. Uptake of community-based, self-collected HPV testing vs. visual inspection with acetic acid for cervical cancer screening in Kampala, Uganda: Preliminary results of a randomised controlled trial. Tropical Medicine and International Health. 2015 10;20(10):1355-67.

142. Chaponda EB, Bruce J, Michelo C, Chandramohan D, Chico RM. Assessment of syndromic management of curable sexually transmitted and reproductive tract infections among pregnant women: an observational cross-sectional study. BMC Pregnancy and Childbirth. 2021 12;21(1):98.

143. Tadesse E, Teshome M, Amsalu A, Shimelis T. Genital Chlamydia trachomatis infection among women of reproductive age attending the gynecology clinic of Hawassa University Referral Hospital, Southern Ethiopia. PLoS ONE. 2016 12;11(12).

144. Masese LN, Wanje G, Kabare E, Budambula V, Mutuku F, Omoni G, et al. Screening for sexually transmitted infections in adolescent girls and young women in Mombasa, Kenya: Feasibility, prevalence, and correlates. Sexually transmitted diseases. 2017 12;44(12):725.

145. Masha SC, Wahome E, Vaneechoutte M, Cools P, Crucitti T, Sanders EJ. High prevalence of curable sexually transmitted infections among pregnant women in a rural county hospital in Kilifi, Kenya. PLoS ONE. 2017 3;12(3).

146. Yuh T, Micheni M, Selke S, Oluoch L, Kiptinness C, Magaret A, et al. Sexually Transmitted Infections Among Kenyan Adolescent Girls and Young Women With Limited Sexual Experience. Frontiers in Public Health. 2020 7;8.

147. Mukanyangezi M, Sengpiel V, Manzi O, Tobin G, Rulisa S, Bienvenu E, et al. Screening for human papillomavirus, cervical cytological abnormalities and associated risk factors in ¡scp¿HIV¡/scp¿ -positive and ¡scp¿HIV¡/scp¿ -negative women in Rwanda. HIV Medicine. 2018 2;19(2):152-66.

148. Maufi AJ, Mazigo HD, Kihunrwa A. Prevalence and factors associated with trichomonas vaginalis infection among pregnant women attending public antenatal clinics in Mwanza City, North-Western Tanzania. Tanzania Journal of Health Research. 2016;18(2).

149. Yegorov S, Galiwango RM, Good SV, Mpendo J, Tannich E, Boggild AK, et al. Schistosoma mansoni infection and socio-behavioural predictors of HIV risk: A cross-sectional study in women from Uganda. BMC Infectious Diseases. 2018 11;18(1).

150. Deese J, Philip N, Lind M, Ahmed K, Batting J, Beksinska M, et al. Sexually transmitted infections among women randomised to depot medroxyprogesterone acetate, a copper intrauterine device or a levonorgestrel implant. Sexually Transmitted Infections. 2021 6;97(4):249-55.

151. Mgodi NM, Takuva S, Edupuganti S, Karuna S, Andrew P, Lazarus E, et al. A Phase 2b Study to Evaluate the Safety and Efficacy of VRC01 Broadly Neutralizing Monoclonal Antibody in Reducing Acquisition of HIV-1 Infection in Women in Sub-Saharan Africa: Baseline Findings. JAIDS Journal of Acquired Immune Deficiency Syndromes. 2021 5;87(1):680-7.

152. Baussano I, Sayinzoga F, Tshomo U, Tenet V, Vorsters A, Heideman DAM, et al. Impact of human papillomavirus vaccination, rwanda and Bhutan. Emerging Infectious Diseases. 2021 1;27(1):1-9.

153. Nsereko E, Uwase A, Mukabutera A, Muvunyi CM, Rulisa S, Ntirushwa D, et al. Maternal genitourinary infections and poor nutritional status increase risk of preterm birth in Gasabo District, Rwanda: a prospective, longitudinal, cohort study. BMC Pregnancy and Childbirth. 2020 6;20(1):345.

154. Masatu ES, Kajura A, Mujuni F, Chibwe E, Nyawale HA, Rambau P, et al. High prevalence of sexually transmitted infections among asymptomatic women opting for the intrauterine contraceptive device use in Mwanza, Tanzania: An urgent call for control interventions. SAGE Open Medicine. 2022;10.

155. Kahsay AG, Mezgebo TA, Gebrekidan GB, Desta BL, Mihretu HG, Dejene TA. Prevalence, Antibiotic Resistance and Associated Factors of Neisseria gonorrhoeae Among Patients Attending Non-Profitable Private Clinics in Mekelle, Tigrai, Ethiopia. Infection and drug resistance. 2023;16:4065-72.

156. Madanitsa M, Barsosio HC, Minja DTR, Mtove G, Kavishe RA, Dodd J, et al. Effect of monthly intermittent preventive treatment with dihydroartemisinin–piperaquine with and without azithromycin versus monthly sulfadoxine–pyrimethamine on adverse pregnancy outcomes in Africa: a double-blind randomised, partly placebo-controlled trial. The Lancet. 2023 3;401(10381):1020-36.

157. Mehta SD, Zulaika G, Agingu W, Nyothach E, Bhaumik R, Green SJ, et al. Analysis of bacterial vaginosis, the vaginal microbiome, and sexually transmitted infections following the provision of menstrual cups in Kenyan schools: Results of a nested study within a cluster randomized controlled trial. PLoS Medicine. 2023 7;20(7 July).

158. Celum CL, Bukusi EA, Bekker LG, Delany-Moretlwe S, Kidoguchi L, Omollo V, et al. PrEP use and HIV seroconversion rates in adolescent girls and young women from Kenya and South Africa: the POWER demonstration project. Journal of the International AIDS Society. 2022 7;25(7):25962.

159. Lokken EM, Jisuvei C, Oyaro B, Shafi J, Nyaigero M, Kinuthia J, et al. Nugent Score, Amsel's Criteria, and a Point-of-Care Rapid Test for Diagnosis of Bacterial Vaginosis: Performance in a Cohort of Kenyan Women. Sexually Transmitted Diseases. 2022 1;49(1):E22-5.

160. Juliana NCA, Deb S, Ouburg S, Chauhan A, Pleijster J, Ali SM, et al. The Prevalence of Chlamydia trachomatis and Three Other Non-Viral Sexually Transmitted Infections among Pregnant Women in Pemba Island Tanzania. Pathogens. 2020 7;9(8):625.

161. Chitneni P, Bwana MB, Owembabazi M, O'Neil K, Kalyebara PK, Muyindike W, et al. Sexually Transmitted Infection Prevalence among Women at Risk for HIV Exposure Initiating Safer Conception Care in Rural, Southwestern Uganda. Sexually Transmitted Diseases. 2020 8;47(8):E24-8.

162. Grabowski MK, Mpagazi J, Kiboneka S, Ssekubugu R, Kereba JB, Nakayijja A, et al. The HIV and sexually transmitted infection syndemic following mass scale-up of combination HIV interventions in two communities in southern Uganda: a population-based cross-sectional study. The Lancet Global Health. 2022 12;10(12):e1825-34.

163. Husen O, Aliyo A, Boru K, Gemechu T, Dedecha W, Ashenafi G. Trichomonas vaginalis and Associated Factors among Pregnant Women Attending Antenatal Care at Bule Hora University Teaching Hospital, Oromia Region, Southern Ethiopia. Journal of Parasitology Research. 2023;2023.

164. Zenebe MH, Mekonnen Z, Loha E, Padalko E. Prevalence, risk factors and association with delivery outcome of curable sexually transmitted infections among pregnant women in Southern Ethiopia. PLoS ONE. 2021 3;16(3 March).

165. Heffron R, Casmir E, Aswani L, Ngure K, Kwach B, Ogello V, et al. HIV risk and pre-exposure prophylaxis interest among women seeking post-abortion care in Kenya: a cross-sectional study. Journal of the International AIDS Society. 2021 5;24(5):25703.

166. McHaro RD, Kisinda A, Njovu L, McHaro M, Mbwilo F, Mihale G, et al. Prevalence of and risk factors associated with HIV, Herpes Simplex Virus-type 2, Chlamydia trachomatis and Neisseria gonorrhoeae infections among 18–24 year old students attending Higher Learning Institutions in Mbeya-Tanzania. PLoS ONE. 2022 5;17(5 May).

167. Nair G, Celum C, Szydlo D, Brown ER, Akello CA, Nakalega R, et al. Adherence, safety, and choice of the monthly dapivirine vaginal ring or oral emtricitabine plus tenofovir disoproxil fumarate for HIV pre-exposure prophylaxis among African adolescent girls and young women: a randomised, open-label, crossover trial. The Lancet HIV. 2023 12;10(12):e779-89.

168. Martin K, Olaru ID, Buwu N, Bandason T, Marks M, Dauya E, et al. Uptake of and factors associated with testing for sexually transmitted infections in community-based settings among youth in Zimbabwe: a mixed-methods study. The Lancet Child and Adolescent Health. 2021 2;5(2):122-32.

169. Nyakambi M, Waruru A, Oladokun A. Prevalence of genital Chlamydia trachomatis among women of reproductive age attending outpatient clinic at Kisumu County Referral Hospital, Kenya, 2021. Journal of Public Health in Africa. 2022 9;13(3).

170. Oware K, Adiema L, Rono B, Violette LR, McClelland RS, Donnell D, et al. Characteristics of Kenyan women using HIV PrEP enrolled in a randomized trial on doxycycline postexposure prophylaxis for sexually transmitted infection prevention. BMC women's health. 2023 12;23(1).

171. van der Veer C, Kondoni C, Kuyere A, Mtonga F, Nyasulu V, Shaba G, et al. Prevalence of sexually transmitted infection in pregnancy and their association with adverse birth outcomes: a case–control study at Queen Elizabeth Central Hospital, Blantyre, Malawi. Sexually Transmitted Infections. 2024.

172. Sineque A, Ceffa S, Parruque F, Guidotti G, Massango C, Sidumo Z, et al. Impact of STIs on cervical cancer screening: Prevalence of ¡i¿Chlamydia trachomatis¡/i¿ and ¡i¿Neisseria gonorrhoeae¡/i¿ in visual inspection with acetic acid (VIA) positive women in Mozambique. International Journal of STD & AIDS. 2024 11;35(13):1019-24.

173. Mbuvi CM, Musila BN, Nyamache AK. Urogenital Infections Among Women Attending Mwingi Hospital, Kitui County, Kenya: Safeguarding Antibiotics Through Microbiological Diagnosis. East Afr Health Res J. 2024;8(1):99-105.

174. Senkoro RH, Juma H, Namkinga L. Prevalence and Risk factors associated with Sexually Transmitted Infections among Women of reproductive age attending reproductive and child health clinics in Dodoma and Dar es Salaam Tanzania. Tanzania Journal of Health Research. 2024;25(1):642-55.

175. Romoren M, Sundby J, Velauthapillai M, Rahman M, Klouman E, Hjortdahl P. Chlamydia and gonorrhoea in pregnant Batswana women: Time to discard the syndromic approach? BMC Infectious Diseases. 2007 4;7.

176. Kleinschmidt I, Rees H, Delany S, Smith D, Dinat N, Nkala B, et al. Injectable progestin contraceptive use and risk of HIV infection in a South African family planning cohort. Contraception. 2007 6;75(6):461-7.

177. Sturm PDJ, Connolly C, Khan N, Ebrahim S, Sturm AW. Vaginal tampons as specimen collection device for the molecular diagnosis of non-ulcerative sexually transmitted infections in antenatal clinic attendees. International Journal of STD & AIDS. 2004 2;15(2):94-8.

178. Paz-Bailey G, Rahman M, Chen C, Ballard R, Moffat HJ, Kenyon T, et al. Changes in the etiology of sexually transmitted diseases in botswana between 1993 and 2002: Implications for the clinical management of genital ulcer disease. Clinical Infectious Diseases. 2005;41(9):1304-12.

179. Pettifor AE, Kleinschmidt I, Levin J, Rees HV, MacPhail C, Madikizela-Hlongwa L, et al. A community-based study to examine the effect of a youth HIV prevention intervention on young people aged 15-24 in South Africa: Results of the baseline survey. Tropical Medicine and International Health. 2005 10;10(10):971-80.

180. Van De Wijgert J, Altini L, Jones H, De Kock A, Young T, Williamson AL, et al. Two methods of self-sampling compared to clinician sampling to detect reproductive tract infections in Gugulethu, South Africa. Sexually Transmitted Diseases. 2006 8;33(8):516-23.

181. Odendaal HJ, Schoeman J, Grove D, de Jager M, Theron G, Orth H, et al. The association between Chlamydia trachomatis genital infection and spontaneous preterm labour. South African journal of obstetrics and gynaecology. 2006;12(3).

182. Sobngwi-Tambekou J, Taljaard D, Nieuwoudt M, Lissouba P, Puren A, Auvert B. Male circumcision and Neisseria gonorrhoeae, Chlamydia trachomatis and Trichomonas vaginalis: Observations after a randomised controlled trial for HIV prevention. Sexually Transmitted Infections. 2009 4;85(2):116-20.

183. Sebitloane HM, Moodley J, Esterhuizen TM. Pathogenic lower genital tract organisms in HIV-infected and uninfected women, and their association with postpartum infectious morbidity. South African medical journal. 2011 6;101(7):466-9.

184. Black V, Magooa P, Radebe F, Myers M, Pillay C, Lewis DA. The detection of urethritis pathogens among patients with the male urethritis syndrome, genital ulcer syndrome and HIV voluntary counselling and testing clients: should South Africa's syndromic management approach be revised? Sexually Transmitted Infections. 2008;84(4):254-8.

185. Lewis DA, Pillay C, Mohlamonyane O, Vezi A, Mbabela S, Mzaidume Y, et al. The burden of asymptomatic sexually transmitted infections among men in Carletonville, South Africa: Implications for syndromic management. Sexually Transmitted Infections. 2008 10;84(5):371-6.

186. De Jongh M, Lekalakala MR, Le Roux M, Hoosen AA. Risk of having a sexually transmitted infection in women presenting at a termination of pregnancy clinic in Pretoria, South Africa. Journal of Obstetrics and Gynaecology. 2010 7;30(5):480-3.

187. Botswana Ministry of Health. Microbiological Survey of Sexually Transmitted Infections 2007-2008. Gaborone; 2011.

188. Thigpen MC, Kebaabetswe PM, Paxton LA, Smith DK, Rose CE, Segolodi TM, et al. Antiretroviral Preexposure Prophylaxis for Heterosexual HIV Transmission in Botswana. New England Journal of Medicine. 2012 8;367(5):423-34.

189. Moodley D, Moodley P, Sebitloane M, Soowamber D, McNaughton-Reyes HL, Groves AK, et al. High prevalence and incidence of asymptomatic sexually transmitted infections during pregnancy and postdelivery in KwaZulu Natal, South Africa. Sexually Transmitted Diseases. 2015 1;42(1):43-7.

190. Kleppa E, Holmen SD, Lillebø K, Kjetland EF, Gundersen SG, Taylor M, et al. Cervical ectopy: Associations with sexually transmitted infections and HIV. A cross-sectional study of high school students in rural South Africa. Sexually Transmitted Infections. 2015 3;91(2):124-9.

191. Peters RPH, Dubbink JH, Van Der Eem L, Verweij SP, Bos MLA, Ouburg S, et al. Cross-sectional study of genital, rectal, and pharyngeal chlamydia and gonorrhea in women in rural South Africa. Sexually Transmitted Diseases. 2014;41(9):564-9.

192. Galappaththi-Arachchige H, Amlie Hegertun I, Holmen S, Qvigstad E, Kleppa E, Sebitloane M, et al. Association of Urogenital Symptoms with History of Water Contact in Young Women in Areas Endemic for S. haematobium. A Cross-Sectional Study in Rural South Africa. International Journal of Environmental Research and Public Health. 2016 11;13(11):1135.

193. Shukla JD, Kleppa E, Holmen S, Ndhlovu PD, Mtshali A, Sebitloane M, et al. The Association Between Female Genital Schistosomiasis and Other Infections of the Lower Genital Tract in Adolescent Girls and Young Women: A Cross-Sectional Study in South Africa. Journal of Lower Genital Tract Disease. 2023 7;27(3):291-6.

194. Jewanraj J, Ngcapu S, Osman F, Ramsuran V, Fish M, Mtshali A, et al. Transient association between semen exposure and biomarkers of genital inflammation in South African women at risk of HIV infection. Journal of the International AIDS Society. 2021 6;24(6):4762.

195. Jongen VW, Schim van der Loeff MF, Botha MH, Sudenga SL, Abrahamsen ME, Giuliano AR. Incidence and risk factors of C. trachomatis and N. gonorrhoeae among young women from the Western Cape, South Africa: The EVRI study. PLOS ONE. 2021 5;16(5):e0250871.

196. Barnabas SL, Dabee S, Passmore JAS, Jaspan HB, Lewis DA, Jaumdally SZ, et al. Converging epidemics of sexually transmitted infections and bacterial vaginosis in southern African female adolescents at risk of HIV. International Journal of STD and AIDS. 2018 5;29(6):531-9.

197. Le Roux MC, Hoosen AA. Quantitative Real-Time Polymerase Chain Reaction for the Diagnosis of Mycoplasma genitalium Infection in South African Men with and Without Symptoms of Urethritis. Sexually Transmitted Diseases. 2017 1;44(1):18-21.

198. Ginindza TG, Stefan CD, Tsoka-Gwegweni JM, Dlamini X, Jolly PE, Weiderpass E, et al. Prevalence and risk factors associated with sexually transmitted infections (STIs) among women of reproductive age in Swaziland. Infectious Agents and Cancer. 2017 5;12(1).

199. Abbai-Shaik NS, Reddy T, Govender S, Ramjee G. Poor Performance of the Chlamydia Rapid Test Device for the Detection of Asymptomatic Infections in South African Men: A Pilot Study . Journal of Sexually Transmitted Diseases. 2016 4;2016:1-3.

200. Huyveneers LEP, Maphanga M, Umunnakwe CN, Bosman-de Boer L, Moraba RS, Tempelman HA, et al. Prevalence, incidence and recurrence of sexually transmitted infections in HIV-negative adult women in a rural South African setting. Tropical medicine & international health : TM & IH. 2023 4;28(4):335-42.

201. Kaida A, Dietrich JJ, Laher F, Beksinska M, Jaggernath M, Bardsley M, et al. A high burden of asymptomatic genital tract infections undermines the syndromic management approach among adolescents and young adults in South Africa: implications for HIV prevention efforts. BMC Infectious Diseases. 2018 10;18(1).

202. Kharsany ABM, McKinnon LR, Lewis L, Cawood C, Khanyile D, Maseko DV, et al. Population prevalence of sexually transmitted infections in a high HIV burden district in KwaZulu-Natal, South Africa: Implications for HIV epidemic control. International Journal of Infectious Diseases. 2020 9;98:130-7.

203. Wynn A, Ramogola-Masire D, Gaolebale P, Moshashane N, Sickboy O, Duque S, et al. Prevalence and treatment outcomes of routine Chlamydia trachomatis, Neisseria gonorrhoeae and Trichomonas vaginalis testing during antenatal care, Gaborone, Botswana. Sexually transmitted infections. 2018 5;94(3):230-5.

204. Gorgens M, Longosz AF, Ketende S, Nkambule M, Dlamini T, Mabuza M, et al. Evaluating the effectiveness of incentives to improve HIV prevention outcomes for young females in Eswatini: Sitakhela Likusasa impact evaluation protocol and baseline results. BMC Public Health. 2020 12;20(1):1591.

205. Gill K, Happel A, Pidwell T, Mendelsohn A, Duyver M, Johnson L, et al. An open-label, randomized crossover study to evaluate the acceptability and preference for contraceptive options in female adolescents, 15 to 19 years of age in Cape Town, as a proxy for HIV prevention methods (UChoose). Journal of the International AIDS Society. 2020 10;23(10).

206. Hoffman CM, Mbambazela N, Sithole P, Morré SA, Dubbink JH, Railton J, et al. Provision of Sexually Transmitted Infection Services in a Mobile Clinic Reveals High Unmet Need in Remote Areas of South Africa: A Cross-sectional Study. Sexually Transmitted Diseases. 2019 3;46(3):206-12.

207. Delany-Moretlwe S, Mgodi N, Bekker LG, Baeten JM, Li C, Donnell D, et al. High prevalence and incidence of gonorrhoea and chlamydia in young women eligible for HIV pre-exposure prophylaxis in South Africa and Zimbabwe: Results from the HPTN 082 trial. Sexually Transmitted Infections. 2023 11;99(7):433-9.

208. Francis SC, Mthiyane TN, Baisley K, Mchunu SL, Ferguson JB, Smit T, et al. Prevalence of sexually transmitted infections among young people in South Africa: A nested survey in a health and demographic surveillance site. PLoS Medicine. 2018 2;15(2):e1002512.

209. Dessai F, Nyirenda M, Sebitloane M, Abbai N. Diagnostic evaluation of the BD Affirm VPIII assay as a point-of-care test for the diagnosis of bacterial vaginosis, trichomoniasis and candidiasis. International Journal of STD and AIDS. 2020 3;31(4):303-11.

210. Govender V, Moodley D, Naidoo M, Connoly C, Ngcapu S, Abdool Karim Q. High incidence of asymptomatic genital tract infections in pregnancy in adolescent girls and young women: Need for repeat aetiological screening. Sexually Transmitted Infections. 2023 11;99(7):482-8.

211. Gray GE, Bekker LG, Laher F, Malahleha M, Allen M, Moodie Z, et al. Vaccine Efficacy of ALVAC-HIV and Bivalent Subtype C gp120–MF59 in Adults. New England Journal of Medicine. 2021 3;384(12):1089-100.

212. Joseph Davey DL, Nyemba DC, Gomba Y, Bekker LG, Taleghani S, DiTullio DJ, et al. Prevalence and correlates of sexually transmitted infections in pregnancy in HIV-infected and- uninfected women in Cape Town, South Africa. PLOS ONE. 2019 7;14(7):e0218349.

213. Naicker M, Dessai F, Singh R, Mitchev N, Tinarwo P, Abbai NS. 'Mycoplasma hominis does not share common risk factors with other genital pathogens': Findings from a South African pregnant cohort. Southern African journal of infectious diseases. 2021 5;36(1).

214. Price MA, Kuteesa M, Oladimeji M, Brumskine W, Edward V, Makkan H, et al. High STI burden among a cohort of adolescents aged 12-19 years in a youthfriendly clinic in South Africa. PLoS ONE. 2024 7;19(7 July).

215. Taku O, Brink A, Meiring TL, Phohlo K, Businge CB, Mbulawa ZZA, et al. Detection of sexually transmitted pathogens and co-infection with human papillomavirus in women residing in rural Eastern Cape, South Africa. PeerJ. 2021 3;9.

216. Chetty R, Mabaso N, Abbai N. Genotypic Variation in ¡i¿Trichomonas vaginalis¡/i¿ Detected in South African Pregnant Women. Infectious Diseases in Obstetrics and Gynecology. 2020 8;2020:1-11.

217. Oree G, Naicker M, Maise HC, Tinarwo P, Ramsuran V, Abbai NS. Tracking Antimicrobial Resistance in ¡i¿Neisseria gonorrhoeae¡/i¿ from the Molecular Level Using Endocervical Swabs. Laboratory Medicine. 2021 1;53(1):18-23.

218. de Voux A, Nyemba DC, Silliman M, Mashele N, Mvududu R, Myer L, et al. Point-of-care testing for sexually transmitted infections and HIV pre-exposure prophylaxis among pregnant women in South Africa, 2021–2022: randomised controlled trial. Sexually Transmitted Infections. 2023 12;100(2):77-83.

219. Mabaso N, Ngobese B, Tinarwo P, Abbai N. Prevalence of Chlamydia trachomatis infection in pregnant women from Durban, South Africa. International Journal of STD & AIDS. 2022;33(10):920–927.

220. Jarolimova J, Chidumwa G, Chimbindi N, Okesola N, Dreyer J, Smit T, et al. Prevalence of Curable Sexually Transmitted Infections in a Population-Representative Sample of Young Adults in a High HIV Incidence Area in South Africa. Sexually Transmitted Diseases. 2023 12;50(12):796-803.

221. Mullick S, Cox LA, Martin CE, Fipaza Z, Ncube S. Comparing the Integration of Syndromic versus Etiological Management of Sexually Transmitted Infections Into HIV Pre-Exposure Prophylaxis Services for Adolescent Girls and Young Women, in South Africa. Journal of Adolescent Health. 2023 12;73(6):S67-72.

222. Mussa A, Wynn A, Ryan R, Babalola CM, Hansman E, Simon S, et al. Prevalence of Chlamydia trachomatis and Neisseria gonorrhoeae infection and associated factors among asymptomatic pregnant women in Botswana. International Journal of STD and AIDS. 2023 6;34(7):448-56.

223. de Voux A, Nyemba DC, Silliman M, Mashele N, Mvududu R, Myer L, et al. Point-of-care testing for sexually transmitted infections and HIV pre-exposure prophylaxis among pregnant women in South Africa, 2021-2022: randomised controlled trial. Sexually transmitted infections. 2024 12;100(2):77-83.

224. Delany-Moretlwe S, Hughes JP, Bock P, Ouma SG, Hunidzarira P, Kalonji D, et al. Cabotegravir for the prevention of HIV-1 in women: results from HPTN 084, a phase 3, randomised clinical trial. The Lancet. 2022 5;399(10337):1779-89.

225. Kelso N, Patterson T. Natural earth free vector and raster map data v1.3; 2024. Available from: `https://www.naturalearthdata.com/`.
